# Subnanomolar MAS-related G protein-coupled receptor-X2/B2 antagonists with efficacy in human mast cells and disease models

**DOI:** 10.1038/s41392-025-02209-8

**Published:** 2025-04-21

**Authors:** Ghazl Al Hamwi, Mohamad Wessam Alnouri, Sven Verdonck, Piotr Leonczak, Shaswati Chaki, Stefan Frischbutter, Pavel Kolkhir, Michaela Matthey, Constantin Kopp, Marek Bednarski, Yvonne K. Riedel, Daniel Marx, Sophie Clemens, Vigneshwaran Namasivayam, Susanne Gattner, Dominik Thimm, Katharina Sylvester, Katharina Wolf, Andreas E. Kremer, Steven De Jonghe, Daniela Wenzel, Magdalena Kotańska, Hydar Ali, Piet Herdewijn, Christa E. Müller

**Affiliations:** 1 PharmaCenter Bonn, Pharmaceutical Institute, Pharmaceutical & Medicinal Chemistry, University of Bonn, An der Immenburg 4, 53121 Bonn, Germany; 2 Medicinal Chemistry, Rega Institute for Medical Research, KU Leuven, Herestraat 49-box 1041, 3000 Leuven, Belgium; 3 Department of Basic & Translational Sciences, School of Dental Medicine, University of Pennsylvania, Philadelphia, PA 19104 USA; 4 Institute of Allergology, Charité—Universitätsmedizin Berlin, corporate member of Freie Universität Berlin and Humboldt-Universität zu Berlin, 12203 Berlin, Germany; 5 Fraunhofer Institute for Translational Medicine and Pharmacology ITMP, Immunology and Allergology, 12203 Berlin, Germany; 6 Department of Systems Physiology, Institute of Physiology, Medical Faculty, Ruhr University of Bochum, 44801 Bochum, Germany; 7 Department of Pharmacological Screening, Jagiellonian University, Medical College, Medyczna 9, 30-688 Krakow, Poland; 8 Department of Medicine 1, University Hospital Erlangen and Friedrich-Alexander-University Erlangen-Nürnberg, Nürnberg, Germany; 9 Helmholtz Institute for Translational Oncology, Mainz (HI-TRON Mainz)—A Helmholtz Institute of the DKFZ, Mainz, Germany; 10 Department of Gastroenterology and Hepatology, University Hospital Zürich, University of Zürich, Zürich, Switzerland; 11 Institute of Physiology I, Life & Brain Center, Medical Faculty, University of Bonn, 53127 Bonn, Germany

**Keywords:** Target validation, Molecular medicine

## Abstract

The MAS-related G protein-coupled receptor-X2 (MRGPRX2), an orphan receptor expressed on mast cells (MCs), is upregulated upon inflammation and induces hypersensitivity and inflammatory diseases. In contrast to the large number of MRGPRX2 agonists, only a few antagonists have been described, and no optimization has been reported to improve potency, selectivity, and drug-like properties. Antagonists with ancillary inhibition of the putative mouse ortholog MRGPRB2 have not been described. Here, we present a multi-disciplinary approach involving chemistry, biology, and computational science, resulting in the development of a small-molecule MRGPRX2 antagonist (PSB-172656, 3-ethyl-7,8-difluoro-2-isopropylbenzo[4,5]imidazo [1,2-*a*] pyrimidin-4(1*H*)-one) based on a fragment screening hit. The compound exhibits metabolic stability, low cytotoxicity, and competitive blockade of MRGPRX2 activation induced by a diverse range of agonists. It displays subnanomolar potency in Ca^2+^ mobilization assays (*K*_i_ value 0.142 nM) and was found to block MRGPRX2-mediated Gα_q_ and Gα_i1_ dissociation, in addition to *β*-arrestin-2 recruitment. PSB-172656 is selective for MRGPRX2 versus all other MRGPRX subtypes. Its effect on MCs was confirmed in cell lines, including rat basophilic leukemia cells (RBL-2H3) recombinantly expressing human MRGPRX2, human Laboratory of Allergic Diseases 2 (LAD2) MCs, and native human skin MCs. PSB-172656 was found to additionally block the putative mouse ortholog of MRGPRX2, MRGPRB2, as determined in Ca^2+^ mobilization assays (*K*_i_ 0.302 nM), and to prevent mouse tracheal contractions, local allergic reactions, and systemic anaphylactic symptoms. PSB-172656 constitutes a unique pharmacological tool and has the potential to be developed as a drug for mast cell-mediated hypersensitivity reactions and chronic inflammatory diseases, addressing a huge unmet medical need.

## Introduction

The increasing prevalence of allergic diseases is a major health challenge worldwide. According to the World Allergy Organization, approximately 30-40% of the global population is affected by one or more allergies, including allergic rhinitis, asthma, atopic dermatitis, and food allergies.^1^ Allergic symptoms can range from mild symptoms, such as sneezing, itching, and rashes, to severe and potentially life-threatening conditions.^1,2^ Allergic or hypersensitivity reactions are categorized into antibody-mediated (types I-III) and cell-mediated subtypes (type IV).^3^ MRGPRX2-mediated reactions, that do not involve immunoglobulin E (IgE), result in direct activation of immune cells, in particular mast cells (MCs).^4^ These hypersensitivity reactions were previously referred to as pseudo-allergic reactions. A recently introduced new classification system of allergic reactions includes tissue-driven allergies (types V and VI) and those induced by direct chemical responses (type VII), with MRGPRX2-associated reactions now reclassified as type VII allergies.^5^

Therapeutic strategies for the treatment of allergies are limited, and new approaches are urgently needed.^2,6,7^ MCs have been established as key effectors in allergic inflammation.^8^ Their physiological functions include induction of mucosal secretion, wound healing, and regulation of innate and adaptive immune responses.^9,10^ MCs are strategically located in the skin and in mucosal barriers.^11^ They are classified based on the protease content of their secretory granules into tryptase-containing MCs (MC_T_) present in lungs and gut, and tryptase/chymase-containing MCs (MC_TC_) present in skin.^12,13^ Upon activation, MCs degranulate and release histamine and other inflammatory mediators, thereby inducing allergic symptoms.^14–16^

MAS-related G protein-coupled receptors (MRGPRs) constitute a large receptor family with 49 members grouped into nine subfamilies based on their sequence similarities, designated MRGPRA, -B, -C, -D, -E, -F, -G, -H, and -X.^17–19^ MRGPRs belong to the δ-branch of rhodopsin-like, class A G protein-coupled receptors (GPCR).^17–19^ The MRGPRX family is primate-specific comprising four orphan receptors, MRGPRX1, -X2, -X3, and -X4, whose cognate agonists are still unknown.^17–19^

MRGPRX2 was claimed to be expressed in dorsal root ganglia (DRG) and was therefore postulated to play a role in pain transmission.^18^ Notably, MRGPRX2 is expressed on MCs, basophils and eosinophils.^20,21^ Under normal conditions, MRGPRX2 appears to be predominantly expressed in skin MC_TC_.^20,21^ Activation of MRGPRX2 was found to play an important role in immune defense, particularly in the initiation of innate immune responses to pathogens. It is activated by cationic molecules, e.g., by many basic peptides, and also by a number of approved drugs at high doses, thereby causing direct, chemical-allergic reactions (type VII).^5,19,22^ Thus, MRGPRX2 has been recognized as an important MC receptor responsible for IgE-independent MC degranulation and hence hypersensitivity reactions (previously called pseudo-allergic or anaphylactoid reactions).^23–25^ MRGPRX2-activating peptides include cortistatin-14,^26^ proadrenomedullin N-terminal peptide-12 (PAMP-12),^27^ PAMP-20,^28^ as well as several antimicrobial host defense peptides, e.g., cathelicidin, LL-37, and *β*-defensins.^29,30^ Recently, the orphan chemokine CXCL14, a 77-mer peptide, and its putative cleavage products, e.g., CXCL14(59-56) and CXCL14(57-56), were found to potently activate MRGPRX2, possibly playing a role in lung inflammation and the development of idiopathic lung fibrosis.^31^

MRGPRX2 agonists have been reported to activate several signaling pathways mediated through all four G protein families, Gα_s_, Gα_i/o_, Gα_q/11_, and Gα_12/13_, with robust coupling to Gα_q_ and Gα_i1_.^32,33^ Gα_q_ proteins stimulate the release of Ca^2+^ from intracellular stores, while Gα_i1_ proteins inhibit adenylate cyclase and thus induce a decrease in intracellular cAMP levels. In addition, MRGPRX2 activation can induce the recruitment of *β*-arrestin-2.^28^

MRGPRX2 has been associated to various diseases, including asthma,^24^ allergic rhinitis,^34^ anaphylaxis, chronic urticaria,^35^ atopic dermatitis,^36^ chronic periodontitis,^37^ idiopathic lung fibrosis,^31^ and further inflammatory diseases.^19^ Thus, MRGPRX2 antagonists have great potential as novel drugs for the treatment of those conditions that are associated with a huge medical need. Although a large number of agonists for MRGPRX2 has been described, only very few MRGPRX2 antagonists are known so far, most of which possess only moderate potency, and a lack of selectivity.^19^

Herein, we present the discovery and development of a unique class of MRGPRX2 antagonists possessing subnanomolar potency, high selectivity, and drug-like properties. We provide evidence for their pharmacological efficacy in blocking human MC degranulation. These compounds are predicted to become indispensable tools for further studying the pathophysiological role of MRGPRX2. Moreover, they may be developed as novel drugs for so far hard-to-treat inflammatory and allergic diseases.

## Results

### Discovery of a unique class of MRGPRX2 antagonists

**Fig. 1:**
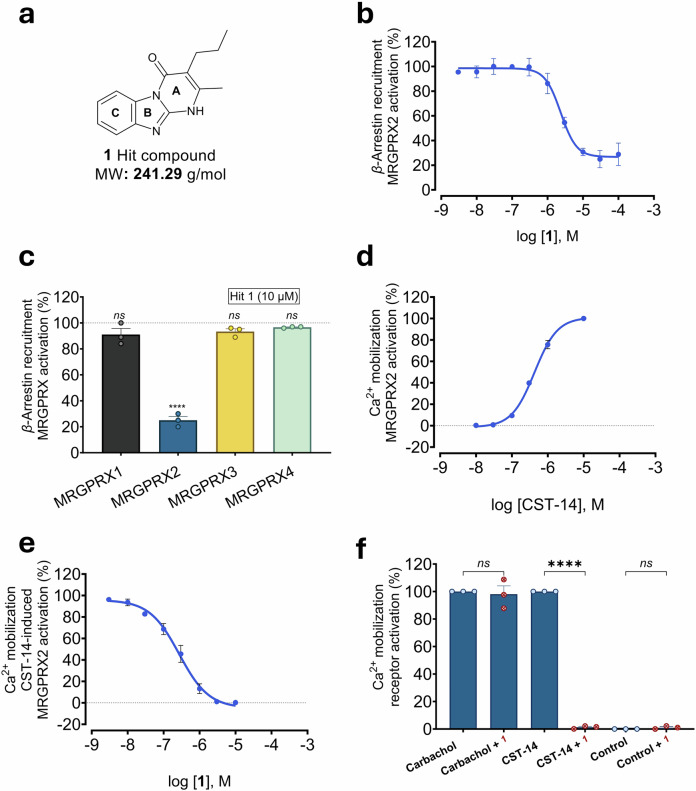
Characterization of MRGPRX2 inhibitor 1. **a** Chemical structure and molecular weight (MW) of **1**. **b** Concentration-response curve of **1** determined in *β*-arrestin recruitment assays. CHO cells that recombinantly express MRGPRX2 were employed versus the agonist CST-14 (at its EC_80_ value of 1.0 µM). An IC_50_ value of 2,420 ± 220 nM was determined for **1**. Data are means ± SEM of 3 biological replicates, performed in duplicates. **c** MRGPRX subtype selectivity of **1** (10 µM) determined in *β*-arrestin assays using CHO cells that recombinantly express the respective MRGPRX subtype. MRGPRX1 was activated by BAM-22P (8.5 µM),^19^ MRGPRX2 was activated by CST-14 (1 µM), MRGPRX3 was activated by PSB-20294 (1 µM), and MRGPRX4 by PSB-18061 (2.6 µM).^44^ Data are means ± SEM of 3 biological replicates, performed in duplicates. The effect of each MRGPRX agonist was assessed in the absence and in the presence of **1**; results were normalized to the maximal agonist effect. Statistical significance was determined by one-way ANOVA (Tukey’s multiple comparisons) tests (*ns*, *P* > 0.05; ****P < 0.0001). **d** CST-14 Concentration-dependent activation of MRGPRX2 in LN229 cells overexpressing MRGPRX2, showing an EC_50_ value of 452 ± 49 nM. Data are means ± SEM of 3 biological replicates, performed in duplicates. **e** Concentration-inhibition curve of **1** determined in Ca^2+^ assays in LN229 cells overexpressing MRGPRX2 versus the agonist CST-14 (at its EC_80_ value of 800 nM), resulting in an IC_50_ value of 683 ± 174 nM. Data are means ± SEM of 3 biological replicates, performed in duplicates. **f** Compound **1** (10 µM) was tested versus the muscarinic agonist carbachol (100 µM) in the LN229-MRGPRX2 cell line, which endogenously expresses muscarinic acetylcholine receptors (mAChRs). Data are means ± SEM of 3 biological replicates. Statistical significance was determined by one-way ANOVA (Tukey’s multiple comparisons) test (*ns* *P* > 0.05 and ****P < 0.0001)

While a variety of MRGPRX2 agonists, including cationic peptides and small molecules, are known, only a few antagonists have been discovered and mainly described in patent literature, without disclosing the structures of the selected clinical candidates.^38–41^ In the search for MRGPRX2 antagonists, we employed a recombinant Chinese hamster ovary (CHO) cell line stably expressing the human MRGPRX2 fused to a small *β*-galactosidase fragment and *β*-arrestin-2 connected to another, larger enzyme fragment. Upon receptor activation, *β*-arrestin is recruited to the cell membrane leading to enzyme complementation.^42^ Addition of a suitable substrate leads to a luminescence signal, while MRGPRX2 antagonists will block the receptor and thus decrease or completely block the signal. This assay was selected for screening because it rarely results in false-positive hits. For receptor activation, we selected the physiological peptide cortistatin-14 (CST-14, PCKNFFWKTFSSCK containing a disulfide bridge, EC_50_ 483 nM, see Supplementary Fig. S1). Screening of part of our compound collection consisting of small fragments and drug-like molecules (molecular weight (MW) < 500 g/mol), mainly focused on purinergic targets, adenosine and nucleotide-activated GPCRs, at a final test concentration of 10 µM versus an EC_80_ of CST-14 (1.0 µM) resulted in the discovery of a unique hit, the heterotricyclic compound 2-methyl-3-propylbenzo[4,5]imidazo[1,2-*a*]pyrimidin-4(1*H*)-one (**1**) (Fig. 1a). Compound **1** is a small molecule with a low MW (241 g/mol), which characterizes it as a fragment,^43^ possessing a chemically tractable scaffold (Fig. 1a). Subsequent determination of a concentration-inhibition curve yielded an IC_50_ value in the low micromolar range (2420 ± 220 nM, Fig. 1b). A maximal inhibition of about 80% was observed, probably due to limited solubility of compound **1** at concentrations above 10 µM, combined with the compound’s moderate potency (Fig. 1b, c). The hit compound was found to be selective for MRGPRX2, showing no inhibition of MRGPRX1, -X3, and -X4 activation (Fig. 1c).

Compound **1** was further validated in a calcium mobilization assay (Ca^2+^ assay) to study its inhibition of Gq protein-dependent signaling. To this end, we utilized a human glioblastoma cell line, LN229, which we had recently discovered to natively express MRGPRX4.^44^ We subsequently investigated the expression of all MRGPRX subtypes in this cell line using quantitative polymerase chain reaction (qPCR). Our results revealed a low level of MRGPRX2 expression (see Supplementary Table S1). Thus, we additionally transfected the cells to stably overexpress human MRGPRX2. In the resulting cell line, CST-14 showed a concentration-dependent MRGPRX2 activation with an EC_50_ value of 452 nM (EC_80_ value of 800 nM), determined by Ca^2+^ assays (Fig. 1d). For hit compound **1**, a *K*_*i*_ value of 246 ± 125 nM was determined (Fig. 1e). Compound **1** (10 µM) did not inhibit the signal induced by carbachol (100 µM), which activates an endogenous muscarinic acetylcholine receptor, indicating that it selectively inhibits MRGPRX2 (Fig. 1f). Compound **1** did not show any agonistic activity, neither in Ca^2+^ assays (Fig. 1f), nor in *β*-arrestin assays (see Supplementary Table S2).

### Structural optimization

**Fig. 2:**
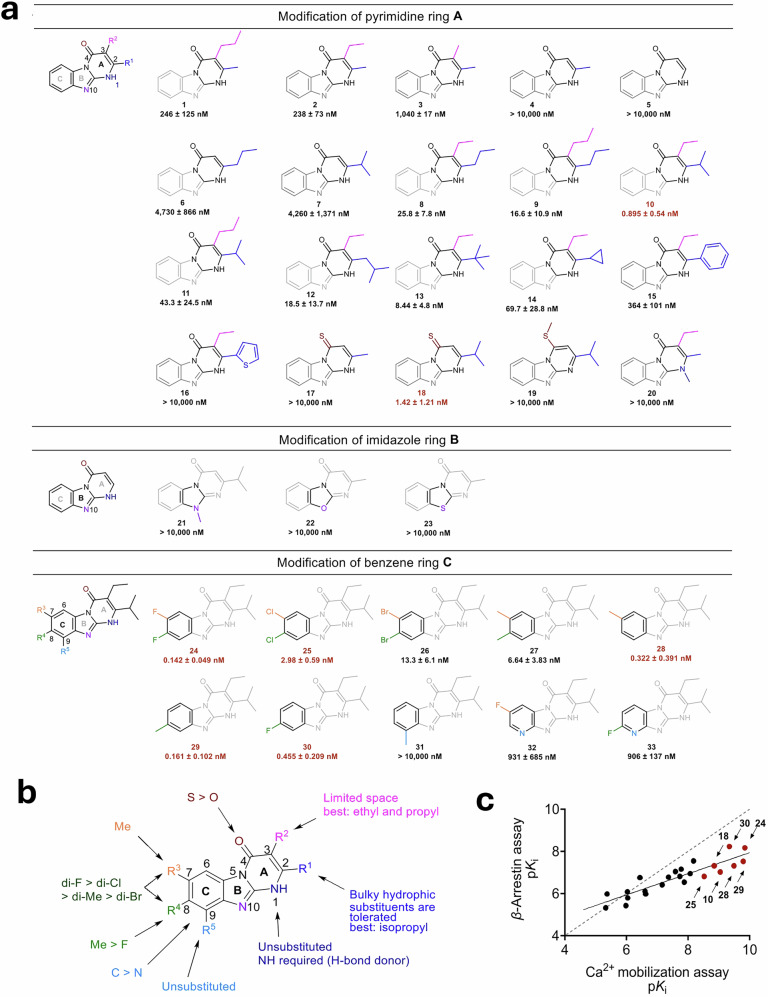
Structural optimization of hit compound 1. **a** Optimization of pyrimido[1,2-*α*]benzimidazole derivative **1** consisting of pyrimidine ring (A), imidazole ring (B), and benzene ring (C). MRGPRX2 inhibition was determined in Ca^2+^ assays performed in LN229 overexpressing MRGPRX2 versus the standard agonist CST-14 (EC_80_ value, 800 nM). Compounds with *K*_i_ values < 5 nM are labeled in red. Data represent mean values ± SEM of 3 biological replicates, performed in duplicates. **b** Summary of the elucidated structure-activity relationships. **c** Correlation plot of p*K*_i_ values determined in *β*‐arrestin assays versus p*K*_i_ values determined in Ca^2+^ mobilization assays for MRGPRX2 antagonists, Pearson correlation coefficient (*r* = 0.896), showing a strong positive correlation significantly different from 0 (two-tailed, *P* < 0.0001). Compounds with more than 50-fold higher potency in Ca^2+^ assay as compared to *β*-arrestin assays are labeled in red. MRGPRX2 inhibition of *β*-arrestin recruitment was determined in *β*-arrestin-CHO cells that recombinantly express MRGPRX2; data are generated from 3 to 4 biological replicates, performed in duplicates

The discovery of the structurally novel, selective MRGPRX2 antagonist **1**, possessing a drug-like, synthetically modifiable structure, enabled us to subsequently tackle its optimization. This was performed in a stepwise optimization process involving a multi-disciplinary approach. All three rings (Fig. 1a) were systematically modified, pyrimidine ring A (Fig. 2a, **1–20**), imidazole ring B (Fig. 2a, **21-23**), and benzene ring C (Fig. 2a, **24-33**). Optimization was achieved by close interdisciplinary collaboration of chemists, biologists, and computational scientists, initially pursuing a potency-driven design approach (for details on synthetic procedures, see Supplementary Material 1). This concerted integrative approach enabled the development of the first subnanomolar MRGPRX2 inhibitor, endowed with drug-like properties, within only a few optimization cycles.

The new compounds were evaluated in Ca^2+^ assays utilizing MRGPRX2-overexpressing LN229 cells to determine structure-activity relationships (SARs). The previously described MRGPRX2 antagonist quercetin was included for comparison, displaying an IC_50_ value of 46,100 nM (Ca^2+^ assay), and of 71,200 nM (*β*-arrestin assay), respectively (Supplementary Table S2). These values are well in agreement with the reported potency of quercetin previously determined in Ca^2+^ assays in Laboratory of Allergic Diseases 2 (LAD2) MCs.^45^

The new lead structure **1** features a methyl group in the 2-position and a propyl group in the 3-position (Fig. 2a, *K*_i_ **246** nM, Ca^2+^ assay). A series of compounds with structural variations of these substituents, R^1^ (2-position) and R^2^ (3-position), on the pyrimidine ring (A) was subsequently synthesized. Keeping a methyl group at the 2-position and substituting the 3-position with an ethyl group was well-tolerated (**2,** *K*_i_ **238** nM), while its replacement by a smaller methyl group resulted in a 4-fold decrease in potency (**3,** *K*_i_ **1040** nM). Compounds **4** and **5**, lacking a substituent in the 3-position, were inactive. Sterically more demanding alkyl groups such as propyl (**6,** *K*_i_ **4730** nM) and isopropyl (**7,** *K*_i_ **4260** nM) in the 2-position of compound **5** resulted in low potency (Fig. 2a). These findings indicated that an alkyl group in the 3-position likely fills a hydrophobic pocket in the receptor, which is of restricted size, well accommodating ethyl or propyl. In the 2-position, a bulkier residue appeared to be more favorable. Combining two structural variations by fixing position 3 as ethyl or propyl and increasing the steric bulk at position 2 led to a series of potent antagonists (**8**-**15**). The rank order of potency for 3-ethyl derivatives was as follows, regarding the 2-substituent: isopropyl (**10,** *K*_i_ **0.895** nM) > *tert*-butyl (**13,** *K*_i_ **8.44** nM) > propyl (**8,** *K*_i_ **25.8** nM) > cyclopropyl (**14,** *K*_i_ **69.7** nM) > phenyl (**15,** *K*_i_ **364** nM) > 2-thienyl (**17**, inactive). Thus, isopropyl in the 2-position combined with ethyl in the 3-position was optimal (**10,** *K*_i_ **0.895** nM), showing a 275-fold increase in potency compared to the parent compound **1** (Fig. 2a, b).

The exchange of the carbonyl function for a thione in position 4 of antagonist **7,** yielding **18,** proved to be beneficial, showing an almost 3000-fold increase in potency (**7,** *K*_i_ **4260** nM; **18,** *K*_i_ **1.42** nM). In contrast, the corresponding thioether **19** was completely inactive, likely due to the loss of the hydrogen bond donor at position 1 (Fig. 2a, b). Methylation of the *N*1- (**20**) or *N*10-position (**21**) of the benzimidazole core structure was not tolerated, again confirming that the NH function is crucial for interaction with MRGPRX2, presumably due to forming an essential hydrogen bond. Consequently, the exchange of *N*10 for O or S also resulted in inactive analogs **22** and **23**, again underscoring the necessity of an H-bond donor (Fig. 2a, b).

Fluorination at the 7- and 8-positions caused a 7-fold increase in potency (**24,** *K*_i_ **0.142** nM) compared to the so far best antagonist **10** (*K*_i_ **0.895** nM), corresponding to an 1870-fold increase over the hit compound **1**. Larger halogen atoms at the 7- and 8-position, such as chlorine (in **25,** *K*_i_ **2.98** nM) or bromine (in **26,** *K*_i_ **13.3** nM), resulted in decreased potency. Fluorine, with a smaller van der Waals radius and a higher electronegativity than chlorine and bromine, appears to have the optimal size for lipophilic interaction within the binding pocket. The corresponding 7,8-dimethyl derivative **27** was 5-fold less potent than the di-fluoro-substituted **24** (**27,** *K*_i_ **6.64** nM), likely due to the larger size of the methyl groups. However, it is remarkable that halogens are not required, and methyl groups are well-tolerated, indicating that the lipophilicity is crucial rather than the electronegativity of the halogen atoms (Fig. 2a).

Introducing a single methyl group into the 7-position (**28,** *K*_i_ **0.322** nM) or the 8-position (**29,** *K*_i_ **0.161** nM) of **11** improved the potency by 20- or 40-fold compared to the 7,8-dimethyl derivative **27** (*K*_i_ **6.64** nM). Having two methyl groups may introduce steric hindrance or disrupt the optimal alignment and interactions within the binding pocket. Methylation at the 8-position was shown to be slightly more favored as compared to 7-methyl-substitution (Fig. 2a, b). Since the combination (7,8-dimethyl substitution) is much less potent, a slightly different binding mode of **28** as compared to **29** is likely. Mono-fluorination at the 8-position of the unsubstituted **10** (*K*_i_ **0.895** nM) did not cause a significant change in potency (**30,** *K*_i_ **0.455** nM). It should be noted that the 7,8-difluoro derivative **24** (*K*_i_ **0.142** nM) displayed about 4-fold higher potency compared to the 8-monofluorinated derivative.

A methyl group at the 9-position of **31** abolished antagonistic activity (Fig. 2a, b). Replacement of a methine by a nitrogen atom at the 9-position, which chemically allowed the straightforward introduction of a single fluorine substituent into the 7- or 8-position, respectively, resulting in pyridine derivatives **32** and **33**, also reduced potency dramatically.

The tricyclic benzimidazoles were further tested in *β*-arrestin recruitment assays, and IC_50_ and *K*_i_ values were determined for all potent antagonists (Supplementary Table S2). A strong correlation between p*K*_i_ values of the inhibitors obtained in both assays, Ca^2+^ mobilization, and *β*-arrestin recruitment, was calculated with a correlation coefficient (*r*) of 0.866 (Fig. 2c). It was, however, observed that the compounds exhibited in most cases higher potency in the Ca^2+^ assay than in the *β*-arrestin assay. This was particularly true for the most potent antagonists displaying *K*_i_ values below 5 nM. This striking difference is likely attributed to plasma protein binding of the compounds; in fact, the *β*-arrestin assay medium contained plasma proteins, while Ca^2+^ assays were performed in pure buffer solution. Subsequent studies confirmed high plasma protein binding of the compounds (Supplementary Table S3).

The most potent antagonist of the present series, 7,8-difluoro-substituted **24** (PSB-172656), was further characterized. It showed relatively high metabolic stability when incubated with human liver microsomes, indicating a low intrinsic clearance (Cl_int_) of 15 µL/min/mg, corresponding to an estimated half-life of 92 min (see Supplementary Table S3). The 8-mono-fluoro-substituted analog **30** was much less stable (half-life of 14 min), suggesting that the second fluorine (7-F) blocks a potential metabolization site, probably preventing ring hydroxylation. Interestingly, the 8-fluoropyridyl derivative **33** showed higher metabolic stability (half-life 48.8 min). The 7,8-dimethyl-analog **27** was again found to be unstable (half-life 10.6 min), presumably because the methyl groups are susceptible to enzymatic hydroxylation.

Potential cytotoxicity of **24** and its similarly potent analogs **27** and **30** was assessed in 3-(4,5-dimethylthiazol-2-yl)-2,5-diphenyl-2*H*-tetrazolium bromide (MTT) assays performed on human 1321N1 astrocytoma cells (Supplementary Table S3). This cell line was chosen since it is well-established for the screening of cytotoxic drugs, making it a reliable choice for assessing cytotoxicity.^46^ None of the investigated MRGPRX2 antagonists affected cell viability when tested at concentrations up to 10 µM, which is at least 500-fold higher than their *K*_i_ value in blocking MRGPRX2 activation as determined in Ca^2+^ assays (Supplementary Table S3).

Thus, the optimized compound **24** (PSB-172656) is an extraordinarily potent, non-cytotoxic, metabolically stable, drug-like MRGPRX2 antagonist, which should be suitable for future in vitro and in vivo studies.

### Comprehensive biological evaluation of PSB-172656

**Fig. 3:**
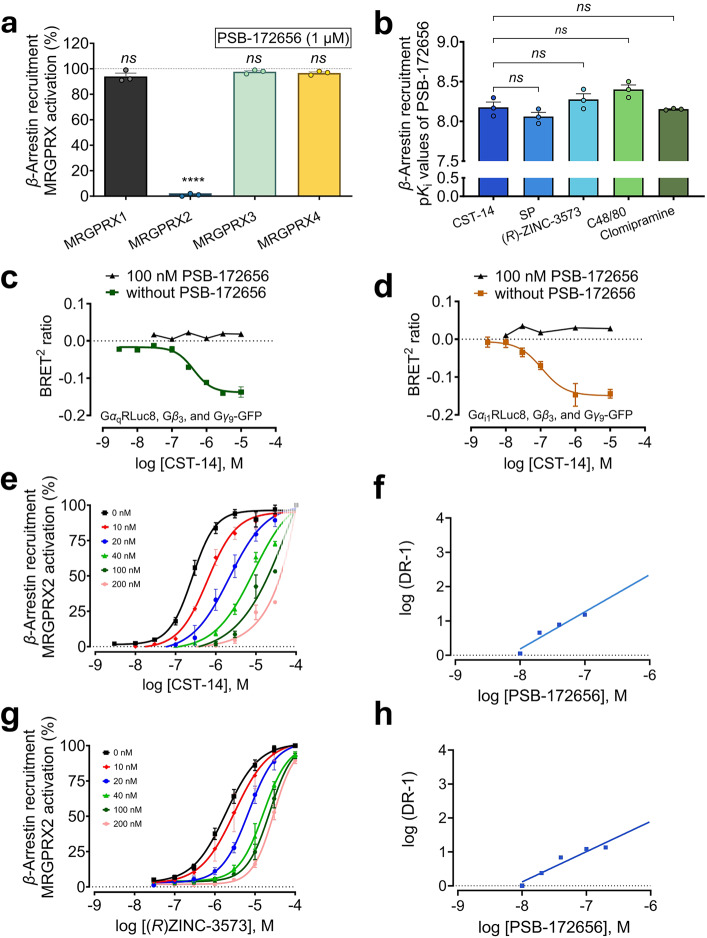
Biological characterization of PSB-172656. **a** Selectivity of PSB-172656 (100 nM) for MRGPRX2 over MRGPRX1, MRGPRX3, and MRGPRX4 measured in *β*-arrestin assays utilizing *β*-arrestin-CHO cells with recombinant expression of the respective receptor. MRGPRX1 was activated by BAM-22P (8.5 µM),^19^ MRGPRX2 was activated by CST-14 (1 µM), MRGPRX3 was activated by PSB-20294 (10 µM), MRGPRX4 was activated by PSB-18061 (2.6 µM).^44^ Data represent mean values ± SEM of 3-4 biological replicates, performed in duplicates. Statistical significance was determined by one-way ANOVA (Tukey’s multiple comparisons) test (*ns* *P* > 0.05 and ****P < 0.0001), comparing the effect of each MRGPRX agonist in the presence of PSB-172656 (10 µM) versus their maximal effects (100%) in the absence of the antagonist. **b** p*K*_i_ values of PSB-172656 determined using *β*-arrestin recruitment assays versus structurally diverse MRGPRX2 agonists, versus the peptide agonist SP (EC_80_ 3 µM); the small-molecule agonist (*R*)-ZINC-3573 (EC_80_ 2 µM); C48/80 (EC_80_ 18 µM); the tricyclic antidepressant and MRGPRX2 agonist clomipramine (EC_80_ 13 µM), and CST-14 as a control. Data are means ± SEM of 3 biological replicates, in duplicates. Statistical significance was determined by one-way ANOVA (Tukey’s multiple comparison) test (*ns* *P* > 0.05). For concentration-response curves and structures, see Supplementary Fig. S2. **c** TRUPATH G*α*_q_ dissociation assays in LN229 glioblastoma cells recombinantly expressing MRGPRX2 and G*α*_q_RLuc8, Gβ_3_, and Gγ_9_-GFP2,^47^ concentration-response curve of the MRGPRX2 peptide agonist CST-14 showing an EC_50_ value of 499 ± 207 nM. This effect was completely inhibited after the preincubation of PSB-172656 (100 nM). **d** TRUPATH G*α*_i1_ dissociation assays in LN229 glioblastoma cells recombinantly expressing MRGPRX2 and G*α*_i1_RLuc8, Gβ_3_, and Gγ_9_-GFP2, concentration-response curve of the MRGPRX2 peptide agonist CST-14 showing an EC_50_ value of 113 ± 16 nM. CST-14-induced G*α*_i1_ dissociation was completely inhibited after preincubation with PSB-172656 (100 nM). Data are means ± SEM of 3 biological replicates, performed in duplicates. **e** Shift assay to determine inhibition type of PSB-172656 versus CST-14. EC_50_ values: without antagonist: 283 ± 45 nM; with 10 nM PSB-172656: 601 ± 89 nM; with 20 nM PSB-172656: 1580 ± 403 nM; with 40 nM PSB-172656: 2510 ± 889 nM; with 100 nM PSB-172656: 4,590 ± 1,580 nM; with 200 nM PSB-172656: 6260 ± 113 nM, faint dashed lines indicate extrapolation. Extrapolated data were not used for analysis but only shown for a comprehensive view. **f** Schild plot for the effect of PSB-172656 on the concentration-response curves for CST-14. *K*_B_ value of 6.71 nM (slope,1.08; X-intercept, -8.17 coresponding to -p*K*_B_ value). **g** Inhibition mode of PS_B_-172656 versus (*R*)-ZINC-3573. EC_50_ values: without antagonist: 2020 ± 333 nM; with 10 nM PSB-172656: 4060 ± 1560 nM; with 20 nM PSB-172656: 6780 ± 572 nM; with 40 nM PSB-172656: 15,900 ± 4020 nM; with 100 nM PSB-172656: 26,300 ± 49,10 nM; with 200 nM PSB-172656: 29,400 ± 4550 nM. **h** Schild plot for the effect of PSB-172656 on the concentration-response curves for (*R*)-ZINC-3573. *K*_B_ value: 7.59 nM (slope, 0.891; X-intercept, -8.17 corresponding to -p*K*_B_ value). Data represent means ± SEM of 3-6 biological replicates

Selectivity of the most promising MRGPRX2 antagonist **24** (PSB-172656) was subsequently studied in *β*-arrestin-CHO cell lines expressing one of the MRGPRX family members, MRPGRX1, -X3, or -X4. Even at a high concentration of 10 µM, PSB-172656 was not able to block any of the other MRGPRX subtypes (Fig. 3a), indicating high selectivity of at least 1000-fold, while it completely blocked MRGPRX2 activation.

MRGPRX2 can be activated by structurally diverse agonists, small molecules as well as peptides.^19,22^ Thus, we tested antagonist PSB-172656 in *β*-arrestin assays versus a variety of structurally diverse MRGPRX2 agonists (employed at their EC_80_ values). PSB-172656 not only inhibited MRGPRX2 activation induced by the peptide CST-14 (*K*_i_ 6.81 nM), but also by all other investigated agonists including the peptide substance P (SP; *K*_i_ 8.82 nM, Supplementary Fig. S2a), the small molecules (*R*)-ZINC-3573 (*K*_i_ 5.44 nM, Supplementary Fig. S2b) and clomipramine (IC_50_ *K*_i_ 7.04 nM, Supplementary Fig. S2c), and the polymeric compound 48/80 (C48/80; *K*_i_ 3.68 nM, Supplementary Fig. S2d). The potency of PSB-172656 in blocking MRGPRX2 activation was almost identical ( < 2-fold difference from the *K*_i_ value of PSB-172656 versus CST-14) for all of the investigated agonists (Fig. 3b).

To further investigate PSB-172656 signaling, direct activation of the relevant G protein subunits, Gα_q_ and Gα_i1_,^32^ was studied. This was assessed using G protein dissociation assays and LN229 cells stably overexpressing MRGPRX2 while transiently expressing the investigated G protein (Fig. 3c, d).^47^ In fact, PSB-172656 (100 nM) completely inhibited MRGPRX2-induced Gα_q_ as well as Gα_i1_ protein activation (Fig. 3c, d).

Next, the mechanism of MRGPRX2 blockade by PSB-172656 (orthosteric or allosteric) was investigated with respect to the peptide agonist CST-14 and versus the small-molecule agonist *(R)*-ZINC-3573. To this end, curves of CST-14 and *(R)*-ZINC-3573 were recorded in the presence of increasing concentrations of antagonist PSB-172656 utilizing *β*-arrestin recruitment assays (Fig. 3e). A parallel shift of the concentration-response curve was observed in the presence of low concentrations of the antagonist (up to 20 nM) versus the peptidic agonist CST-14 (Fig. 3e). The limited solubility of CST-14 prevented the use of higher concentrations and thus, the curves appear to be cut off (Fig. 3e). However, the Schild plot at the lower antagonist concentrations was linear with a slope of 1.08 resulting in a *K*_B_ value of 6.71 nM (p*K*_B_ value 8.17) (Fig. 3e, f). This indicates a competitive mode of action. Despite the large molecular size of CST-14 (14 amino acids), the small, heterocyclic antagonist PSB-172656 probably interacts with similar residues in the binding pocket. In the case of the small-molecule agonist (*R)*-ZINC-3573, higher concentrations could be employed since this compound is well water-soluble (Fig. 3g). Increasing concentrations of PSB-172656 induced a parallel right-ward shift of the curve without any significant reduction in efficacy. This is indicative of a competitive mode of action. The Schild plot for the inhibition of (*R)*-ZINC-3573-induced receptor activation by PSB-172656 was linear with a slope of 0.891, and a *K*_B_ value of 7.59 nM was calculated (p*K*_B_ value 8.13) (Fig. 3g, h).

### Docking studies

To predict the binding pose and interactions of the new MRGPRX2 antagonists, we docked PSB-172656 into our optimized homology model (see Supplementary Material 2).^31^ Recently, cryogenic electron microscopy (cryo-EM) structures of MRGPRX2 in complex with agonists, the peptide CST-14, and the small molecule (*R*)ZINC-3573, were reported.^32^ Thus, in addition to the optimized homology model, we docked PSB-172656 into the cryo-EM structure (PDB: 7S8L). The docking studies predicted virtually the same orientation of PSB-172656 within the binding pocket of the optimized homology model and that of the cryo-EM structure, with an overall root-mean-square deviation (RMSD) value of 2.77, suggesting that the model captures the essential features of the reference structure reasonably well (see Supplementary Fig. S3).

**Fig. 4:**
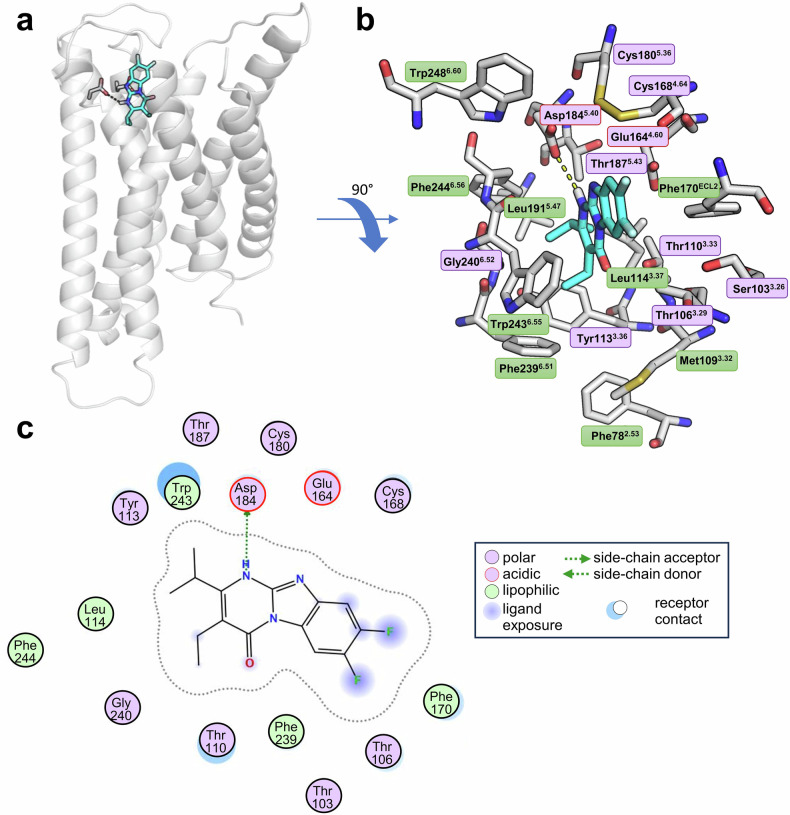
Molecular docking study. **a** Docked pose of PSB-172656 (carbon-colored cyan) into the putative orthosteric binding pocket of the human MRGPRX2 (PDB: 7S8L). The MRGPRX2 model is shown in cartoon representation with the binding pocket near the surface of the receptor (side-view). **b** The binding pocket and PSB-172656 are shown as stick models (top-view). The oxygen atoms are colored in red, nitrogen atoms in blue, and fluorine atoms in cyan, polar amino acids are indicated in purple and lipophilic amino acids are depicted in green. **c** 2D interaction diagram of the docking pose of PSB-172656 in the binding site of MRGPRX2

According to the docking study, PSB-172656 binds to a shallow pocket of MRGPRX2 (Fig. 4a). As shown in Fig. 4a, the N1-H of PSB-172656 is proposed to form a hydrogen bond with Asp184^5.38^ within the binding pocket. The compound is likely stabilized inside the pocket through hydrophobic interactions of the alkyl groups in positions 2 and 3 (ethyl and isopropyl, respectively) with the amino acid residues Tyr113^3.36^, Leu114^3.36^, Phe239^6.51^, and Trp243^6.55^ (Fig. 4b, c). Replacement of these two substituents by methyl groups in compound **3** resulted in reduced potency (Fig. 2a–c), possibly due to a change in the binding orientation, both compounds adopting different binding modes. The carbonyl function at position 4 is exposed and likely forms a water-mediated hydrogen bond with the free hydroxyl group of Thr110^3.33^ (Fig. 4b, c). According to the model, the fluorine atoms in positions 7 and 8 are surrounded by polar residues including Ser103^3.26^, Thr106^3.29^, Thr107^3.30^, and Glu164^4.60^, possibly forming direct or water-mediated electrostatic interactions (Fig. 4b, c). Additionally, the benzene ring of PSB-172656 is likely to form a hydrophobic interaction with Phe170^ECL2^ (Fig. 4b, c).

It should be noted that the recent cryo-EM structures of MRGPRX2 in complex with CST-14 and (*R)*-ZINC-3573 indicate that Glu164^4.60^, Phe170^ECL2^, Asp184^5.38^, and Trp243^6.55^ in the binding pocket are crucial for agonist-receptor interactions, as confirmed by mutagenesis studies.^32^ These amino acid residues are predicted to also interact with the new potent antagonist PSB-172656 based on our docking studies. These results are consistent with the characterization of PSB-172656 as a competitive antagonist interacting with the same binding site as the reported agonists.

### Effects of PSB-172656 on mast cells

**Fig. 5:**
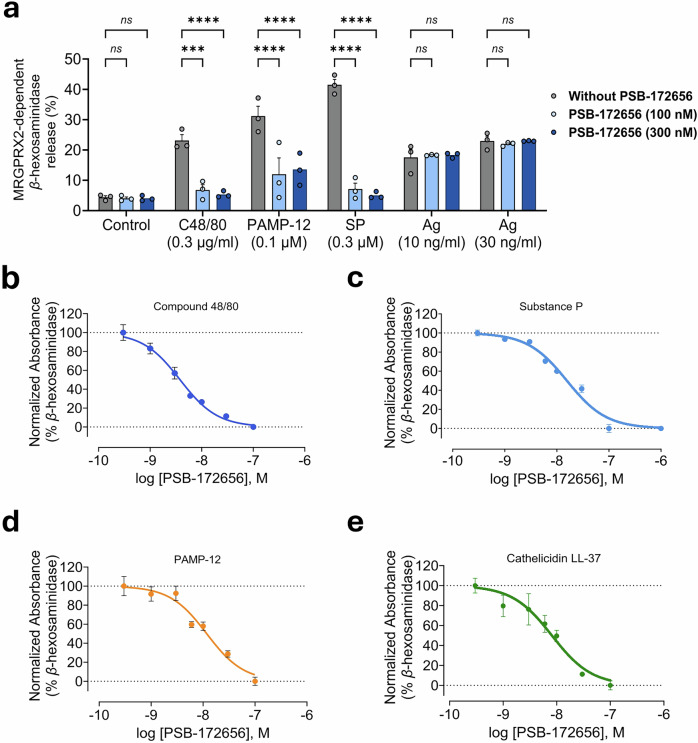
PSB-172656 blocks MRGPRX2-induced mast cell degranulation without affecting IgE-mediated responses. **a** RBL-MRGPRX2 cells were incubated with control (vehicle) or preincubated with PSB-172656 (100 or 300 nM), followed by stimulation with C48/80 (0.3 µg/mL), substance P (SP; 0.3 µM), or PAMP-12 (0.1 µM), and *β*-hexosaminidase release was measured. RBL-MRGPRX2 cells were sensitized with monoclonal anti-dinitrophenyl antibody (DNP-specific IgE), and degranulation induced by 2,4-dinitrophenylated bovine serum albumin (DNP-BSA, 10 and 30 ng/mL) was measured without or after preincubation with PSB-172656 (0.1 and 0.3 µM). For concentration-response curves, RBL-MRGPRX2 cells were treated with PSB-172656 (various concentrations) for 5 min, followed by activation with **b** C48/80 (0.3 µg/mL) (PSB-172656, IC_50_ 3.79 nM), **c** substance P (SP, 0.3 µM) (PSB-172656, IC_50_ 15.5 nM), **d** PAMP-12 (0.1 µM) (PSB-172656, IC_50_ 11.7 nM), or **e** cathelicidin LL-37 (3 µM) (PSB-172656, IC_50_ 8.12 nM), for 30 min, and *β*-hexosaminidase release was determined. Data are expressed as means ± SEM of 3 biological replicates. Statistical significance was determined by two-way ANOVA (Tukey’s multiple comparisons)

Having a competitive MRGPRX2 antagonist with subnanomolar potency in hand, we next studied its effects on MCs. Activation of MRGPRX2, which is highly expressed in skin MCs, results in degranulation and histamine release.^19^ We therefore investigated whether PSB-172656 inhibits MRGPRX2-mediated MC activation utilizing the rat MC line RBL-2H3 recombinantly expressing the human MRGPRX2 (designated RBL-MRGPRX2). These cells were treated with two different concentrations of PSB-172656 (100 nM and 300 nM), and then exposed to different MRGPRX2 agonists, namely C48/80 (0.3 µg/mL), SP (0.3 µM), and PAMP-12 (0.1 µM), respectively. Degranulation of the cells was assayed by *β*-hexosaminidase release measurements. At both tested concentrations, PSB-172656 (100 nM and 300 nM) completely abolished MC degranulation in response to C48/80 and SP, while it substantially inhibited the response to PAMP-12 (Fig. 5a). The effect was MRGPRX2-specific since PSB-172656 did not inhibit IgE-mediated degranulation induced by 2,4-dinitrophenylated bovine serum albumin (DNP-BSA) of MCs sensitized with DNP-specific mouse IgE (SPE-7; 1 µg/mL) (Fig. 5a). PSB-172656 inhibited degranulation in response to all of the investigated MRGPRX2 agonists in a concentration-dependent manner with similarly high IC_50_ values: C48/80 (3.79 nM, Fig. 5b), SP (15.5 nM, Fig. 5c), PAMP-12 (11.7 nM, Fig. 5d), and LL-37 (8.12 nM, Fig. 5e).

**Fig. 6:**
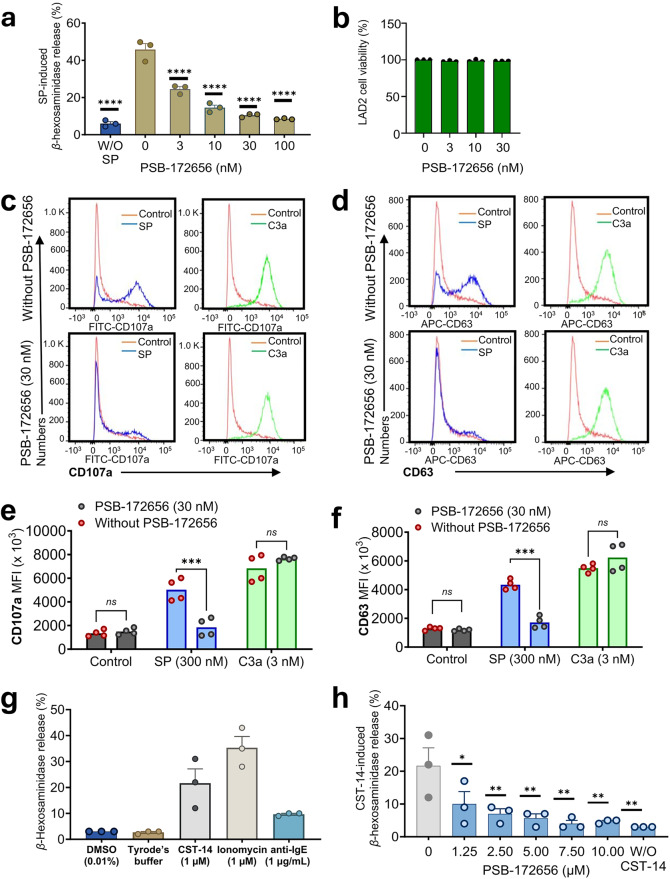
PSB-172656 inhibits MRGPRX2-mediated mast cell degranulation. **a** LAD2 cells were incubated with buffer (control) or various concentrations of MRGPRX2 antagonist PSB-172656, subsequently exposed to substance P (SP, 0.3 µM), and *β*-hexosaminidase release was measured. PSB-172656 showed an IC_50_ value of 5.26 ± 0.18 nM. Data are expressed as means ± SEM of 3 biological replicates. Statistical significance was determined by one-way ANOVA (Tukey’s multiple comparisons), compared to the effect of SP in the absence of PSB-172656 (*P*-value*** < 0.001). **b** Potential cytotoxicity of PSB-172656 was determined in 3-[4,5-dimethylthiazol-2-yl]-2,5-diphenyl tetrazolium bromide (MTT) assays performed on LAD2 cells. Cells were treated with different concentrations of PSB-172656 for 24 h at 37 °C, and the optic density value was determined at 570 nm (the viability of untreated cells corresponds to 100% cell viability). Data are means of 3 biological replicates. To assess degranulation by flow cytometry, LAD2 cells were preincubated with buffer or with PSB-172656 (30 nM, 5 min), activated for 5 min with SP (0.3 μM) or C3a (3 nM), and representative histograms of the expression of **c** CD107a, and **d** CD63 on the cell surface were determined. Mean fluorescence intensity (MFI) levels of **e** CD107a and **f** CD63 are depicted. Data are presented as means ± SEM of 4 biological replicates. **g** *β*-Hexosaminidase release assay of primary human skin MCs ex vivo (*n* = 3 donors). The cells were incubated for 1 h with CST-14 (1 µM), Tyrode’s buffer, vehicle (DMSO 0.01%), anti-IgE (1 μg/mL) or ionomycin (1 µM). Degranulation is characterized by the release of β-hexosaminidase, expressed as a percentage of the total content. **h** Human skin MCs were incubated with different concentrations of PSB-172656 for 30 min followed by stimulation with CST-14 (1 µM). Statistical significance was calculated by one-way ANOVA (Tukey’s multiple comparisons), compared to agonist without PSB-172656 (*P*-value* < 0.05, *** < 0.01, and *P*-value**** < 0.001)

To evaluate the antagonist in an even more physiological system, we further performed experiments using the well-established human LAD2 cell line, a human MC line that exhibits an endogenous expression of MRGPRX2.^48^ PSB-172656 inhibited MRGPRX2-mediated Ca^2+^ mobilization in LAD2 cells (see Supplementary Fig. S4). Similarly, as in the recombinant RBL-MRGPRX2 MC line, PSB-172656 caused a dose-dependent inhibition of *β*-hexosaminidase release in LAD2 cells in response to SP, displaying an IC_50_ value of 5.26 nM (Fig. 6a). PSB-172656 did not exhibit toxicity even at concentrations 6-fold higher than its IC_50_ value (Fig. 6b).

The granule-associated proteins CD107a and CD63 externalize in the process of degranulation. Their quantification by flow cytometry is a reliable marker of mast cell activation, which is often used for the diagnosis of allergies and anaphylaxis in mast cell activation tests.^49,50^ Thus, the expression of CD107a and CD63 on the cell surface was determined to confirm the effects of PSB-172656 on SP-induced MRGPRX2-mediated degranulation. The MRGPRX2 agonist SP (300 nM) resulted in an increased expression of CD107a and CD63 on the cell surface as determined by flow cytometry, which was completely prevented by a low concentration of PSB-172656 (30 nM, 5 min) (Fig. 6c, d and Supplementary Fig. S5). In addition to MRPGRX2, LAD2 cells also express a GPCR that is specifically activated by the complement component C3a.^51^ Both receptors can activate Gα_i/o_ proteins to promote MC activation.^48,52^ To further determine the specificity of PSB-172656 in blocking MRGPRX2, we preincubated LAD2 cells with PSB-172656 (30 nM) and tested its ability to modulate the effect of C3a (3 nM) on the expression of CD107a and CD63 on the cell surface. In contrast to its blockade of SP effects, PSB-172656 did not affect the C3a-induced response (Fig. 6e, f).

Finally, the effect of the MRGPRX2 antagonist PSB-172656 on MRGPRX2-mediated MC degranulation in primary human skin mast cells isolated from breast skin tissue was investigated. These cells were controlled by flow cytometry for CD117, FcεRI, and MRGPRX2 to ensure their purity (Supplementary Fig. S6), and their functionality was assessed using the MC degranulation *β*-hexosaminidase release assay (Fig. 6g). PSB-172656 inhibited MC degranulation in response to CST-14 exposure (1 µM) in a concentration-dependent manner (Fig. 6h).

### Effects of PSB-172656 on mouse MRGPRB2

**Fig. 7:**
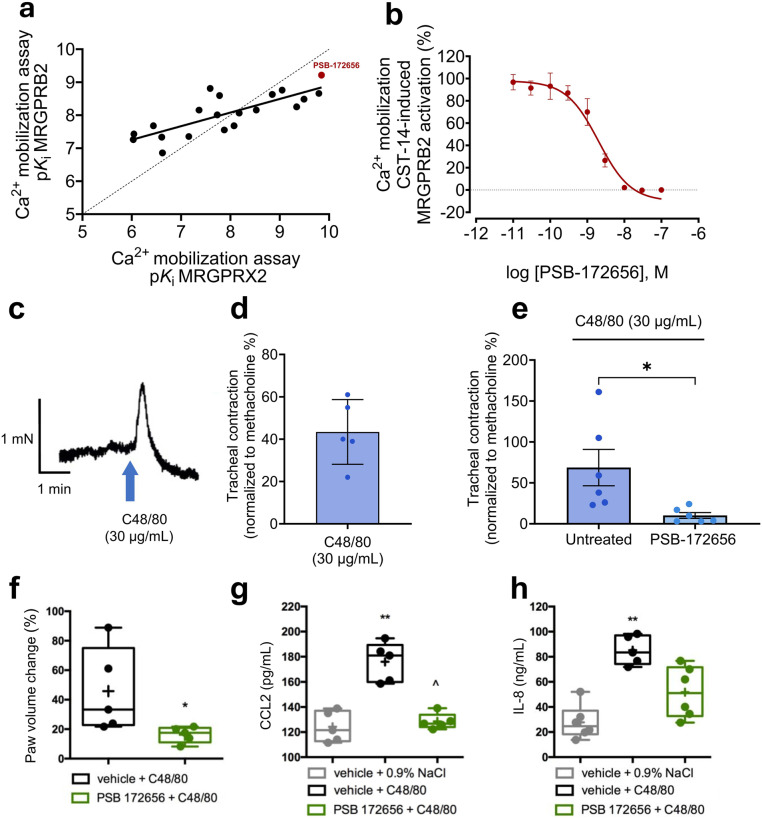
Evaluation of the effects of MRGPRX2 antagonists on the mouse ortholog MRGPRB2 **a** Correlation plot of p*K*_*i*_ values determined in Ca^2+^ assays for MRGPRX2 and MRGPRB2 antagonists (*r*^2^ = 0.791), the most potent antagonist PSB-172656 is shown in red. For MRGPRB2, the p*K*_*i*_ values of the compounds were determined using 1321N1 astrocytoma cells recombinantly expressing MRGPRB2 using Ca^2+^ assays versus the MRGPRX2/MRGPRB2 agonist CST-14 (EC_80_ value, 2000 nM). For MRGPRX2, p*K*_*i*_ values were determined using Ca^2+^ assays performed in LN229 cells recombinantly overexpressing MRGPRX2 versus the agonist CST-14 (EC_80_ value, 800 nM). **b** Concentration-response curve of PSB-172656 in Ca^2+^ assays using 1321N1 astrocytoma cells expressing MRGPRB2 versus CST-14 (EC_80_ value, 2000 nM), *K*_i_ value 0.302 ± 0.039 nM. Data are generated from 3 biological replicates, determined in duplicates. **c** A representative trace showing the effect of C48/80 (30 µg/mL) on a mouse tracheal ring in isometric force measurements using a wire-myograph system. **d** The contraction response of mouse trachea to C48/80 (30 µg/mL) was normalized to the contraction caused by methacholine (10 µM), a muscarinic receptors agonist. The data were generated from 5 biological replicates. **e** Contraction responses of the trachea to C48/80 (30 µg/mL) were assessed after treatment with PSB-172656 (100 µM) and compared to untreated controls (solvent,1% DMSO), the data were generated from 6 biological replicates. Statistical significance was determined by an unpaired Student’s *t*-test (*P*-value* < 0.05). **f** In vivo effects of PSB-172656 (50 mg/kg body weight (b.w.), *i.p*.) on local allergic reaction in mice (*n* = 5). **f** Paw edema was induced by injection of C48/80 (20 µL of a 30 µg/mL solution) into the right hind paw, and the percentage of volume increase compared to control was calculated; median (marked with a line) and mean (marked with “+”) ± SD (box) are shown. Mann–Whitney test (two-tailed); * - difference versus (vehicle + C48/80) was considered when (*P*-value* < 0.05). **g**, **h** Effect of PSB-172656 (50 mg/kg b.w., i.p.) on CC-chemokine ligand 2 (CCL2, **g**) and interleukin-8 (IL-8, **h**) levels in mouse plasma after C48/80-induced (i.p.) systemic anaphylaxis reaction. Median (marked with a line), mean (marked with “+”) ± SD (box), whiskers indicate minimum and maximum values; *n* = 5–6; Kruskal–Wallis test, Dunn’s post hoc; * difference versus (vehicle + 0.9% NaCl) was considered when *P* < 0.01 (**), ^ difference versus (vehicle + C48/80) was considered when *P* < 0.05 (^)

The mouse MRGPRB2 was suggested to be a functional ortholog of the primate MRGPRX2 due to similar expression patterns and partly intersecting pharmacology.^53^ Therefore, we investigated whether the new MRGPRX2 antagonists could also block MRGPRB2. To this end, we used recombinant 1321N1 astrocytoma cells that stably expressed MRGPRB2, employing Ca^2+^ assays, and CST-14 as MRGPRX2/MRGPRB2 agonist (EC_80_ value of 2000 nM).^31^ In fact, the compounds inhibited MRGPRB2 as well with p*K*_i_ values that showed a significant correlation with those determined for the human receptor (correlation coefficient (r) of 0.791) (Fig. 7a). Among them, PSB-172656 demonstrated the highest potency at both receptors; with a *K*_i_ value of 0.302 nM at MRGPRB2 (Fig. 7b) it was found to be only 3-fold less potent than at the human MRGPRX2.

In fact, both MRGPRX2 and MRGPRB2 share conserved residues predicted to be critical for the binding of PSB-172656, such as Glu and Asp residues (e.g., Glu164/Asp184 in MRGPRX2 and Glu171/Asp191 in MRGPRB2). These residues provide hydrogen bonding and electrostatic interactions crucial for stabilizing the antagonist (see Supplementary Figs. S7 and S8 for sequence alignment and comparison of the antagonist binding pockets of both species). The cysteine residues (Cys168/Cys180 in MRGPRX2 and Cys175/Cys187 in MRGPRB2) are well-positioned to interact with halogenated substituents, further contributing to the anchoring of the ligand. Additionally, hydrophobic residues (Phe239, Trp243 in MRGPRX2 and Phe246, Trp250 in MRGPRB2) in the binding pocket possibly contribute to ligand recognition and stabilization of the small-molecule antagonists. The binding pocket of MRGPRB2 for PSB-172656 shows subtle structural differences to that of MRGPRX2, which may explain the moderate differences in potency.

The MRGPRX2/MRGPRB2 agonist C48/80 was previously reported to induce tracheal contractions in WT mice, a response that was absent in mice with a frameshift mutation in *Mrgprb2*.^53^ We investigated whether this effect could be blocked by PSB-172656, the potent MRGPRX2/MRGPRB2 antagonist. Therefore, the force of tracheal contractions induced by C48/80 (30 µg/mL) in mouse tracheal rings was measured using a wire myograph, confirming the compound’s ability to provoke tracheal contractions (Fig. 7c, d). Pre-treatment with the antagonist PSB-172656 effectively blocked the contractions (Fig. 7e).

PSB-172656 was further investigated in a mouse model of skin inflammation,^54^ determining its effects on C48/80-induced hind paw edema. Systemic injection (*i.p*.) of the antagonist PSB-172656 followed by local injection of the agonist C48/80 into the right hind paw of the mice led to a significant decrease in paw swelling compared to controls without the antagonist (Fig. 7f). Additionally, we measured plasma levels of CC-chemokine ligand 2 (CCL2, Fig. 7g) and interleukin-8 (IL-8, Fig. 7h) as key markers of systemic anaphylaxis, both of which were significantly elevated following C48/80 treatment. Administration of PSB-172656 resulted in significantly decreased levels of CCL2. These findings indicate a strong anti-inflammatory effect of the MRGPRX2/B2 antagonist in this mouse model.

## Discussion

The recent discovery that the orphan GPCR MRGPRX2 likely plays a crucial role in mast cell activation has opened up new avenues.^19,53^ Specifically, MRGPRX2 may be key for modulating and eventually treating non-IgE-dependent hypersensitivities.^19^ These include urticaria,^23^ rosacea,^29^ and atopic dermatitis,^36^ but also allergic reactions against many drugs, all of which lack efficient treatment options.^19^ Moreover, various inflammatory diseases, including chronic inflammation and fibrosis, e.g., asthma and lung fibrosis,^31^ may benefit from treatment with MRGPRX2 antagonists. Although numerous structurally diverse MRGPRX2 agonists have been discovered,^19^ only a few antagonists have been described to date.^19^ Importantly, no optimization of hit compounds has been reported aimed at improving potency, selectivity, and drug-like properties. Neither structures of clinical candidates nor SARs have been disclosed. The compounds described in the patents all display strikingly similar peptidomimetic-like structures with relatively high MWs. The MRGPRX2 antagonist EP262 (INCB000262, structure undisclosed) was evaluated in a phase 2 clinical trial for the treatment of chronic urticaria, but the trial was recently halted because of preclinical in vivo toxicology findings (Incyte.com). One may speculate that the observed toxicity is due to the structure of the compound, not due to the target (MRGPRX2). Therefore, the antagonists described in the present manuscript, which are structurally very different, may not share EP262’s chronic toxicity.

In the present study, we set out to discover and develop qualified tool compounds with drug-like properties for blocking MRGPRX2. To this end, we selected an in-house compound collection of fragments (MW < 250 g/mol) and drug-like molecules (MW < 500 g/mol) and screened a subset of 4000 compounds. As an initial screening system, we used a *β*-arrestin recruitment assay based on *β*-galactosidase enzyme complementation, which had previously been employed for the screening of several orphan GPCRs,^55,56^ including MRGPRX4,^44^ with great success. *β*-Arrestin CHO cells recombinantly expressing MRGPRX2 were employed. In fact, we identified the drug-like, tractable, heterotricyclic hit compound **1**, which qualifies as a fragment due to its low MW of 241 g/mol. This allowed rational optimization by an integrated approach involving synthetic organic chemistry, molecular pharmacology, and computational studies.

Initial hit compound characterization confirmed MRGPRX2-blocking activity in an orthogonal assay measuring inhibition of G_q_ protein-mediated calcium mobilization in a different cell line, an MRGPRX2-overexpressing glioblastoma cell line (LN229). This cell line was established since it had been found to express MRGPRX receptors, including a low level of MRGPRX2, and was therefore expected to have MRGPRX2 signaling in place. Since hit compound **1** was additionally found to be specific for MRGPRX2, not blocking any other of the MRGPRX receptor subtypes (Fig. 1c), its structure was subsequently modified, and SARs were analyzed, with the aim of improving the antagonist’s potency.

Only a few optimization cycles were required to reach subnanomolar potencies (compounds **10,** **24** (PSB-172656), **28,** **29**, and **30**, see Fig. 2a). SARs were steep, indicating specific interactions of the different functional groups. According to our knowledge, these are the most potent MRGPRX2 antagonists reported to date, and they possess a unique chemical structure.

The SARs indicate a highly lipophilic binding site and an essential hydrogen bond, *N*1-H of the pyridone-containing scaffold acting as a hydrogen bond donor (see Fig. 2b) that likely interacts with the carboxylate function of Asp184 acting as an H-bond acceptor (see Fig. 4).

The most potent antagonists appeared to be more potent in inhibiting agonist-induced Ca^2+^ mobilization (G_q_-dependent signaling) than *β*-arrestin recruitment (Fig. 2c). However, this can be explained by their high percentage of binding to plasma proteins (Supplementary Table S3), present only in the *β*-arrestin, but not in the Ca^2+^ mobilization assay setup. In vivo, drugs showing high plasma protein binding may be protected from metabolic degradation.^57^ Moreover, it may result in sustained release of the drug, both effects potentially resulting in a long duration of action.^58^

Metabolic stability studies in human liver microsomes in vitro indeed showed good stability of the most potent antagonist PSB-172656, with an estimated half-life of >90 min (Supplementary Table S3). The di-fluoro-substitution, preventing hydroxylation of the phenyl ring by cytochrome P450 enzymes, appears to be important for metabolic stability since the corresponding dimethyl-analog **27** and the mono-fluoro derivative **30** were found to be metabolically less stable, exhibiting half-lives of 10.6 min (**27**), and 14.0 min (**30**).

Further, a broad characterization of PSB-172656 showed that it is non-cytotoxic, and highly selective versus other MRGPRX subtypes. Moreover, our studies indicated a competitive mechanism of action in blocking a range of structurally diverse agonists, including peptides like CST-14 and SP, the polymer C48/80, and small-molecule agonists such as (*R*)-ZINC-3573 and clomipramine.

Finally, the effects of the potent antagonist were evaluated at MRGPRX2 expressed in MCs. PSB-172656 potently blocks *β*-hexosaminidase release in rat RBL-2H3 cells recombinantly expressing human MRGPRX2, whereas IgE-mediated release is not affected. This inhibition was observed for MC degranulation induced by various agonists (Fig. 5). Thus, our data show that PSB-172656 appears to have a specific mechanism of action, not interfering with antigen-dependent signaling. Also, in the human LAD2 mast cell line, PSB-172656 blocks *β*-hexosaminidase release with similarly high potency (Fig. 6a). Since RBL-2H3 and LAD2 cells only represent MC models, which may differ from native human MCs, we additionally investigated human skin MCs from 3 donors. Again, PSB-172656 shows a concentration-dependent inhibition of *β*-hexosaminidase release induced by the MRGPRX2 agonist CST-14. The IC_50_ value for PSB-172656 in human skin MCs (ex vivo) appears to be in the nanomolar range since the lowest tested concentration (1.25 µM) already showed an inhibition of greater than 50%.

PSB-172656 selectively inhibits MRGPRX2-induced MC degranulation without affecting IgE or C3a pathways, emphasizing its selectivity rather than general inhibition of mast cell activation.^59^ This highlights its potential as a therapeutic agent with minimal off-target effects. Additionally, PSB-172656 may be employed as a diagnostic tool for predicting MRGPRX2-mediated drug hypersensitivities, distinguishing them from the typical IgE- and C3a-dependent allergies.

PSB-172656 cannot only be used to study the human MRGPRX2 in suitable systems, e.g., in humanized mice expressing the human receptor,^60^ but the antagonist also block its proposed mouse ortholog, MRGPRB2, with similarly high, subnanomolar potency (Fig. 7). In fact, the proposed binding sites of both receptors, MRGPRX2 and -B2, for the small antagonist PSB-172656 are very similar (see Fig. S8) harboring several crucial amino acid residues required for the strong interaction. Since no antagonist-bound structures have been published, docking of the competitive antagonist was performed based on the agonist-bound structures and associated mutagenesis data^32^, providing a highly plausible binding pose (Fig. 4). PSB-172656 is a sterically fixed molecule with little flexibility, which confers high reliability to the docking study. Molecules with peptidomimetic-like structures that are larger than PSB-172656, can be expected to show larger species differences since they require interaction with additional amino acid residues that may differ in human and mouse receptors (for sequence alignment, see Fig. S7). A functional ortholog of the human MRGPRX2 in dogs sharing 62% sequence homology was reported.^61^ The dog MRGPRX2 is also expressed in MCs and plays a role in drug-induced allergic reactions similar to the human receptor. Whether the new MRGPRX2/MRGPRB2 antagonists also block canine MRGPRX2 warrants further study considering the animals’ role in preclinical drug development and veterinary medicine.^61^ PSB-172656 emerges as a promising candidate for such studies.

Contractions of mouse trachea in response to the MRGPRX2/MRGPRB2 agonist C48/80 were previously reported.^53^ In the present study, we demonstrated that PSB-172565 potently blocks tracheal contractions induced by C48/80 via MRGPRB2 inhibition, suggesting that it might be useful for MRGPRX2/B2-induced allergic asthma; however, its value for treating airway inflammation still requires confirmation. Activation of MRGPRX2/B2 expressed on MCs, is able to quickly affect tissue homeostasis in inflammatory processes,^62^ leading, for example, to tissue swelling and an increase in tissue levels of pro-inflammatory chemokines. Our results demonstrated that PSB-172656, systemically applied, prevents or reduces edema formation in a mouse model of inflammation induced by local application of the MRGPRX2/B2 agonist C48/80 (mouse hind paw edema, Fig. 7f). Systemic application of C48/80 results in an anaphylactic reaction,^63^ characterized by an increase in CCL2 and IL-8 plasma levels.^45^ Again, pre-treatment with PSB-172656 prevented the dramatic increase in CCL2 levels (Fig. 7g), indicating that the antagonist may protect from MRGPRX2/B2-mediated anaphylaxis.

The main limitation of the current study is the fact that MRGPRB2 does not qualify as the direct, closely related, sole ortholog of the primate-specific MRGPRX2.^19^ Clinical studies, or at least studies in non-human primates, will therefore be indispensable to examine and confirm the in vivo effects of PSB-172656. The antagonist is only active against MRGPRX2/B2-induced MC degranulation, but there are other pathways that induce degranulation.^64^ The antagonist also requires further, more comprehensive characterization regarding chronic toxicity and with respect to its residence time.^65^ Also, a cryo-EM structure or a co-crystal structure of the antagonist-bound MRGPRX2 would be useful as a basis for further drug development efforts, and it would provide insights into the inactive conformation of the receptor, which is still lacking. Finally, MRGPRX2/B2-dependent contributions to systemic allergic reactions, impacts on different organs and tissues, and their blockade by PSB-172656 require further study.

Nevertheless, MRGPRX2/MRGPRB2 antagonists, specifically the highly potent compound PSB-172656, will serve as valuable tools for studying and understanding the roles of these receptors in various pathological conditions, including asthma,^24^ idiopathic lung fibrosis,^31^ allergic rhinitis,^34^ chronic urticaria,^35^ atopic dermatitis,^36^ and further allergic and inflammatory diseases.^19^ PSB-172656 is by far the most potent MRGPRX2 antagonist described to date, possessing a non-peptidomimetic-like scaffold, and it additionally blocks the receptor’s putative mouse ortholog MRGPRB2 with similarly high potency. The antagonist possesses drug-like properties and has shown efficacy in human MCs. Moreover, it was demonstrated to block the corresponding MRGPRB2 in vivo in native mouse models of anaphylaxis and inflammation.

In conclusion, PSB-172656 is expected to become an invaluable tool compound for studying MRGPRX2 and its mouse ortholog in vitro and in vivo and has the potential to be developed for the treatment of so far difficult-to-treat mast cell-dependent diseases.

## Materials and methods

### *β*-Arrestin recruitment assays

Eurofins DiscoverX (Fremont, CA, USA) provided engineered Chinese hamster ovary (CHO) cells stably expressing *β*-arrestin-2 fused to a large *β*-galactosidase fragment (enzyme acceptor, EA). These CHO-K1-*β*-arrestin-2 cells were commercially available (93-0164). CHO-K1-β-arrestin-2 cells that additionally expressed MRGPRX1, -2, or -4 fused to the fragment ProLink™ (PK) were also purchased from the same company (93-0919C2, 93-0309C2, and 3-0541C2A, respectively). CHO cells expressing MRGPRX3 were generated as previously decided.^31^ The cells were grown in F-12 Ham Nutrient Mixture (Life Technologies) supplemented with 10% FBS, 100 U/mL of penicillin, 100 µg/mL of streptomycin (PAN-Biotech), 300 μg/mL of hygromycin B (PAN-Biotech), and 800 μg/mL of gentamicin (PAN-Biotech) at 37 °C and 5% CO_2_.).

The assays were carried out as previously described.^22^ On the day before the assay, cells were seeded into 96-well plates (Nunclon Delta surface plates, Thermo Fisher Scientific) at a density of 2.5 × 10^5^ cells/mL per well in 89 μL of Opti-MEM medium (Thermo Fisher Scientific) supplemented with 2% FBS, 100 U/mL of penicillin, 100 µg/mL of streptomycin, 800 μg/mL of gentamicin, and 300 μg/mL of hygromycin B. The antagonists were diluted in DMSO and added to the cells; the final DMSO concentration did not exceed 1% (v/v). After 30 min of incubation at 37 °C and 5% CO_2_, Cortistatin-14 (CST-14, Bioscience, Bristol, United Kingdom) or another MRGPRX2 agonist that served as a standard agonist was added, and the mixture was incubated for 90 min at 37 °C and 5% CO_2_. Next, a detection reagent (50 µL, DiscoverX) was added, and the mixture was incubated at room temperature in the dark for 60 min. Chemiluminescence was measured on a Mithras LB 940 plate reader (Berthold Technologies, Bad Wildbad, Germany). The results represent means ± SEM of three to five independent experiments, each in duplicates.

### Calcium mobilization assays

Glioblastoma LN229-MRGPRX2 stably expressing MRGPRX2 (produced by retroviral transfection) were seeded into black sterile 96-well microplates at a density of 5 × 10^4^ cells/well in 200 µL of DMEM/F12-Nut Mix medium, supplemented with 10% FCS, 100 U/mL of penicillin 100 µg/mL of streptomycin, and 200 µg/mL of G418 and incubated for 18–22 h.

The next day Fluo-4 AM Solution (1 mM) (Thermo Fisher Scientific) was prepared and used to prepare cell loading dye solution consisting of 4970 µL of Hank’s Buffered Saline Solution (HBSS) buffer, 15 µL of 20% Pluronic-F and 15 µL of 1 mM Fluo-4 AM. Next, the cell medium was removed and exchanged for 40 µL of dye solution per well (final concentration of Fluo-4 AM: 3 µM). The cell plate was incubated for 60 min in the dark at room temperature, then the dye solution was removed and replaced by 189 µL HBSS. Subsequently, 1 µL of antagonist diluted in DMSO was added and incubated for 30 min at room temperature and in the dark (final DMSO concentration 0.5%, v/v). CST-14 or another agonist prepared in HBSS as well as carbachol (final concentration 100 µM) were pipetted (10 µL) using a FlexStation^®^ 3 Multi-Mode Microplate Reader. For the determination of baseline fluorescence, HBSS buffer containing solvent of the investigated compound was used. Fluorescence was measured for 120 intervals of 1 s (number of flashes: 10) at a wavelength of 525 nM (excitation 485 nM) using the SoftMax^®^ Pro5.1 Microplate Data Acquisition & Analysis Software. Three independent experiments were performed in duplicates. Measurements are provided in relative fluorescence units and normalized to the maximal effect of CST-14.

### MTT assay

For the experiment, 1321N1 astrocytoma cells were seeded at a density of 1 × 10^5^ cells/well in 180 µL of cell medium into transparent 96-well plates, whereby the outer wells only contained buffer (to avoid an edge effect). Cells were incubated for 24 h at 37°C, 5% CO_2_. Compound dilutions were prepared in DMSO. The DMSO concentration in the assay did not exceed 1%. Subsequently, 20 µL of these compound dilutions were added to each well so that astrocytoma cells were incubated with varying concentrations of MRGPRX2 antagonists (0.01 µM–10 µM) at 37° C, 5% CO_2_. 5-Fluoruracil, a cytostatic drug, served as a positive control. Medium-containing DMSO in the absence of a compound served as a negative control. After 71 h, 40 µL of MTT solution (5 mg/mL, prepared in Millipore water) was added to each well (final concentration 0.5 mg/mL) and cells were incubated again for another hour. Subsequently, the medium was removed, and cells were lyzed by adding 100 µL of DMSO per well. In order to dissolve the produced formazan dye crystals, plates were incubated for 2 min on a rotary shaker (150 rpm). Afterward, the UV absorbance at 570 nm was measured by using SoftMax^®^ Pro5.1 Microplate Data Acquisition & Analysis Software (Molecular Devices, Sunnyvale, CA, USA). The UV absorbance at 690 nm was measured directly afterward to eliminate the background absorbance of residual medium and cell components. The values of the measured UV absorbance at 690 nm were subtracted from the values obtained by the measurement of UV absorbance at 570 nm. Three to four independent experiments were performed, each in triplicate.

LAD2 cells (5 × 10^3^ cells/well in a 96-well plate) were exposed to compound PSB-17-2656 (3, 10, 30 nM) or vehicle (PBS). After 24 h, MTT solution was added to each well, and incubated for an additional 4 h. Supernatants were removed, and DMSO (100 µL, Sigma, USA; Cat #D2660-100ML) was added. The plate was shaken to dissolve the formazan, and absorbance was measured at 570 nm using a Versamax microplate spectrophotometer (Molecular Device, San Jose, CA, USA). Cell viability was measured according to the above-mentioned procedure.

### Degranulation assay determined by *β*-hexosaminidase release

Rat basophilic leukemia (RBL-2H3) cells that stably expressed the human MGPRGX2 (RBL-MRGPRX2) were grown in Dulbecco’s modified Eagle’s medium (DMEM) supplemented with 10% FBS, l-glutamine (2 mM), penicillin (100 IU/mL), and streptomycin (100 µg/mL).^42^

Degranulation as determined by *β*-hexosaminidase release was performed as described previously.^66,67^ Briefly, RBL-MRGPRX2 cells (5 × 10^4^ cells/well) were seeded into 96-well cell culture plates. After 16 h, cells were treated with PSB-172656 or solvent for 5 min in HEPES buffer containing 0.1% bovine serum albumin (BSA), and then activated with MRGPRX2 agonist (C48/80, SP, PAMP-12, or LL-37) for 30 min at 37 °C. For measurement of total *β*-hexosaminidase content, unstimulated cells were lyzed by Triton X-100 (0.1%). Aliquots (20 µL) of supernatant were incubated with 20 µL of 1 mM 4-nitrophenyl-*N*-acetyl-β-D-glucosaminide (PNAG) for 1 h at 37°C. Finally, the reaction was stopped by adding 250 µL of 0.1 M Na_2_CO_3_/0.1 M NaHCO_3_ buffer, and absorbance was determined with a Versamax microplate spectrophotometer (Molecular Devices, San José, CA) at a wavelength of 405 nm. The degranulation was quantitated as a percentage of *β*-hexosaminidase release by dividing the *β*-hexosaminidase in the sample by the total enzyme content. To test the antigen response, RBL-MRGPRX2 (5 × 10^4^ cells/well) cells were sensitized with DNP-specific mouse IgE (SPE-7; 1 µg/mL). After 16 h, cells were preincubated in the absence or presence of respective concentrations of PSB-172656 for 5 min, followed by the stimulation of antigen (DNP-BSA, 10 and 30 ng/mL), and the degranulation was measured as described above. For degranulation in LAD2 cells, 1 × 10^4^ cells/well were seeded into 96-well cell culture plates, incubated with or without PSB-172656 for 5 min, then with the MRGPRX2 agonist SP for 30 min, and degranulation was measured similarly.

Cell culture reagents, 2,4-dinitrophenylated bovine serum albumin (DNP-BSA; Cat # A23018), and DNP-specific mouse IgE (SPE-7) were obtained from Invitrogen (Gaithersburg, MD, USA).

### Degranulation measured by the surface expression of CD107a and CD63

The human mast cell line Laboratory of Allergic Diseases 2 (LAD2) was kindly provided by Dr. A. Kirshenbaum and Dr. D. Metcalfe (Laboratory of Allergic Diseases, National Institute of Allergy and Infectious Diseases, National Institute of Health, Bethesda, MD, USA) and maintained in a complete StemPro-34 medium supplemented with L-glutamine (2 mM), penicillin (100 IU/mL), streptomycin (100 µg/mL), and recombinant human stem cell factor (rhSCF, 100 ng/mL). rhSCF was purchased from PeproTech (Rocky Hill, NJ, USA).

MC degranulation was also measured by determining the cell surface expression of CD107a (Lysosomal-Associated Membrane Protein-1, LAMP-1) and CD63 (LAMP-3) after agonist stimulation.^48^ LAD2 cells were preincubated with PSB-172656 or vehicle control for 5 min at 37 °C, then activated with SP (0.3 μM) and or C3a (3 nM) for 5 min at 37 °C. After 5 min of incubation, cells were fixed with 500 µL of 4% fixation buffer (Biolegend, Catalog 420801) for 15 min at room temperature. The reaction was stopped with cold FACS buffer [1X DPBS containing 2% fetal calf serum (FCS) and 0.02% sodium azide] and blocked with 1% BSA in 1X DPBS for 30 min at 4 °C to reduce non-specific binding. Cells were incubated with zombie-yellow (Live/Dead staining; BioLegend, cat #77168) and fluorescein isothiocyanate-conjugated anti-human CD107a and allophycocyanin-conjugated anti-human CD63 antibodies for 30 min at 4 °C. Cell surface expression of CD107a and CD63 was measured from the zombie-yellow negative population by a BD LSR II flow cytometer (San Jose, CA, USA) and analyzed by FlowJo software version 10.7.2 (Tree Star Inc., Ashland, OR, USA).^48,50^

Flow cytometry antibodies, allophycocyanin-conjugated anti-human CD63 (Clone H5C6, Catalog 353008), and fluorescein isothiocyanate-conjugated anti-human CD107a (Clone H4A3, Catalog 328606) were from BioLegend (San Diego, CA, USA).

### Degranulation of human skin mast cells

MCs were isolated from breast skin tissue, which was sectioned into strips and digested overnight at 4 °C in 2.4 U/mL dispase type II (Fisher Scientific). Following this, the epidermis was removed, and the dermal tissue was minced into small fragments and further digested for 1 h at 37 °C in PBS containing Ca^2+^ and Mg^2+^ (Gibco, Carlsbad, CA, USA) with 1% penicillin-streptomycin (Pen/Strep), 5% fetal calf serum (FCS), 2.5 μg/mL amphotericin B (Thermo Fisher), 5 mM MgSO_4_, 10 μg/mL DNase I (Omnilab), 0.75 mg/mL hyaluronidase (Sigma-Aldrich), and 1.5 mg/mL collagenase (Worthington Biochemical), under constant agitation. The resulting cell suspension was filtered sequentially through 300 μm and 40 μm sieves (Retsch), followed by centrifugation at 300 × *g* for 15 min at 4 °C. The digestion process was repeated once. After digestion, the cells were washed in PBS (Gibco) not containing Ca^2+^ or Mg^2+^. MCs were then isolated via CD117-positive magnetic-activated cell sorting (MACS) enrichment (Miltenyi) and cultured in basal Iscove’s medium supplemented with 1% Pen/Strep, 10% FCS, 1% non-essential amino acids (all Gibco), and 226 μM α-monothioglycerol. The purity of the MC cultures was assessed by flow cytometry for CD117 and FcεRI (Miltenyi) and exceeded 95%. Recombinant human interleukin-4 (IL-4, 20 ng/mL; Peprotech) and human stem cell factor (SCF, 100 ng/mL; Miltenyi) were added 24 h post-isolation. Cultures were maintained at a density of 1.0 × 10^6^ cells/mL, with IL-4 and SCF freshly added twice a week.

For flow cytometry analysis of human skin MCs, freshly isolated human skin MCs were gently washed 2 times with PBS/BSA/EDTA and subsequently incubated with 30 µL of the staining cocktail containing anti-human FcεRIα [AER-37 (CRA-1)] Brilliant Violet 605™ (BioLegend), anti-human MRGPRX2 (K125H4) APC (BioLegend), and anti-human CD117 PE-Vio® 615, REAfinity™ (Miltenyi) and incubated for 15 min at 4°C. After incubation, the cells were centrifuged, washed, and resuspended in 100 µL of PBS/BSA/EDTA prior to measurement. The cells were analyzed on a SONY ID7000 spectral cell analyzer (Sony Biotechnology) and further analyzed by FlowJo software (FlowJo version 10.8.0; BD).

For *β*-hexosaminidase release assays, MCs were fed the day before stimulation with the medium. The next day, the cells (2 × 10^5^) were seeded into a 96-well plate in a total volume of 50 μl of HEPES-Tyrode’s buffer. For the experiments with MRGPRX2 antagonist, the cells were incubated for 30 minutes with warm Tyrode’s containing the respective antagonist in final concentrations of 0.5–10 µM. Subsequently, the cells were incubated for 1 h with CST-14 (1 µM), Tyrode’s buffer, or Ionomycin (1 µM) at 37 °C. After stimulation, the cells were centrifuged, and 50 µL of supernatants were collected. The cells were lyzed in 100 µL of distilled water, and lysates were rapidly frozen at −80 °C. After thawing, 50 µL of lysates and supernatants were incubated for 1 h at 37 °C with the same amount of 4-methylumbelliferyl *N*-acetyl-*β*-D-glucosaminide (Sigma-Aldrich, Munich, Germany) diluted in citrate buffer (pH 4.5) to measure the level of secreted and intracellular hexosaminidase. The reaction was stopped by adding 100 µL of sodium carbonate buffer (pH 10.7), and fluorescence was measured at 460 nm and excitation at 355 nm for 0.1 s. The percentage of *β*-hexosaminidase release was calculated as: (optical density [OD] of lysates + OD of supernatants)/OD of supernatants × 100. Then, the effect of CST-14 in the presence of PSB-172656 was corrected to the effect of CST-14 incubated with the vehicle only.

### Isometric force measurements of mouse tracheal rings on wire-myograph

Isometric force measurements were performed as described previously.^68^ In detail, tracheas of C57BL/6 mice were isolated and cut into rings in cold low-calcium physiological saline solution (PSS), containing 118 mM NaCl, 5 mM KCl, 1.2 mM MgCl2, 1.5 mM NaH_2_PO_4_, 0.16 mM CaCl_2_, 10 mM glucose and 24 mM Hepes (pH 7.4). Rings were mounted on a wire-myograph (Multi Myograph 620 M, Danish Myo Technology, Aarhus, Denmark) and pre-stretched to 5 mN in PSS, containing 118 mM NaCl, 5 mM KCl, 1.2 mM MgCl_2_, 1.5 mM NaH_2_PO_4_, 1.6 mM CaCl_2_, 10 mM glucose, and 24 mM Hepes (pH 7.4). After equilibration for 20 min, maximal constriction was induced by methacholine (MCh, 10 μM, A2251, Sigma-Aldrich, Germany). After washout, C48/80 (30 µg/mL, Sigma-Aldrich, Germany) was applied. In some experiments, PSB-172656 (100 µM) or the solvent (1% DMSO) was incubated for 10 min before addition of C48/80. Force amplitudes were always analyzed at the strongest contraction of tracheal rings. For analysis, LabChart 8 (ADInstruments, Oxford, UK) was used. Statistical significance was determined by an unpaired Student’s *t*-test, *P < 0.05.

### In vivo studies

#### Animals

Mice were obtained from the Jagiellonian University Animal House at the Faculty of Pharmacy in Krakow, Poland. Fifty male C57BL/6 mice (22–27 g) were housed per standard cage while maintaining a 12:12-h light/dark cycle, with food and water available ad libitum. The experimental procedures were performed from 9:00 a.m. to 5:00 p.m. All experiments involving equal treatment of animals were conducted by experimenters blind to the experimental groups.

#### Local allergic reaction (hind paw edema)

Mice were given the antagonist PSB-172656 intraperitoneally at a dose of 50 mg/kg b.w. or vehicle (2% DMSO). The volume of the paws (both right (test) and left (control)) was measured using a plethysmometer (7140, UgoBasile, Italy). After 30 min, 20 µL of C48/80 (30 µg/mL) were injected into the right paw and 20 µL of 0.9% NaCl into the left paw. After 15 min, paw thickness was measured again. Mice were then killed by dislocation of cervical vertebrae. The mouse model has been described previously.^54,69^

#### Systemic anaphylactic reaction

30 min after intraperitoneal administration of PSB-172656 at a dose of 50 mg/kg b.w. or vehicle (2% DMSO), an anaphylactic reaction was induced by the intraperitoneal injection of C48/80 at a dose of 2 mg/kg b.w. (in 0.9% NaCl). The non-lethal dose of this compound was assumed based on the literature.^70,71^ The mice were then left in their cages for 4 h. Then, heparin (2500 U/mouse) and sodium thiopental at a dose of 70 mg/kg b.w. were administered intraperitoneally, and blood was collected after decapitation. The plasma concentration of CCL-2 and IL-8 was determined using an ELISA kit (catalog number: E1707Mo, E1481Mo, BT LAB, China).

### Statistical analysis

GraphPad PRISM software (version 9.0.1; San Diego, CSA, USA) was used to determine the statistical values. For concentration-response curves, sigmoidal response parameters (variable slope) were used to determine the potencies of the compounds. The obtained IC_50_ values were converted to *K*_i_ values using the Cheng–Prusoff equation to calculate the affinity of the ligands.^72^

Correlation between groups was analyzed by Pearson correlation and two-tailed *P*-value < 0.001 was considered to be significant. Differences between groups were analyzed by analysis of variance (ANOVA) followed by Tukey’s multiple comparison tests. *P*-value ≤ 0.05 was significant. Statistical significance was determined by one-way or two-way ANOVA test (numerical *P* valued are indicated unless *P* < 0.001 or in some cases <0.0001). Additionally, statistical significance between two groups was determined using an unpaired Student’s *t*-test, (*P*-value* < 0.05). For animal studies, non-parametric tests were used (Mann–Whitney test or Kruskal–Wallis’s test with Dunn’s post hoc). The data are displayed as the mean ± standard deviation (SD) and median.

## Supplementary information

Supplementary Information

Supplementary Data S1

Supplementary Data S2

## Data Availability

The data produced or assessed in the present study are included in the published article and the associated supplementary data. Further information and raw data are available from the corresponding author upon reasonable request. All plasmids were deposited with Addgene: pLXSN-MRGPRX2 (Plasmid #213960) and pLVX-IRES-MRGPRB2-mCherry (Plasmid #213961).
