# Supplementary material for: Subnanomolar MAS-related G protein-coupled receptor-X2/B2 antagonists with efficacy in human mast cells and disease models
Source: Signal Transduct Target Ther. 2025 Apr 21;10:128. doi: 10.1038/s41392-025-02209-8 (PMC12010006; doi:10.1038/s41392-025-02209-8)
Supplement: Supplementary file 1 — Supplementary Information [file 41392_2025_2209_MOESM1_ESM.docx]

Supplementary Materials for

**Subnanomolar MAS-related G protein-coupled receptor-X2/B2 (MRGPRX2/B2) antagonists with efficacy in human mast cells and disease models**

Ghazl Al Hamwi, Mohamad Wessam Alnouri, Sven Verdonck, Piotr Leonczak, Shaswati Chaki, Stefan Frischbutter, Pavel Kolkhir, Michaela Matthey, Constantin Kopp, Marek Bednarski, Yvonne K. Riedel, Daniel Marx, Sophie Clemens, Vigneshwaran Namasivayam, Susanne Gattner, Dominik Thimm, Katharina Sylvester, Katharina Wolf, Andreas E. Kremer, Steven De Jonghe, Daniela Wenzel, Magdalena Kotánska, Hydar Ali, Piet Herdewijn, Christa E. Müller

Correspondence to: christa.mueller@uni-bonn.de

**This PDF file includes:**

Figures. S1 to S7

Tables S1 to S3

Captions for Data S1 and S2

**Other Supplementary Materials for this manuscript include the following:**

Data S1 and S2

Figure. S1.

Concentration-response curve of standard MRGPRX2 agonist: Concentration-response curve of CST-14 determined in *β*-arrestin-CHO cells recombinantly expressing MRGPRX2; EC_50_ 483 ± 85 nM. Data are means ± SEM from 4 biological replicates, in duplicates.


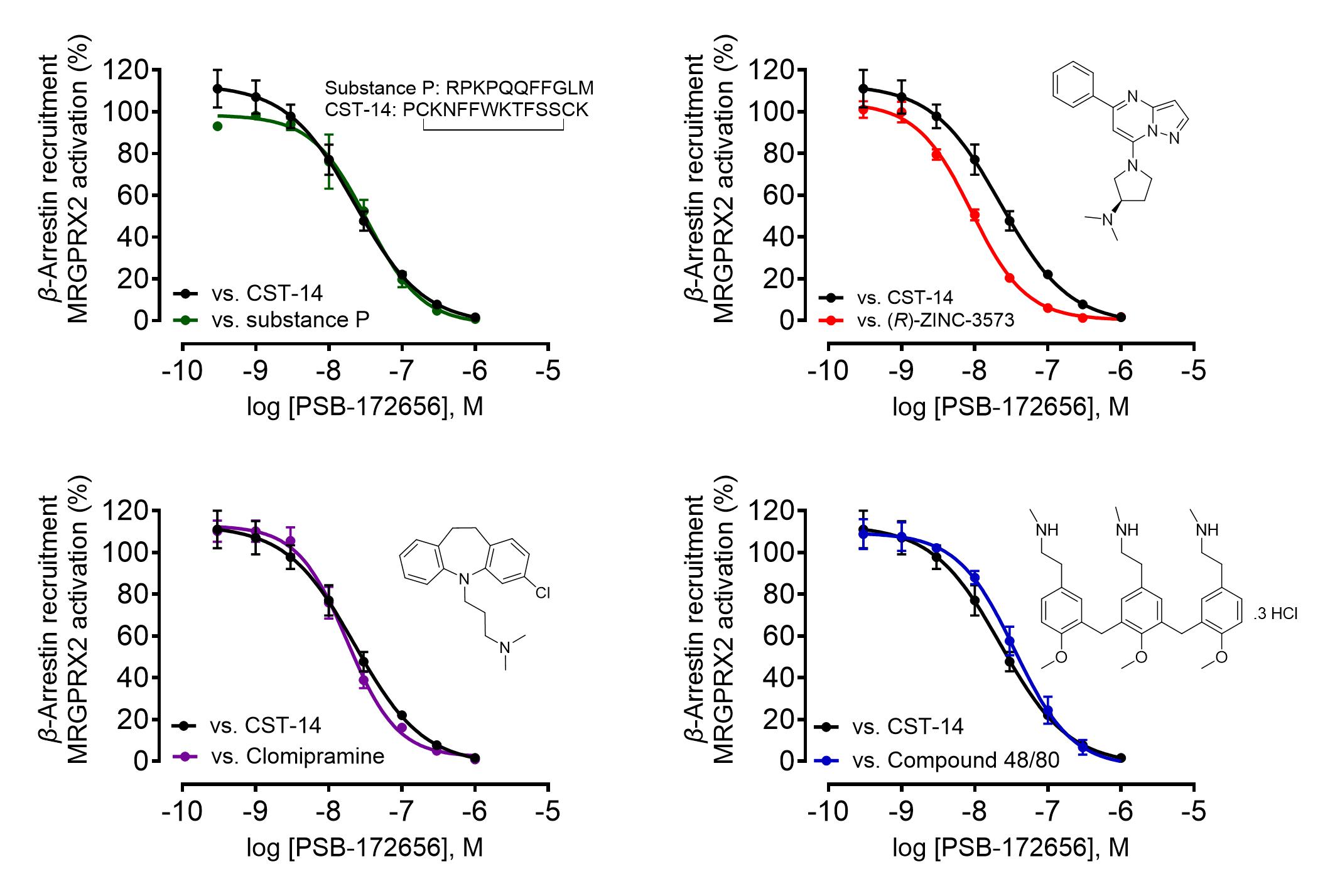


**c d**

**a b**

Figure. S2.

PSB-172656 inhibits structurally diverse MRGPRX2 agonists: Concentration response curves of PSB-172656 using *β*-arrestin recruitment assays versus structurally diverse MRGPRX2 agonists, (**a**) versus the peptide agonist substance P (EC_80_ 3 µM) displaying an IC_50_ value of 30.5 ± 6.3 nM, *K*_i_ value of 8.82 ± 3.64 nM; (**b**) versus the small molecule agonist (*R*)-ZINC-3573 (EC_80_ 2 µM) resulting in an IC_50_ value of 11.3 ± 3.1 µM nM, *K*_i_ value of 5.44 ± 2.98 nM; (**c**) versus the tricyclic antidepressant and MRGPRX2 agonist clomipramine (EC_80_ 13 µM) showing an IC_50_ value of 17.2 ± 0.4 nM, *K*_i_ value of 7.04 ± 0.32 nM; (**d**) versus MRGPRX2 agonist compound 48/80 (EC_80_ 18 µM) showing an IC_50_ value of 39.5 ± 9.0 nM, *K*_i_ value of 3.68 ± 1.68 nM. Concentration response curves of PSB-172656 versus CST-14 (EC_80_ 1 µM) was used as control showing an IC_50_ value of 39.5 ± 9.0 nM, *K*_i_ value of 6.81 ± 3.51 nM. Data are means ± SEM of 3 biological replicates, performed in duplicates.


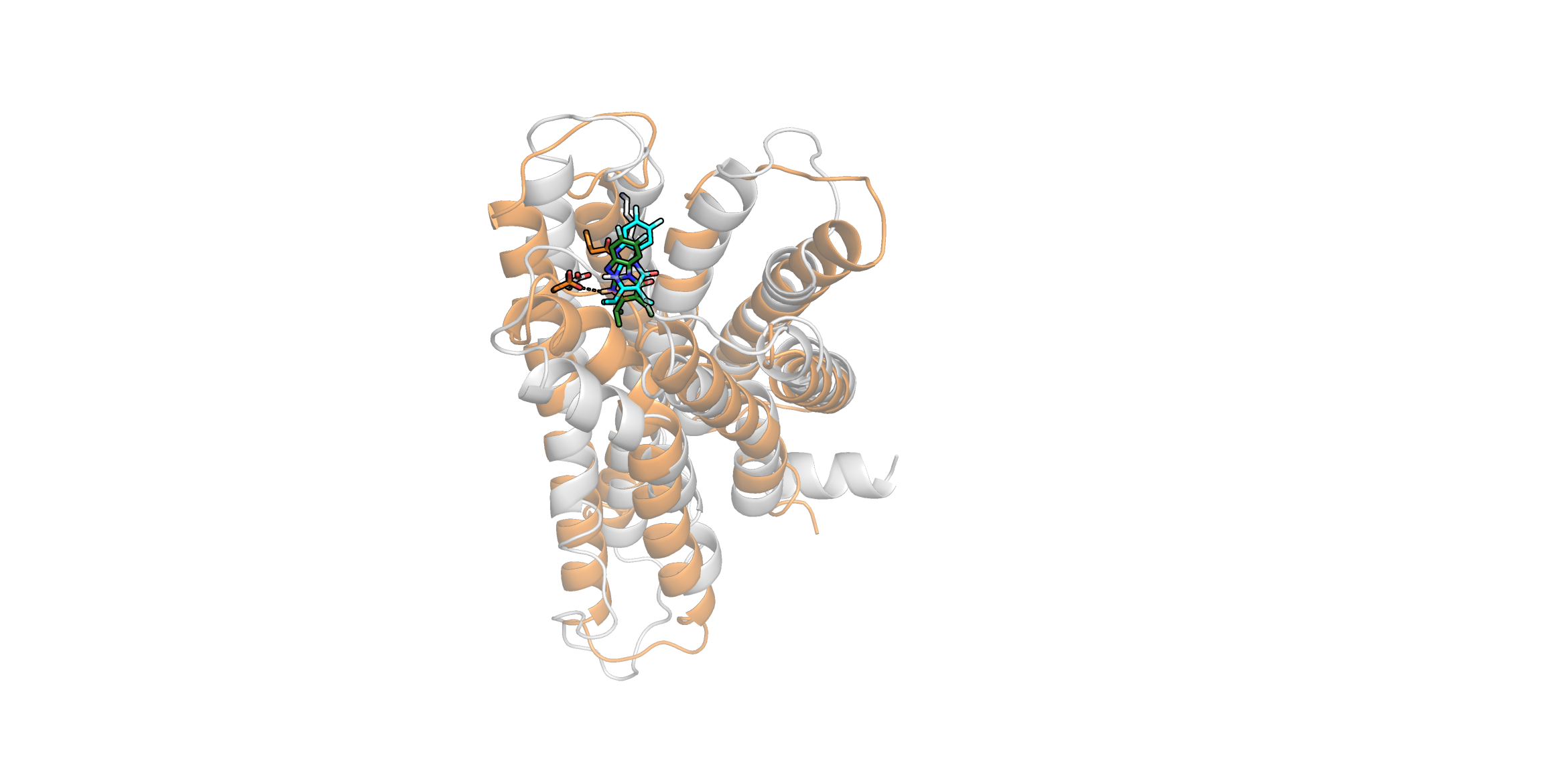


Figure. S3.

Overlapping docking poses of PSB-172656 in MRGPRX2 receptor structures: homology model and cryo-EM template. Docked pose of PSB-172656 (carbon colored green) in the putative orthosteric binding pocket of the human MRGPRX2 cryo-EM structure (orange, PDB: 7S8L). The human MRGPRX2 model is shown in cartoon representation. The putative binding pocket is located near the surface of MRGPRX2 (side-view). PSB-172656 (carbon colored cyan) docked into the binding pocket of the MRGPRX2 homology model (gray) showing large overlap with the docked pose into the cryo-EM structure. An overall Root-Mean-Square Deviation (RMSD) value of 2.77 was determined.


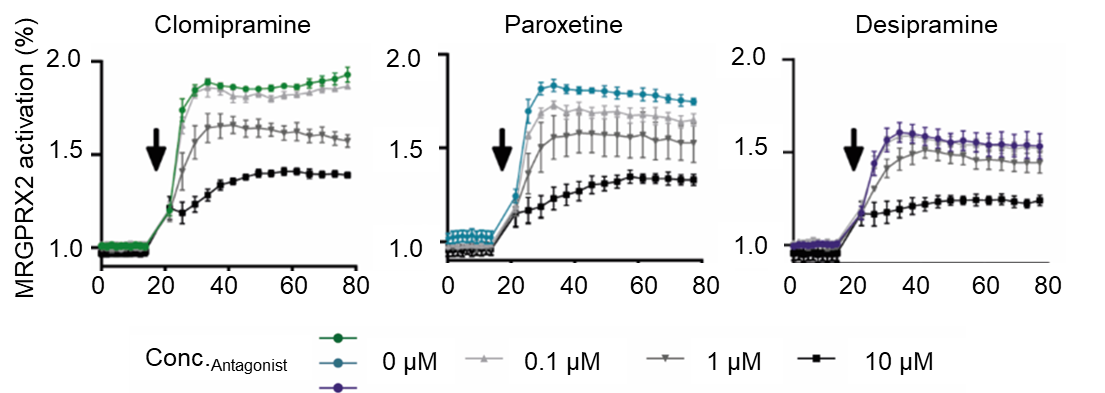


Figure. S4.

Blocking of MRGPRX2 by the selective antagonist PSB-172656. PSB-172656 decreased Ca^2+^ mobilization upon stimulation with clomipramine (16.0 µM), paroxetine (18.1 µM), and desipramine (34.4 µM) in LAD2 cells. Arrows indicate addition of stimulus; graphs represent means ± SEM of three independent experiments.


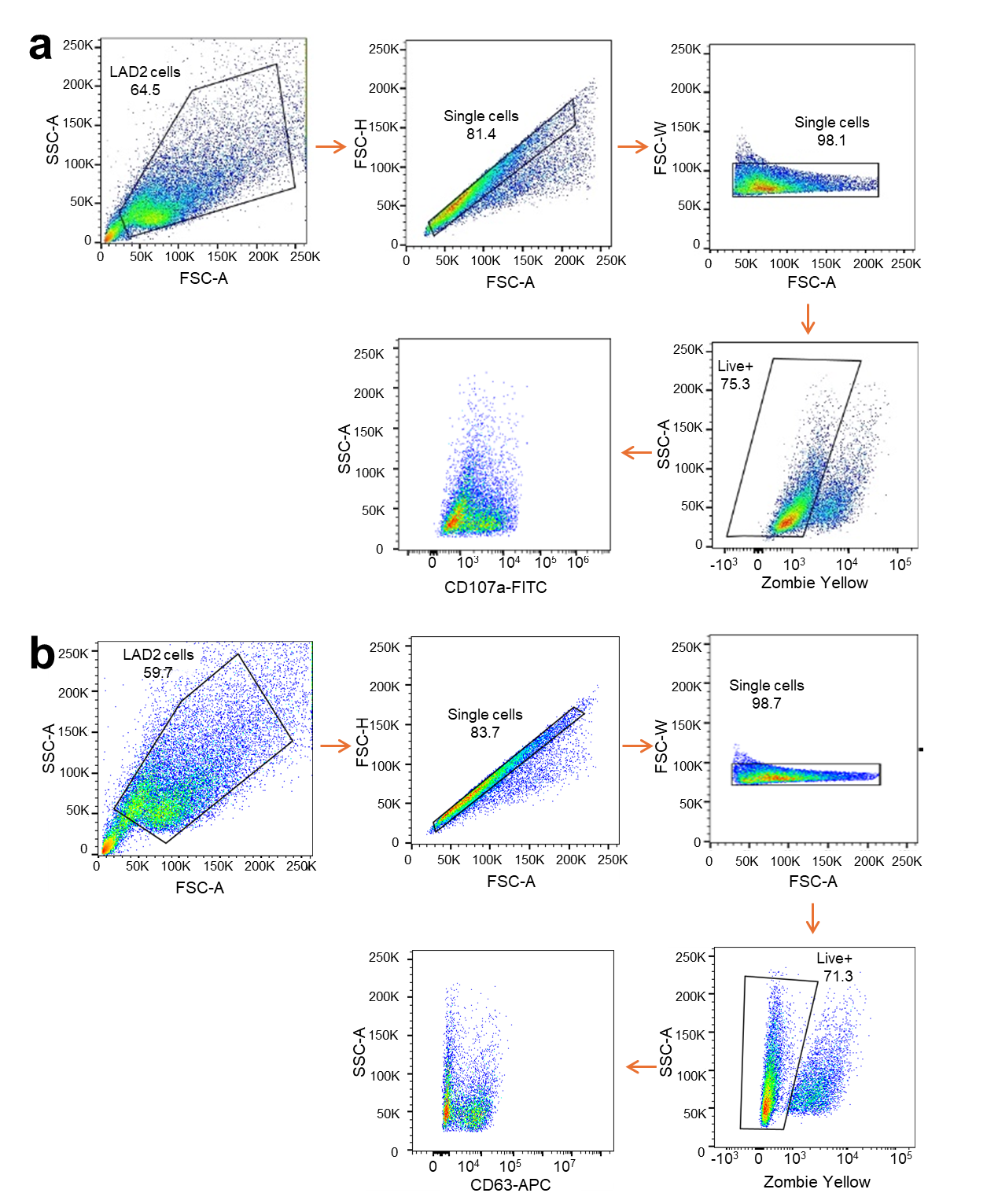


Figure. S5.

Gating strategy for flow cytometry analysis of LAD2 cells. LAD2 cells were gated according to size and granularity in the FSC/SSC plot, excluding debris. FSC-H versus FSC-A and FSC-W versus FSC-A plots were sequentially used to select and refine the single-cell population, ensuring the exclusion of doublets and aggregates. SSC-A versus Zombie Yellow™ staining gate was used to exclude dead cells and focus on the live cell population. LAD2 were gated as (**a**) Zombie-yellow^-^ CD107a^+^ live cells. (**b**) Zombie-yellow^-^ CD63^+^ live cells. FSC: Forward Scatter, SSC: Side Scatter, FSC-H: FCS-Height, FSC-A: FCS-Area, FSC-W: FCS-Width, SSC-A: SSC Area. The data are generated from 3 biological replicates.


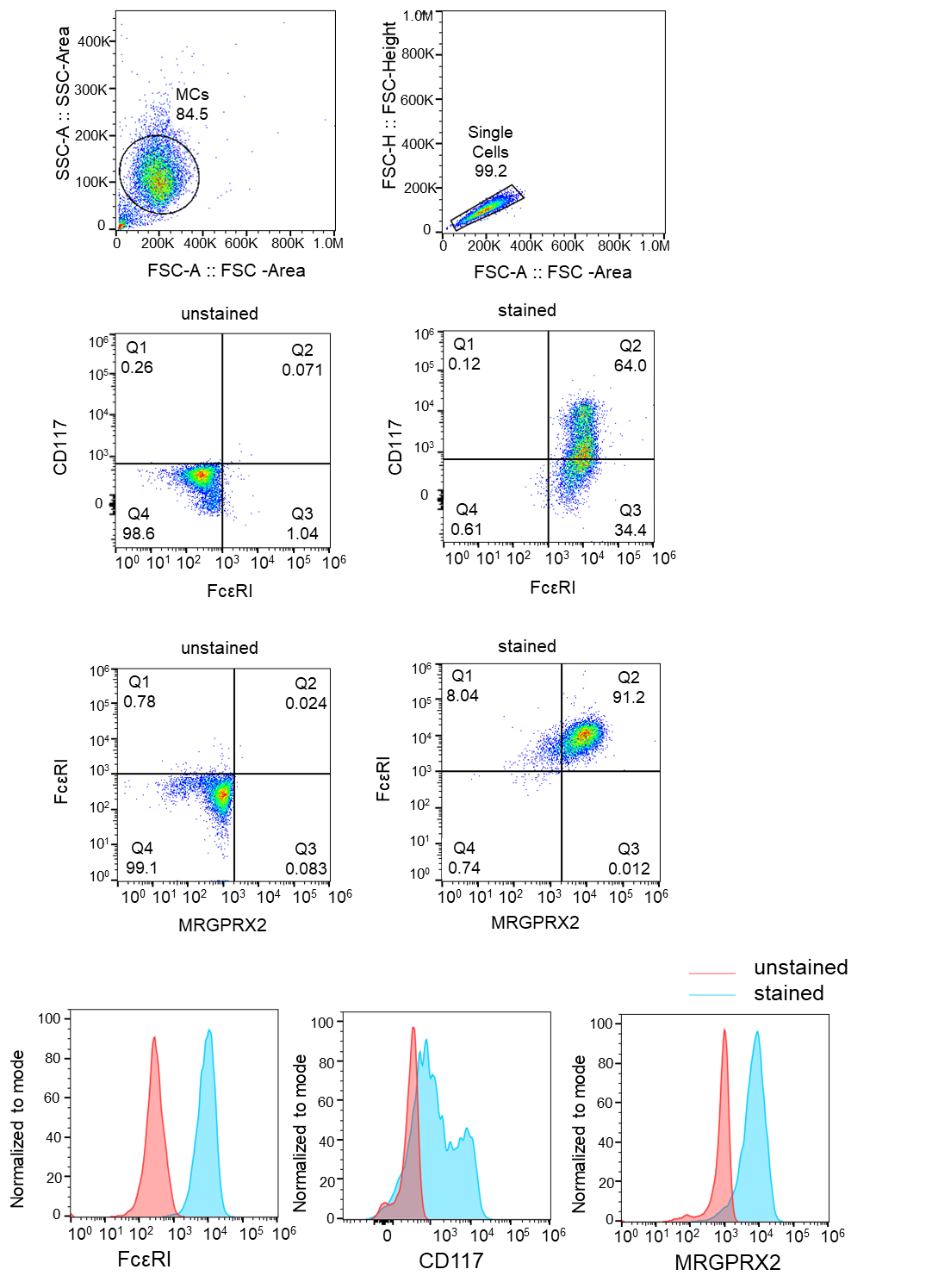


Figure. S6.

Flow cytometry gating strategy of primary human skin MCs. MCs were gated according to size and granularity in the FSC/SSC plot, excluding debris. Doublets were excluded in the FSC-A vs FSC-H and SSC-A vs SSC-H gate. Within the single cell gate, MCs were identified as FcεRI and CD117 (c-KIT) double-positive cells.


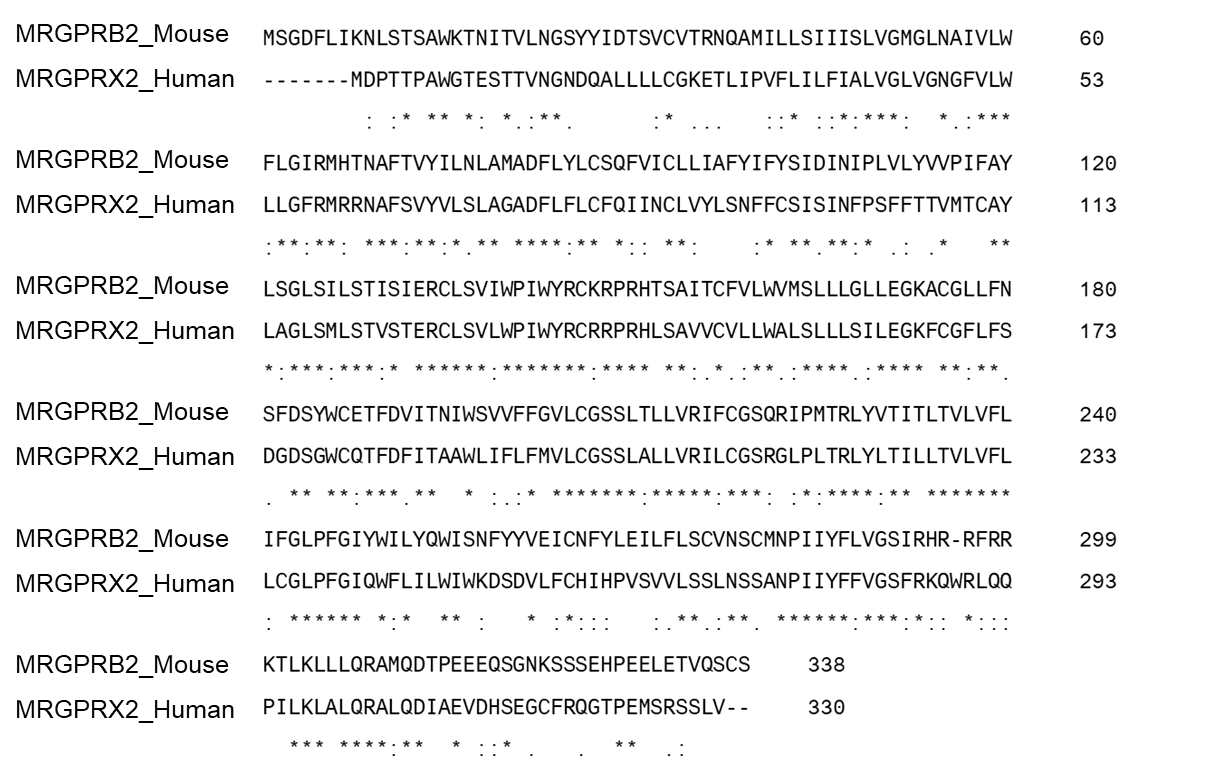


Figure. S7.

Sequence alignment and comparison of the antagonist binding pockets of both MRGPRX2 and MRGPRB2. * Denotes conserved sequence (identical amino acid residues), : denotes conservative mutation, ∙ denotes semi-conservative mutation, . denotes no similarity.


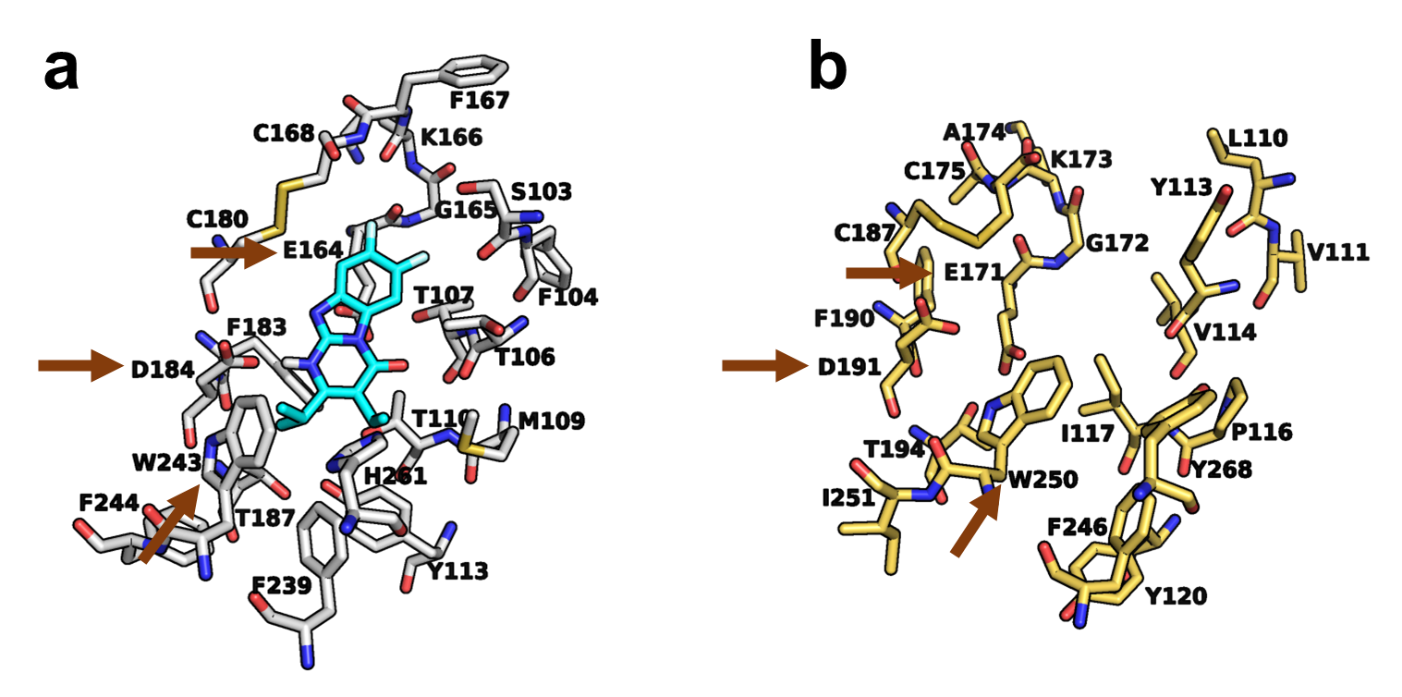


Figure. S8.

Comparison of PSB-172656 binding pockets of human MRGPRX2 and mouse MRGPRB2 **a** Docked pose of PSB-172656 (carbon colored cyan) within the putative orthostatic binding pocket of the human MRGPRX2 (depicted in gray, PDB: 7S8L). **b** Homology model of the mouse MRGPRB2, constructed based on the cryo-EM template of MRGPRX2. Brown arrows highlight amino acid residues that are suggested to interact with PSB-172656.

Table S1.

Expression of MRGPRX subtypes in LN229 cells determined using quantitative polymerase chain reaction (qPCR^h,i^)

|  | Cycle quantification (Cq) value^a^ |
| --- | --- |
| MRGPRX1^b^ | 45 |
| MRGPRX2^c^ | not detectable |
| MRGPRX3^d^ | 32.2 |
| MRGPRX4^e^ | 29.6 |
| GAPDH^f^ | 21.2 |
| *β*-actin^g^ | 19.4 |

^a^ The lower the Cq value the higher the expression

^b^ Primer set for MRGPRX1 (f: caccagctgtatgatctctgattc, r: caccagctgtatgatctctgattc)

^c^ Primer set for MRGPRX2 (f: cagtgtctctggaactgcctta, r: ttggcaaagctctcataggg)

^d^ Primer set for MRGPRX3 (f: tcgtctgagatgtggggag, r: ttcaggagatgcctacaggag)

^e^ Primer set for MRGPRX4 (f: aaacgtcagatttcatcccagt, r: cagaggatcctgaccagcag)

^f^ Primer set for human Glyceraldehyde 3-phosphate dehydrogenase (GAPDH) (f: agccacatcgctcagacac, r: gcccaatacgaccaaatcc);

^g^ Primer set for human *β*-actin (f: ggtggcttttaggatggcaag, r: actggaacggtgaaggtgacag)

^h^ Used kits: RNA purification (Qiagen), DNAase I (NEB), cDNA synthesis: iScript cDNA Synthesis Kit (BioRad), RNaseH (NEB), qRT PCR: iQ SYBR Green Supermix (BioRad) > (5 ng per reaction, and 250 nM primer per reaction.

^i^ Reaction program:

step 1: 95°C 3min, step 2: 95°C 20 s, step 3: 60°C 20 s, step 4: 68°C 40 s (repeat step2-4 49x), step 5: 95°C 30 s, step 6: 65°C 30 s, step 7: melt curve from 60°C to 95°C with an increment of 0.5°C/ 5 s

Table S2.

Potency of tricyclic pyrimido[1,2-*a*]benzimidazole derivatives

| **Compound** | **Ca^2+^ assay**^a^  ***K*_i_ ± SEM (nM)^b,c^** | ***β*-Arrestin assay^d^**  ***K*_i_ ± SEM**  **(nM)^e,c^** | ***β*-Arrestin assay^d^**  **EC_50_ ± SEM (nM)**  **(% activation)^f^** |
| --- | --- | --- | --- |
| **Quercetin** | (IC_50_ **46,100** ± 9,800) | (IC_50_ **71,200** ± 24,400) | n.d. |
| **1 (Hit)** | **246** ± 125 | **788** ± 143 | >10,000 (1%) |
| **2** | **238** ± 73 | **1,052** ± 234 | >10,000 (-2%) |
| **3** | **1,040** ± 17 | **3,778** ± 2410 | >10,000 (1%) |
| **4** | n.d. | n.d. | >10,000 (0%) |
| **5** | n.d. | n.d. | >10,000 (-1%) |
| **6** | **4,730** ± 866 | **4,788** ± 846 | >10,000 (1%) |
| **7** | **4,260** ± 1,371 | **1,048** ± 534 | >10,000 (0%) |
| **8** | **25.8** ± 7.8 | **90.5** ± 7.03 | >10,000 (-1%) |
| **9** | **16.6** ± 10.9 | **69.7** ± 20.8 | >10,000 (-1%) |
| **10** | **0.895** ± 0.54 | **94.4** ± 63.8 | >10,000 (-1%) |
| **11** | **43.3** ± 24.5 | **166** ± 69.7 | >10,000 (1%) |
| **12** | **18.5** ± 13.7 | **152** ± 33.8 | >10,000 (-2%) |
| **13** | **8.44** ± 4.8 | **113** ± 42.3 | >10,000 (-1%) |
| **14** | **69.7** ± 28.8 | **384** ± 214 | >10,000 (0%) |
| **15** | **364** ± 101 | **175** ± 49.5 | >10,000 (0%) |
| **16** | n.d. | n.d. | >10,000 (-2%) |
| **17** | n.d. | n.d. | >10,000 (-3%) |
| **18** | **1.42** ± 1.21 | **48.2** ± 1.08 | >10,000 (-1%) |
| **19** | n.d. | n.d. | >10,000 (-1%) |
| **20** | n.d. | n.d. | >10,000 (1%) |
| **21** | n.d. | n.d. | >10,000 (-1%) |
| **22** | n.d. | n.d. | >10,000 (0%) |
| **23** | n.d. | n.d. | >10,000 (-1%) |
| **24** | **0.142** ± 0.049 | **6.80** ± 3.51 | >10,000 (-1%) |
| **25** | **2.98** ± 0.59 | **155** ± 56.6 | >10,000 (1%) |
| **26** | **13.3** ± 6.1 | **289** ± 119 | >10,000 (-1%) |
| **27** | **6.64** ± 3.89 | **28.5** ± 23.9 | >10,000 (0%) |
| **28** | **0.322** ± 0.391 | **47.8** ± 9.77 | >10,000 (-3%) |
| **29** | **0.161** ± 0.102 | **29.6** ± 19.0 | >10,000 (-2%) |
| **30** | **0.455** ± 0.209 | **5.89** ± 2.86 | >10,000 (-3%) |
| **31** | n.d. | n.d. | >10,000 (1%) |
| **32** | **931** ± 685 | **824** ± 553 | >10,000 (-3%) |
| **33** | **906** ± 137 | **1,610** ± 885 | n.d. |

**^a^** LN229 cells recombinantly expressing MRGPRX2

**^b^** From three independent experiment in duplicates versus CST-14 (EC_80_ 800 nM, EC_50_ 452 nM)

**^c^** *K*_i_ values were calculated from the IC_50_ values using the Cheng–Prusoff equation
*K*_i_= IC_50_/ ([A]/EC_50_ +1) where, [A] is the fixed concentration of agonist and here A corresponds to EC_80_ of the agonist.

**^d^** *β*-Arrestin CHO cells recombinantly expressing MRGPRX2

**^e^** Data from three independent experiment in duplicates versus CST-14 (EC_80_ 1,000 nM, EC_50_ 483 nM)

**^f^** Percentage of activation of normalized to CST-14 (EC_80_ 5,000 nM)

Table S3

Plasma protein binding, metabolic stability, calculated parameters, and *in vitro* cytotoxicity of selected MRGPRX2 antagonists

| **Compound** | **24** (PSB-172656) | **27** | **30** | **33** |
| --- | --- | --- | --- | --- |
| **Structure** |  |  |  |  |
| **MW** | 291.3 | 238.4 | 273.3 | 274.3 |
| **Ca^2+^ assay^a^**  ***K*_i_ ± SEM (nM)^b^** | **0.142** ± 0.049 | **6.64** ± 3.89 | **0.455** ± 0.209 | **906** ± 137 |
| **Cytotoxicity** **CC_50_^c^ (nM) (% effect)** | > 10,000 (41%) | > 10,000 (28%) | > 10,000 (30%) | not determined |
| **PPB (%)^d^** | 100 | 99.7 | 100 | 99.8 |
| **T_1/2_^e,g^ (min)** | 92.4 | 10.6 | 14.0 | 48.8 |
| **Cl_int_^f,g^** | 15.0 | 130.8 | 100.4 | 28.4 |
| **Metabolic stability^g^** |  | | | |

^a^ LN229 cells recombinantly expressing MRGPRX2

^b^ From three independent experiment in duplicates versus CST-14 (EC_80_), *K*_i_ values were calculated from the IC50 values using the Cheng–Prusoff equation

^c^ Concentration of cytotoxicity 50% (**CC_50_**^)^ Determined in 3-(4,5-dimethylthiazol-2-yl)-2,5-diphenyl-2*H*-tetrazolium bromide  (MTT) assays performed in human 1321N1 astrocytoma cells; from 3 biological replicates, in triplicates ^d^ Plasma protein binding (PPB) (normalized to warfarin, which was used as control full PPB) ^d^ Calculated half-life (t_1/2_ = 0.693/slope= 92 min) ^e^ Intrinsic clearance rate (CL_int_ = 15 µL/min/mg = volume of distribution*0.693/ t_1/2_)
^g^ Human liver microsomes (0.25 mg/ml, mixed genders, pooled) were used to determine the stability of the compounds (1 µM) and the remaining of the compounds were measured using HPLC-MS/MS and normalized to the starting concentration, this was used to determine t_1/2_ and Cl_int_.

Data S1. (separate file)

Design and synthesis of analogs

Data S2. (separate file)

Homology model of MRGPRX2
