# Supplementary material for: Subnanomolar MAS-related G protein-coupled receptor-X2/B2 antagonists with efficacy in human mast cells and disease models
Source: Signal Transduct Target Ther. 2025 Apr 21;10:128. doi: 10.1038/s41392-025-02209-8 (PMC12010006; doi:10.1038/s41392-025-02209-8)
Supplement: Supplementary file 2 — Supplementary Data S1 [file 41392_2025_2209_MOESM2_ESM.docx]

Data S1: Design and synthesis of analogs

## Design and synthesis of analogs

The pyrimido[1,2-*a*]benzimidazole scaffold can be subdivided into its three rings, the pyrimidinone (**A**), the imidazole (**B**), and the benzene ring (**C**). Substitution of rings **A**, **B**, and **C** was systematically modified to study the structure-activity relationships (SARs) of the novel MRGPRX2 antagonists.

**Modification of the pyrimidinone (ring A)**

A series of pyrimido[1,2-*a*]benzimidazole derivatives with modification of the substituents on the pyrimidinone ring **A** was synthesized by cyclization of the commercially available 2-aminobenzimidazole (**34**) with the appropriate 3-oxocarboxylic acid esters (**35a-c**, **36a-d**, or **37a-e**) at elevated temperature, either with or without sodium alkoxide as a base yielding derivatives **4** and **6**-**16** (Scheme 1).^1–3^

The commercial availability of *α*-substituted-3-oxoesters was limited. Thus, they were synthesized from the commercially available 3-oxoesters **35b** and **35c** via alkylation at the *α*-position by treatment with a strong base and a suitable alkyl halide, yielding the derivatives **36a-d** (Scheme 1).^3^ The disadvantage of this method was, however, that the reaction did not always reach completion; starting material and desired product were difficult to separate requiring multiple rounds of flash chromatography. Alternatively, starting from ethyl butyrate (**39**), a modified mixed Claisen condensation reaction with a substituted carboxylic acid chloride (**38a-e**) afforded the desired *α*-substituted-3-oxoesters **37a-e** (Scheme 1).^3^ Advantages of this approach included the wide variety of commercially available carboxylic acid chlorides and the straightforward purification of the final products.

To synthesize the unsubstituted benzo[4,5]imidazo[1,2-*α*]pyrimidin-4(1*H*)-one **5**, 2-aminobenzimidazole (**34**) was treated with diethyl ethoxymethylenemalonate (**40**).^3^ Basic hydrolysis of the ethyl ester of product **41**, followed by decarboxylation of **42** yielded the desired compound **5** (Scheme 1).

Analogs with a thione in position 4 were obtained by treating compounds **4** and **7** either with phosphorus pentasulfide or with Lawesson's reagent to generate **17** and **18**. Compound **18** was subsequently treated with dimethyl sulfate under basic conditions leading to the generation of thioether **19** (Scheme 1).

**Scheme 1.** Synthesis of analogs of hit compound **1** with modifications at the pyrimidinone ring^a^

^a^Reagents and conditions: **A.** (a) **34**, *N,N*-dimethylformamide (DMF), reflux, overnight or NaOEt, EtOH, reflux; (b) NaH, R^2^-I, tetrahydrofuran (THF), rt, 30 min, reflux 3 h; (c) *n*BuLi, *i*Pr_2_NH, THF, -78 °C to rt, 3 h. **B.** (a) **34**, MeOH, reflux, 5h; (b) 0.2 M NaOH, 85 °C, 2.5 h; (c) Cu, quinoline, 200 °C, 1 h. **C.** (a) P_2_S_5_, pyridine, reflux, 20 h; (b) Lawesson's reagent, toluene, reflux, overnight; (c) Me_2_SO_4_, NaHCO_3_, acetone, reflux, 20 h.

**Modification of the imidazole moiety (ring B)**

To probe the requirement of a hydrogen bond donor, a benzo[4,5]imidazo[1,2-*a*]pyrimidin-4(10*H*)-one derivative with *N*-methyl substitution was obtained (compound **21**; Scheme 2), by cyclization of ethyl 4-methyl-3-oxopentanoate (**35c**) with *N*-methyl-2-amino-benzimidazole (**43**) (Scheme 2).

Compounds **22** and **23**, possessing an oxygen or sulfur atom instead of NH at the 10-position, were synthesized by cyclization of commercially available 2-aminobenzoxazole (**44a**) or 2-aminobenzothiazole (**44b**) with ethyl 3-oxobutanoate (**35a**) (Scheme 2).

**Scheme 2.** Synthesis of analogs of hit compound **1** with modifications at the imidazole ring^a^

^a^Reagents and conditions: **A.** (a) DMF, reflux, overnight. **B.** (a) polyphosphoric acid (PPA), 120° C, overnight.

**Modification of the benzene moiety (ring C)**

Various substituted 2-aminobenzimidazole derivatives **46a-e** were obtained by reaction of diaminobenzenes **45a-e** with cyanogen bromide. Subsequent condensation with ethyl 2-ethyl-4-methyl-3-oxopentanoate (**36c**) afforded the 7,8-disubstituted benzo[4,5]imidazo[1,2-*α*]pyrimidin-4(1*H*)-one derivatives **24-27** and 9-substituted benzo[4,5]imidazo[1,2-*α*]pyrimidin-4(1*H*)-one derivative **31** (Scheme 3).

**Scheme 3.** Synthesis of 7,8-disubstituted-benzo[4,5]imidazo[1,2-*a*]pyrimidin-4(1*H*)-ones **24-27** and 9-substituted benzo[4,5]imidazo[1,2-*α*]pyrimidin-4(1*H*)-one derivative **31**^a^

^a^Reagents and conditions: **A.** (a) BrCN, EtOH, 50 °C, overnight; (b) ethyl 2-ethyl-4-methyl-3-oxopentanoate (**36c**), DMF, reflux, overnight.

In order to obtain pure 7- and 8-monosubstituted derivatives, a novel synthetic pathway was established (Scheme 4). The amino group of commercially available substituted 2-nitroanilines **47a-d** was protected by a benzyl residue (**48a-d**). Catalytic hydrogenation of the nitro group of **48a-d** yielded the benzyl-protected 1,2-diaminobenzenes **49a-d**. Subsequent formation of the imidazole moiety was achieved by reaction of **49a-d** with cyanogen bromide affording the benzyl-protected 2-aminobenzimidazole derivatives **50a-d**. Condensation with the appropriate ethyl 2-ethyl-4-methyl-3-oxopentanoate (**36c**) yielded the 7- and 8-monosubstituted benzyl-protected benzo[4,5]imidazo[1,2*-α*]pyrimidin-4(10*H*)-ones **51a**, **51b**, and **51d** in a regioselective manner. Subsequent removal of the benzyl group by catalytic hydrogenation with palladium on carbon furnished the desired monosubstituted derivatives **28-30** (Scheme 4). Even though 3-ethyl-8-fluoro-2-isopropylbenzo[4,5]imidazo[1,2-*a*]pyrimidin-4(1*H*)-one (**30**) was successfully synthesized, the corresponding 7-fluoro-substituted regioisomer could not be obtained during the final cyclization with the ketoester. This may be explained by the inductive effect of the fluorine atom on the imidazole nitrogen lone pair.

For the synthesis of pyridyl analogs in which the benzene ring (**C**) of the pyrimido[1,2*-α*]benzimidazole scaffold was replaced by a pyridine, commercially available diaminopyridine derivative was used as starting material (**56a**). The 2,3-diamino-6-fluoropyridine (**56b**) was synthesized by protecting the amino group of 6-fluoro-substituted pyridine-3-amine (**52**) as a carbamate (**53**), followed by a regioselective aromatic nitration yielding **54**. The deprotection of the amino group was performed under basic conditions (**55**). Subsequent reduction of the nitro group with hydrogen gas in the presence of palladium on carbon as a catalyst afforded the required diaminopyridine derivative **56b**.

Ring closure was carried out using cyanogen bromide, yielding 1*H*-imidazo[4,5-*b*]pyridin-2-amines **57a-b**, followed by cyclization using ethyl 2-ethyl-4-methyl-3-oxopentanoate (**36c**) to obtain the final products **32-33** in moderate yields (Scheme 4).

**Scheme 4**. Synthesis of 7- and 8-monosubstituted-benzo[4,5]imidazo[1,2-*a*]pyrimidin-4(1*H*)-ones **28-30** and their pyridyl analogs **32-33** ^a^

^a^Reagents and conditions: **A.** (a) BnBr, H_2_O, reflux, 48 h; (b) Raney nickel, H_2_, MeOH, rt, 3 h; (c) BrCN, EtOH, 50 °C, overnight; (d) DMF, ethyl 2-ethyl-4-methyl-3-oxopentanoate (**36c**), 160 °C, overnight; (e) H_2_, Pd/C, CH_2_Cl_2_, MeOH, rt, 5 h. **B.** (a) propyl chloroformate, NaHCO_3_, THF, 0 °C, 30 min; (b) H_2_SO_4_, HNO_3_, 0 °C to rt; (c) Ba(OH)_2_, 2n NaOH, acetonitrile, 90 °C, overnight; (d) H_2_, 10% Pd/C, MeOH, rt; (e) BrCN, MeOH/H_2_O, 90 °C, 1 h; (f) DMF, *N*,*N*-diisopropylethylamine (DIPEA), ethyl 2-ethyl-4-methyl-3-oxopentanoate (**36c**), 160 °C.

All synthesized final compounds were characterized by NMR spectroscopy and high-resolution mass spectrometry (HRMS). Purity was determined by high-performance liquid chromatography-UV(256 nm)-low resolution mass spectra (HPLC‑MS) and found to be >95% in all cases.

The additional compounds **2**, **3** and **20** were commercially available and included in the SAR study (Table S2).

## Materials

All commercial reagents were obtained from Acros Organics, Sigma-Aldrich, AK Scientific and Fluorochem and are at least 99% pure unless indicated otherwise. All dry solvents were purchased from Acros Organics with an AcroSeal system and regular solvents were obtained via Fisher Scientific at analytical grade. 3-Ethyl-1,2-dimethylpyrimido[1,2-*a*]benzimidazol-4(1*H*)-one (**20**) was purchased from ChemBridge (ID number: 9205102) (San Diego, CA, USA). Thin layer chromatography (TLC) was performed on silica gel on aluminum foils with fluorescent indicator (254 nm) (60 Å pore diameter) obtained from Sigma-Aldrich and visualized using ultraviolet light (254 nm). Recording of the NMR spectra was performed using a Bruker 300 MHz, 500 MHz or 600 MHz spectrometer. The chemical shifts are reported in ppm relative to tetramethylsilane (TMS) or the residual solvent signal for ^1^H, or the residual solvent signal for ^13^C. Spectra recorded in D_2_O and for ^19^F are uncorrected. Coupling constants (J) are reported in Hertz. Mass spectra were acquired on a quadrupole orthogonal acceleration time-of-flight mass spectrometer (Synapt G2 HDMS, Waters, Milford, MA). Samples were infused at 3 µL/min and spectra were obtained in positive or negative ionization mode with a resolution of 15000 (FWHM) using leucine enkephalin as lock mass. Purity of the compounds was determined on a Waters 600 HPLC system equipped with a Waters 2487 Dual λ absorbance detector set at 256 nm using a 5 µm 4.6x150 mm XBridge Reversed Phase (C18) column. The mobile phase was a gradient over 30 min starting from 95% A and 5% B and finishing at 5% A and 95% B with a flow rate of 1 ml/min (solvent A: MilliQ water; solvent B: acetonitrile). All synthesized final compounds had a purity of at least 95%. Compound **33** was crystallized from a tetrahydrofuran (THF) solution at room temperature. Measurement was performed using a Bruker X8-KappaApexII instrument and the analysis of the resulting crystal structure showed a triclinic crystal system within the space group P-1.

## Tautomerism of benzo[4,5]imidazo[1,2-a]pyrimidin-4-one derivatives

The benzo[4,5]imidazo[1,2-*a*]pyrimidin-4-one derivatives may undergo tautomerism, with the hydrogen atom being adjacent to *N*1 or *N*10 as shown for derivative **5** (Supplementary Data 1 Fig. SD1a). We obtained a crystal structure of compound **33** displaying the tautomeric *N*1-H structure. The crystal was stabilized by intermolecular hydrogen bonds (Supplementary Data 1 Fig. SD1b). Additionally, ^1^H-NMR spectroscopy of compound **33** in deuterated dimethyl sulfoxide (DMSO-*d*_6_) showed a broad singlet (br s) at a chemical shift of 12.73 ppm indicating for the presence of the *N*1-H tautomeric structure (Supplementary Data 1 Fig. SD1c).

**Fig.** SD1 Tautomerism of pyrimido[1,2-*a*]benzimidazoles **a** Tautomeric forms of tricyclic pyrimido[1,2-*a*]benzimidazoles shown for compound **5**. **b** Crystal structure obtained from a tetrahydrofuran (THF) solution at room temperature and the molecular structure of compound **33. c**^1^H-NMR spectrum of compound **33**.

## Chemistry

**General procedure A:** Synthesis of 2-alkyl-3-oxoesters (**36a,** **36c-36d**). Appropriate 3-oxoester (**35b-35c**; 1.0 equiv) was added to a suspension of NaH (60% in mineral oil, 1.0 equiv) in THF (1.0 ml/1.0 mmol of 3-oxoester) at rt and the resulting mixture was stirred for 30 min at rt. Then the alkyl halide (1.0 equiv) was added, and the mixture was refluxed for 3 h. After cooling to rt, the reaction was quenched with saturated aqueous NH_4_Cl solution (1.2 ml/1.0 mmol of NaH). The aqueous layer was separated and extracted with ethyl acetate (3x). The organic layers were combined, dried over MgSO_4_, filtered, and concentrated under reduced pressure. The crude product was purified by flash column chromatography on silica gel giving colorless liquid.

**General procedure B:** Synthesis of 2-alkyl-3-oxoesters (**37a-37e**). To a solution of butyl lithium (2.3 equiv) in THF at -78 °C was added diisopropylamine (DIPEA; 2.4 equiv) dropwise. The mixture was stirred at -78 °C for 30 min. Ethyl ester (**39**; 2.0 equiv) was added dropwise via cannula over 30 min and the mixture was allowed to stir further at –78 °C. Lastly acid chloride (**38a-38e**; 1.0 equiv) was added dropwise to the reaction mixture and after addition the reaction was heated to rt and allowed to stir for another 2 h. After completion of the reaction was confirmed by TLC, the reaction was quenched with saturated aqueous NH_4_Cl solution, and the mixture was extracted with ethyl acetate (3x). The combined organic layers were dried over Na_2_SO_4_, filtered and concentrated under reduced pressure. The crude product was purified by flash column chromatography on silica gel yielding a colorless to light yellow oil.

**General procedure C:** Synthesis of 2(,3)-(di)substituted-benzo[4,5]imidazo[1,2-a]pyrimidin-4(1*H*)-ones (**4, 8, 10, 12-16, 21**) and 6(,7)-(di)substituted-benzo[4,5]imidazo[1,2-a]pyrimidin-4(1*H*)-ones (**24-25, 27, 31, 51a-51b, 51d**). To an appropriate 2-aminoimidazole derivative (1.0 equiv) in DMF (1.5 ml/1 mmol of starting material) was added the appropriate 3-oxoester (1-1.64 equiv) and the mixture was refluxed overnight. Upon disappearance of the starting materials or no further progress of the reaction, the mixture was cooled to rt. If precipitation occurred MeOH or EtOH was added, the solid was filtered off, washed with the indicated solvent and dried under vacuum. In other cases, the volatiles were removed, and the crude residue was purified by silica gel column chromatography.

**General procedure D:** Synthesis of 2,3-disubstituted-benzo[4,5]imidazo[1,2-a]pyrimidin-4(1*H*)-ones (**9**, **11**). To a mixture of an appropriate 2-aminoimidazole derivative (1.0 equiv) and 3-oxoester (1.0-1.1 equiv) in absolute EtOH (3 ml/1 mmol of starting material) was added sodium ethoxide (21% in EtOH; 2.0 equiv). Resulting mixture was refluxed for several hours (TLC control). Upon disappearance of starting materials or no further progress the reaction mixture was cooled to rt. Volatiles were removed under reduced pressure and the crude product was purified using flash silica gel column chromatography or preparative TLC. In case of not sufficient purity of product after chromatographic purification, the solid was suspended in MeOH, filtered and dried affording the target compound.

**General procedure E:** Synthesis of 1*H*-benzo[d]imidazol-2-amine derivatives (**46a-46e, 50a-50d**). To an appropriate diaminobenzene derivative (1.0 equiv) in EtOH (4.5 ml/1 mmol starting material) was added BrCN (1.2 equiv) and the resulting mixture was stirred at 50 °C till disappearance of the starting material (TLC control). Then, volatiles were removed under reduced pressure and the solid residue was redissolved (or resuspended) in H_2_O. The aqueous mixture was extracted with EtOAc (3x). The organic layer was discarded, and the pH of the aqueous layer was adjusted to 9–10 (according to universal indicator paper) using a 25% aqueous NH_4_OH or 1 M aqueous NaOH solution. The aqueous layer was extracted with EtOAc (3x). The combined organic layers were dried over MgSO_4_, filtered and the solvent was evaporated under reduced pressure and, if necessary, the crude residue was further purified by silica gel flash column chromatography.

**General procedure F:** Synthesis of *N*-benzyl-2-nitroanilines *(***48a-48d**). A mixture of substituted 2-nitroaniline (**47a-47d**; 1.0 equiv) and benzyl bromide (1.2 equiv) in H_2_O (20 ml) was stirred at reflux for 1.5 h. Then, an additional amount of benzyl bromide (3.95 mmol. 0.6 equiv) was added and stirring was continued at reflux for another 2 days. After cooling to rt, a saturated aqueous NaHCO_3_ solution was added, and the mixture was extracted with EtOAc (3x). The organic layers were combined, washed with H_2_O (1x) and dried over Na_2_SO_4_. After removal of drying agent and solvent, the crude residue was purified by silica gel flash column chromatography (using heptane/EtOAc 9:1 as mobile phase).

**General procedure G:** Synthesis of benzene-1,2-diamines (**49a-49d**). To a solution of substituted 2-nitroaniline (**48a-48d**; 1.0 equiv) in methanol (10 ml) was added a slurry of Raney nickel (30 mg) and the mixture was stirred vigorously. The system was flushed with H_2_ gas (3 x). The reaction was allowed to stir for 3 h till the reaction was completely finished. The catalyst was removed by filtration through Celite® and the filtrate was concentrated under reduced pressure yielding the compounds **49a-49d** in quantitative yield which was immediately used in the next reaction.

**General procedure H:** Deprotection of 10-benzyl-benzo[4,5]imidazo[1,2-a]pyrimidin-4(10*H*)-ones (**51a-51b, 51d**) towards the benzo[4,5]imidazo[1,2-a]pyrimidin-4(1*H*)-ones (**28-30**). To a solution of 10-benzyl-benzo[4,5]imidazo[1,2-*a*]pyrimidin-4(10*H*)-ones (**51a-51b, 51d**) in a mixture of DCM and MeOH (1:1, 5 ml) was added Pd/C catalyst (10 mol%). The reaction was flushed with H_2_ gas and stirred for 5 h. After reaction stagnation, the mixture was filtered through Celite® and the solvent was evaporated. The crude residue was purified by silica gel flash column chromatography (using heptane/EtOAc in a ratio of 7:3 as mobile phase).

**General procedure I:** Synthesis of 1*H*-imidazo[4,5-b]pyridin-2-amines (**57a-57b**). The diamino substituted pyridine derivatives (**56a-56b**; 1.0 equiv) and BrCN (3.0 equiv) were dissolved in a mixture of MeOH/H_2_O (1:1, 4 ml) and heated to 90 °C for 4-6 h. After complete conversion of the starting material (TLC: 20% MeOH in CH_2_Cl_2_) the solvent was removed under reduced pressure and the residue purified by automated flash chromatography (gradient: 0-20% MeOH in CH_2_Cl_2_) to yield the 1*H*-imidazo[4,5-*b*]pyridin-2-amine derivatives **57a-57b**.

**General procedure J:** Synthesis of pyrido[2',3':4,5]imidazo[1,2-a]pyrimidin-6(9*H*)-one derivatives (**32-33**). The 1*H*-imidazo[4,5-*b*]pyridin-2-amine derivatives (**57a-57b;** 1.0 equiv) and DIPEA (2.0 equiv) were dissolved in a minimum amount of DMF (2-3 ml) and ethyl 2-ethyl-4-methyl-3-oxopentanoate (**36c**; 2.0 equiv) was added in portions. The resulting mixture was heated to 160 °C for 18 h until complete conversion of the starting material (TLC: 5% MeOH in CH_2_Cl_2_). The solvent was removed under reduced pressure, and the residue purified by automated flash chromatography (gradient: 0-10% MeOH in CH_2_Cl_2_, 20 min). An additional reversed-phase HPLC separation (gradient: 60-100% MeOH in H_2_O, 20 min) was required to yield the final product.

*Ethyl 2-ethyl-3-oxohexanoate (****36a****).* Ethyl 2-ethyl-3-oxohexanoate (**36a**) was obtained from ethyl 3-oxohexanoate (**35b**) and ethyl iodide according to General procedure A. Yield: 76%, colorless liquid. ^1^H-NMR (300 MHz, CDCl_3_) δ [ppm] = 4.19 (q, *J* = 7.2 Hz, 2H), 3.35 (t, *J* = 7.2 Hz, 1H), 2.54 (m, 1H), 2.44 (m, 1H), 1.92 - 1.83 (m, 2H), 1.62 (sext, *J* = 7.5 Hz, 2H), 1.27 (t, *J* = 7.2 Hz, 3H), 0.92 (t, *J* = 7.5 Hz, 3H), 0.91 (t, *J* = 7.5 Hz, 3H). ^13^C-NMR (75 MHz, CDCl_3_) δ [ppm] = 205.5, 170.0, 61.3, 60.8, 43.9, 21.7, 17.0, 14.2, 13.7, 12.1.

*Ethyl 3-oxo-2-propylhexanoate (****36b****).* To a mixture of ethyl 3-oxohexanoate (**35b**, 1.0 mmol) in THF (5 ml) was added *t-*BuOK (1.1 mmol) and the mixture was stirred for 15 min at rt. Then propyl iodide (1.0 mmol) was added dropwise, and the mixture was stirred at rt overnight. After that time TLC control showed presence of mainly starting material. The mixture was brought to reflux and kept refluxing for 20 h. Then it was poured into a saturated aqueous NH_4_Cl solution (50 ml) and extracted with ethyl acetate (3x). The organic layers were combined, dried over MgSO_4_, filtered and concentrated under reduced pressure. The crude product was purified by flash column chromatography on silica gel (CH_2_Cl_2_) affording 99 mg of a colorless liquid (**36b**, 0.494 mmol, 49%). ^1^H-NMR (300 MHz, CDCl_3_) δ [ppm] = 4.18 (q, *J* = 7.2 Hz, 2H), 3.46 (t, *J* = 7.5 Hz, 1H), 1.75 – 1.70 (m, 2H), 1.66 - 1.51 (m, 2H), 1.37 - 1.24 (m, 5H), 0.92 (t, *J* = 7.2 Hz, 3H), 0.91 (t, *J* = 7.5 Hz, 3H). ^13^C-NMR (75 MHz, CDCl_3_) δ [ppm] = 204.5, 169.1, 60.3, 58.1, 42.8, 29.4, 19.8, 16.0, 13.2, 12.9, 12.7.

*Ethyl 2-ethyl-4-methyl-3-oxopentanoate (****36c****).* Ethyl 2-ethyl-4-methyl-3-oxopentanoate (**36c**) was obtained from ethyl *iso*butyrylacetate (**35c**) and ethyl iodide according to General procedure A. Yield: 90%, colorless liquid. ^1^H-NMR (300 MHz, CDCl_3_) δ [ppm] = 4.18 (q, *J* = 7.1 Hz, 2H), 3.53 (t, *J* = 7.3 Hz, 3H), 2.80 (sept, *J* = 6.9 Hz, 1H), 1.87 (dq, *J* = 7.4, 7.3 Hz, 2H), 1.26 (t, *J* = 7.1 Hz, 3H), 1.12 (d, *J* = 6.9 Hz, 3H), 1.10 (d, *J* = 6.9 Hz, 3H), 0.92 (t, *J* = 7.4 Hz, 3H). ^13^C-NMR (75 MHz, CDCl_3_) δ [ppm] = 209.2, 169.9, 61.3, 58.8, 40.7, 21.8, 18.5, 18.2, 14.2, 12.2.

*Ethyl 4-methyl-3-oxo-2-propylpentanoate (****36d****).* Ethyl 4-methyl-3-oxo-2-propylpentanoate (**36d**) was obtained from ethyl *iso*butyrylacetate (**35c**) and propyl iodide according to General procedure A. Yield: 68%, colorless liquid. ^1^H-NMR (300 MHz, CDCl_3_) δ [ppm] = 4.17 (q, *J* = 7.1 Hz, 2H), 3.61 (t, *J* = 7.3 Hz, 1H), 2.77 (sept, *J* = 6.9 Hz, 1H), 1.86 – 1.78 (m, 2H), 1.34 - 1.23 (m, 5H), 1.11 (d, *J* = 6.9 Hz, 3H), 1.10 (d, *J* = 6.9 Hz, 3H), 0.93 (t, *J* = 7.3 Hz, 3H). ^13^C-NMR (75 MHz, CDCl_3_) δ [ppm] = 208.3, 169.0, 60.3, 56.1, 39.7, 29.5, 17.5, 17.2, 13.2, 13.0.

*Ethyl 2-ethyl-5-methyl-3-oxohexanoate (****37a****).* Ethyl 2-ethyl-5-methyl-3-oxohexanoate (**37a**) was obtained from *iso*valeryl chloride (**38a**; 0.50 g, 4.15 mmol) and ethyl butanoate (**39**) according to General procedure B. Yield: 58%, colorless oil. ^1^H-NMR (300 MHz, CDCl_3_) δ [ppm] = 4.18 (dd, *J* = 8.7, 5.7 Hz, 2H), 2.55 - 2.39 (m, 1H), 1.94 - 1.68 (m, 2H), 1.55 (m, 3H), 1.28 (t, *J* = 7.2 Hz, 3H), 0.89 (m, 9H).

*Ethyl 2-ethyl-4,4-dimethyl-3-oxopentanoate (****37b****).* Ethyl 2-ethyl-4,4-dimethyl-3-oxopentanoate (**37b**) was obtained from trimethyacetyl chloride (**38b**; 0.50 g, 4.15 mmol) and ethyl butanoate (**39**) according to General procedure B. Yield: 38%, colorless oil. ^1^H-NMR (300 MHz, CDCl_3_) δ [ppm] = 4.13 (q, *J* = 7.1 Hz, 2H), 3.80 (t, *J* = 7.1 Hz, 1H), 1.80 (m, 2H), 1.24 (t, *J* = 7.5 Hz, 3H), 1.17 (s, 9H), 0.91 (t, *J* = 7.4 Hz, 3H).

*Ethyl 2-(cyclopropanecarbonyl)butanoate (****37c****).* Ethyl 2-(cyclopropanecarbonyl)butanoate (**37c**) was obtained from cyclopropanecarbonyl chloride (**38c**; 0.50 g, 4.78 mmol) and ethyl butanoate (**39**) according to General procedure B. Yield: 26%, light-yellow oil. ^1^H-NMR (300 MHz, CDCl_3_) δ [ppm] = 4.21 (q, *J* = 7.1 Hz, 2H), 3.47 (t, *J* = 7.4 Hz, 1H), 2.07 (m, 1H), 1.94 (q, *J* = 7.4 Hz, 2H), 1.27 (t, *J* = 7.1 Hz, 3H), 1.09 (m, 2H), 0.95 (t, *J* = 7.4 Hz, 3H).

*Ethyl 2-benzoylbutanoate (****37d****).* Ethyl 2-benzoylbutanoate (**37d**) was obtained from benzoyl chloride (**38d**; 0.50 g, 3.56 mmol) and ethyl butanoate (**39**) according to General procedure B. The crude was used in the next reaction without further purification.

*Ethyl 2-(thiophene-2-carbonyl)butanoate (****37e****)*. Ethyl 2-(thiophene-2-carbonyl)butanoate (**37e**) was obtained from thiophene-2-carbonyl chloride (**38e**; 0.50 g, 3.41 mmol) and ethyl butanoate (**39**) according to General procedure B. Yield: 32%, colorless oil. ^1^H-NMR (300 MHz, CDCl_3_) δ [ppm] = 7.80 (s, 1H), 7.68 (s, 1H), 7.15 (s, 1H), 4.17 (m, 2H), 2.74 (m, 1H), 2.05 (m, 2H), 0.99 (t, J = 7.4 Hz, 3H), 0.81 (t, J = 7.5 Hz, 3H).

*2-Methylbenzo[4,5]imidazo[1,2-a]pyrimidin-4(1H)-one (****4****).* 2-Methylbenzo[4,5]imidazo[1,2-*a*]pyrimidin-4(1*H*)-one (**4**) was synthesized according to General procedure C starting from 2-aminobenzimidazole (**34**; 100 mg, 0.751 mmol, 1.0 equiv) and ethyl acetoacetate (**35a**; 1.0 equiv). Yield: 66%, off-white solid. ^1^H-NMR (300 MHz, DMSO-*d*_6_) δ [ppm] = 12.73 (br s, 1H), 8.37 (d, *J* = 7.5 Hz, 1H), 7.53 (d, 1H, *J* = 7.5 Hz), 7.43 (t, *J* = 7.5 Hz, 1H), 7.29 (t, *J* = 7.5 Hz, 1H), 5.84 (s, 1H), 2.31 (s, 3H). ^13^C-NMR (75 MHz, DMSO-*d*_6_) δ [ppm] = 159.3, 148.4, 135.4, 126.9, 125.6, 121.4, 115.2, 113.8, 98.5, 21.8. HRMS (ESI): *m/z* [M+H]^+^ calculated for C_11_H_10_N_3_O: 200.0818, found: 200.0822.

*2-Propylbenzo[4,5]imidazo[1,2-a]pyrimidin-4(1H)-one (****6****).*

This compound was commercially available from ChemBridge (San Diego, CA, USA), but its suggested synthesis procedure is detailed in Scheme 1

*2-Isopropylbenzo[4,5]imidazo[1,2-a]pyrimidin-4(1H)-one (****7****).* 2-Isopropylbenzo[4,5]imidazo[1,2-*a*]pyrimidin-4(1*H*)-one (**8**) was synthesized according to General procedure C starting from 2-aminobenzimidazole (**34,** 2.0 g, 15.02 mmol, 1.0 equiv) and ethyl 4-methyl-3-oxopentanoate (**35c**; 1.0 equiv). Yield: 59%, grey solid ^1^H NMR (DMSO-*d*_6_, 600 MHz): δ (ppm) 1.25 (d, 2H, *J* = 6.6 Hz), 2.84 (sept, 1H, *J* = 6.6 Hz), 5.88 (s, 1H), 7.30 (t, 1H, *J* = 7.8 Hz), 7.45 (t, 1H, *J* = 7.8 Hz), 7.49 (d, 1H, *J* = 7.8 Hz), 8.40 (d, 1H, *J* = 8.1 Hz), 12.86 (bs, 1H). ^13^C NMR (DMSO-*d*_6_, 150 MHz): δ (ppm) 21.48, 34.50, 96.54, 112.52, 115.40, 121.55, 125.83, 126.41, 133.44, 149.06, 159.78, 169.96. ^1^H NMR (CDCl_3_, 300 MHz): δ (ppm) 1.43 (d, 2H, *J* = 6.9 Hz), 3.11 (sept, 1H, *J* = 6.9 Hz), 6.04 (s, 1H), 7.36 (t, 1H, *J* = 8.1. Hz), 7.45–7.52 (m, 2H), 8.63 (d, 1H, *J* = 8.1 Hz). ^13^C NMR (CDCl_3_, 75 MHz): δ (ppm) 21.98, 34.41, 97.09, 113.05, 116.63, 122.56, 126.23, 127.49, 134.81, 149.31, 160.55, 167.18. HRMS (ESI): *m*/*z* [M+H]^+^ calcd for C_13_H_13_N_3_O: 228.1131, found: 228.1134.

*Ethyl-2-propylbenzo[4,5]imidazo[1,2-a]pyrimidin-4(1H)-one (****8****).* Ethyl-2-propylbenzo [4,5]imidazo[1,2-*a*]pyrimidin-4(1*H*)-one (**8**) was synthesized according to General procedure C starting from 2-aminobenzimidazole (**34**; 358 mg, 2.684 mmol, 1.0 equiv) and ethyl 2-ethyl-3-oxohexanoate (**36a**; 1.0 equiv). Yield: 47%, off-white solid. ^1^H-NMR (300 MHz, DMSO-*d*_6_) δ [ppm] = 12.55 (br s, 1H), 8.40 (d, *J* = 7.8 Hz, 1H), 7.47 (d, *J* = 8.4 Hz, 1H), 7.41 (td, *J* = 7.8, 1.2 Hz, 1H), 7.26 (td, *J* = 8.4, 1.2 Hz, 1H), 2.61-2.59 (m, 2H), 2.54 (q, *J* = 7.2 Hz, 2H), 1.69 (sext, *J* = 7.8 Hz, 2H), 1.08 (t, *J* = 7.5 Hz, 3H), 0.96 (t, *J* = 7.2 Hz, 3H). ^13^C-NMR (75 MHz, DMSO-*d*_6_) δ [ppm] = 159.7, 147.0, 126.8, 125.6, 121.1, 115.2, 113.3, 111.1, 34.6, 21.9, 18.1, 14.2, 13.9. HRMS (ESI): *m/z* [M+H]^+^ calculated for C_15_H_18_N_3_O: 256.1444, found: 256.1445.

*2,3-Dipropylbenzo[4,5]imidazo[1,2-a]pyrimidin-4(1H)-one (****9****).* 2,3-Dipropylbenzo [4,5]imidazo[1,2-*a*]pyrimidin-4(1*H*)-one (**9**) was synthesized according to General procedure D starting from 2-aminobenzimidazole (**34**; 66 mg, 0.494 mmol, 1.0 equiv) and ethyl 3-oxo-2-propylhexanoate (**36b**; 1.0 equiv). Yield: 30%, off-white solid. ^1^H-NMR (300 MHz, DMSO-*d*_6_) δ [ppm] = 12.48 (br s, 1H), 8.40 (d, *J* = 8.1 Hz, 1H), 7.48 (d, *J* = 7.8 Hz, 1H), 7.45-7.39 (m, 1H), 7.30-7.24 (m, 1H), 2.64-2.59 (m, 2H), 2.42-2.48 (m, 2H; overlapped with residual solvent signal), 1.70 (sext, *J* = 7.5 Hz, 2H), 1.50 (sext, *J* = 7.5 Hz, 2H), 1.00-0.93 (m, 6H). ^13^C-NMR (75 MHz, DMSO-*d*_6_) δ [ppm] = 159.8, 157.9, 146.9, 135.0, 126.7, 125.5, 121.0, 115.1, 113.1, 109.5, 34.5, 26.7, 22.4, 21.7, 14.0, 13.8. HRMS (ESI): *m/z* [M+H]^+^ calculated for C_16_H_20_N_3_O: 270.1601, found: 270.1599.

*3-Ethyl-2-isopropylbenzo[4,5]imidazo[1,2-a]pyrimidin-4(1H)-one (****10****).* 3-Ethyl-2-*iso*propyl benzo[4,5]imidazo[1,2-*a*]pyrimidin-4(1*H*)-one (**10**) was synthesized according to General procedure C starting from 2-aminobenzimidazole (**34**; 358 mg, 2.684 mmol, 1.0 equiv) and ethyl 2-ethyl-4-methyl-3-oxopentanoate (**36c**; 1.0 equiv). Yield: 67%, off‑white solid. ^1^H-NMR (500 MHz, pyridine-*d*_5_) δ [ppm] = 8.85 (d, *J* = 8.0 Hz, 1H), 7.47 (d, *J* = 8.0 Hz, 1H), 7.34 (t, *J* = 7.5 Hz, 1H), 7.21 (t, *J* = 7.5 Hz, 1H), 3.13 (sept, *J* = 6.5 Hz, 1H), 2.77 (q, *J* = 7.5 Hz, 2H), 1.27 (d, *J* = 6.5 Hz, 6H), 1.20 (t, *J* = 7.5 Hz, 3H). ^13^C-NMR (125 MHz, pyridine-*d*_5_) δ [ppm] = 166.6, 161.0, 148.8, 127.6, 126.2, 121.6, 116.7, 112.4, 111.8, 31.5, 22.0, 18.8, 15.2. HRMS (ESI): *m/z* [M+H]^+^ calculated for C_15_H_18_N_3_O: 256.1444, found: 256.1442.

*2-Isopropyl-3-propylbenzo[4,5]imidazo[1,2-a]pyrimidin-4(1H)-one (****11****).* 2-*Iso*propyl-3-propylbenzo[4,5]imidazo[1,2-*a*]pyrimidin-4(1*H*)-one (**11**) was synthesized according to General procedure D starting from 2-aminobenzimidazole (**34**; 168 mg, 1.258 mmol, 1.0 equiv) and ethyl 4-methyl-3-oxo-2-propylpentanoate (**36d**; 1.0 equiv). Yield: 25%, off-white solid. ^1^H-NMR (500 MHz, pyridine-*d*_5_) δ [ppm] = 8.93 (d, *J* = 7.8 Hz, 1H), 7.55 (d, *J* = 8.1 Hz, 1H), 7.42 (td, *J* = 7.8, 0.9 Hz, 1H), 7.29 (td, *J* = 8.1, 0.9 Hz, 1H), 3.27 (sept, *J* = 6.6 Hz, 1H), 2.86-2.81 (m, 2H), 1.75 (sext, *J* = 7.5 Hz, 2H), 1.36 (d, *J* = 6.6 Hz, 6H), 1.06 (t, *J* = 7.5 Hz, 3H). ^13^C-NMR (125 MHz, pyridine-*d*_5_) δ [ppm] = 166.9, 161.3, 149.0, 133.3, 127.7, 126.3, 121.8, 116.8, 111.9, 110.9, 31.7, 27.6, 24.0, 22.1, 14.7. HRMS (ESI): *m/z* [M+H]^+^ calculated for C_16_H_20_N_3_O: 270.1601, found: 270.1601.

*3-Ethyl-2-isobutylbenzo[4,5]imidazo[1,2-a]pyrimidin-4(1H)-on (****12****).* 3-Ethyl-2-*iso*butyl benzo[4,5]imidazo[1,2-*a*]pyrimidin-4(1*H*)-on (**12**) was synthesized according to General procedure C starting from 2-aminobenzimidazole (**34**; 300 mg, 2.25 mmol,1 .0 equiv) and ethyl 2-ethyl-5-methyl-3-oxohexanoate (**37a**; 1.1 equiv). Yield: 13%, white solid. ^1^H-NMR (500 MHz, pyridine-*d*_5_) δ [ppm] = 8.93 (d, *J* = 7.9 Hz, 1H), 7.62 (d, *J* = 8.0 Hz, 1H), 7.43 (t, *J* = 7.7 Hz, 1H), 7.31 (t, *J* = 7.7 Hz, 1H), 2.80 (q, *J* = 7.3 Hz, 2H), 2.62 (d, *J* = 7.3 Hz, 2H), 2.29 (sept, *J* = 6.9 Hz, 1H), 1.31 (t, *J* = 7.4 Hz, 3H), 0.98 (d, *J* = 6.6 Hz, 6H). ^13^C-NMR (75 MHz, pyridine-*d*_5_) δ [ppm] = 160.1, 156.4, 147.5, 136.0, 125.2, 120.8, 115.7, 113.1, 112.2, 41.5, 29.4, 28.1, 22.0, 18.7, 13.9. HRMS (ESI): *m/z* [M+H]^+^ calculated for C_16_H_20_N_3_O: 270.1607, found: 270.1606.

*2-(tert-Butyl)-3-ethylbenzo[4,5]imidazo[1,2-a]pyrimidin-4(1H)-one (****13****).* 2-(*tert*-Butyl)-3-ethylbenzo[4,5]imidazo[1,2-*a*]pyrimidin-4(1*H*)-one (**13**) was synthesized according to General procedure C starting from 2-aminobenzimidazole (**34**; 200 mg, 1.50 mmol, 1.0 equiv) and ethyl 2-ethyl-4,4-dimethyl-3-oxopentanoate (**37b**; 1.1 equiv). Yield: 7%, off-white solid. ^1^H-NMR (300 MHz, DMSO-*d*_6_) δ [ppm] = 12.78 (s, 1H), 8.45 (d, *J* = 8.0 Hz, 1H), 7.44 (m, 2H), 7.29 (ddd, *J* = 8.4, 5.6, 3.1 Hz, 1H), 2.76 (q, *J* = 7.1 Hz, 2H), 1.46 (s, 9H), 1.15 (t, *J* = 7.2 Hz, 3H). ^13^C-NMR (75 MHz, DMSO-*d*_6_) δ [ppm] = 160.5, 145.9, 126.2, 125.5, 121.4, 113.2, 110.8, 69.9, 30.5, 19.9, 13.9. HRMS (ESI): *m/z* [M+H]^+^ calculated for C_16_H_20_N_3_O: 270.16007, found: 270.1595.

*2-Cyclopropyl-3-ethylbenzo[4,5]imidazo[1,2-a]pyrimidin-4(1H)-one (****14****).* 2-Cyclopropyl-3-ethylbenzo[4,5]imidazo[1,2-*a*]pyrimidin-4(1*H*)-one (**14**) was synthesized according to General procedure C starting from 2-aminobenzimidazole (**34**; 146 mg, 1.10 mmol, 1.0 equiv) and ethyl 2-(cyclopropanecarbonyl)butanoate (**37c**; 1.1 equiv). Yield: 12%, white solid. ^1^H-NMR (300 MHz, DMSO-*d*_6_) δ [ppm] = 12.54 (br s, 1H), 8.44 (d, *J* = 7.9 Hz, 1H), 7.41 (m, 2H), 7.28 (m, 1H), 2.72 (q, *J* = 7.4 Hz, 2H), 2.17 (m, 1H), 1.12 (t, *J* = 7.4 Hz, 3H), 1.02 (m ,4H). ^13^C-NMR (75 MHz, pyridine-*d*_5_) δ [ppm] = 164.2, 160.9, 149.9, 132.7, 126.9, 122.5, 117.5, 114.3, 111.7, 71.8, 30.9, 19.6, 14.8, 10.2. HRMS (ESI): *m/z* [M+H]^+^ calculated for C_15_H_16_N_3_O: 254.12878, found: 254.1292.

*3-Ethyl-2-phenylbenzo[4,5]imidazo[1,2-a]pyrimidin-4(1H)-one (****15****).* 3-Ethyl-2-phenyl benzo[4,5]imidazo[1,2-*a*]pyrimidin-4(1*H*)-one (**15**) was synthesized according to General procedure C starting from 2-aminobenzimidazole (**34**; 232 mg, 1.75 mmol, 1.0 equiv) and ethyl 2-benzoylbutanoate (**37d**; 1.1 equiv). Yield: 7%, white solid. ^1^H-NMR (300 MHz, DMSO-*d*_6_) δ [ppm] = 8.51 (d, *J* = 8.1 Hz, 1H), 7.51 (m, 7H), 7.34 (m, 1H), 2.45 (q, *J* = 6.9 Hz, 2H), 1.09 (t, *J* = 7.1 Hz, 3H). ^13^C-NMR (75 MHz, pyridine-*d*_5_) δ [ppm] = 128.7, 128.3, 128.3, 126.2, 121.5, 115.7, 99.7, 19.7, 14.1. HRMS (ESI): *m/z* [M+H]^+^ calculated for C_18_H_16_N_3_O: 290.1288, found: 290.1292.

*3-Ethyl-2-(thiophen-2-yl)benzo[4,5]imidazo[1,2-a]pyrimidin-4(1H)-one (****16****).* 3-Ethyl-2-(thiophen-2-yl)benzo[4,5]imidazo[1,2-*a*]pyrimidin-4(1*H*)-one (**16**) was synthesized according to General procedure C starting from 2-aminobenzimidazole (**34**; 300 mg, 2.25 mmol, 1.0 equiv) and ethyl 2-(thiophene-2-carbonyl)butanoate (**37e**; 1.1 equiv). Yield: 13%, white solid. ^1^H-NMR (300 MHz, DMSO-*d*_6_) δ [ppm] = 12.88 (br s, 1H), 8.48 (d, *J* = 7.9 Hz, 1H), 7.78 (d, *J* = 5.2 Hz, 1H), 7.60 (d, *J* = 3.3 Hz, 1H), 7.45 (m, 2H), 7.33 (t, *J* = 7.5 Hz, 1H), 7.24 (m, 1H), 2.85 (t, *J* = 7.5 Hz, 2H), 1.25 (t, *J* = 7.5 Hz, 3H). ^13^C-NMR (75 MHz, pyridine-*d*_5_) δ [ppm] = 160.2, 146.9, 129.5, 128.3, 128.3, 126.4, 125.8, 121.7, 115.7, 112.0, 110.8, 19.3, 13.5. HRMS (ESI): *m/z* [M+H]^+^ calculated for C_16_H_14_N_3_OS: 296.0852, found: 296.0856.

*Ethyl 4-oxo-1,4-dihydrobenzo[4,5]imidazo[1,2-a]pyrimidine-3-carboxylate (****41****).* A mixture of 2-aminobenzimidazole (**34**; 500 mg, 3.756 mmol) and diethyl ethoxymethylenemalonate (**40**; 829 mg, 3.831 mmol) in dry MeOH was heated at reflux for 5 h. The mixture was cooled down in an ice-water bath and the resulting precipitate was collected by filtration. The crude product was purified by column chromatography on silica gel (2-20% gradient of MeOH in CH_2_Cl_2_, with 5% vol. addition of 7N ammonia in MeOH). Yield: 10%. ^1^H-NMR (300 MHz, DMSO-*d*_6_) δ [ppm] = 8.68 (s, 1H), 8.51 (d, *J* = 8.1 Hz, 1H), 7.58 (br d, *J* = 8.1 Hz, 1H), 7.53 (td, *J* = 7.2, 0.9 Hz, 1H), 7.39-7.45 (m, 1H), 4.25 (q, *J* = 7.2 Hz, 2H), 1.30 (t, *J* = 7.2 Hz, 3H). ^13^C-NMR (75 MHz, DMSO-*d*_6_) δ [ppm] = 164.3, 158.6, 156.3, 150.6, 131.6, 126.7, 126.3, 122.8, 116.1, 112.5, 103.4, 59.7, 14.3. HRMS (ESI): *m/z* [M+H]^+^ calculated for C_13_H_12_N_3_O_3_ : 258.0873, found: 258.0879.

*4-Oxo-1,4-dihydrobenzo[4,5]imidazo[1,2-a]pyrimidine-3-carboxylic acid (****42****).* A mixture of ethyl 4-oxo-1,4-dihydrobenzo[4,5]imidazo[1,2-*a*]pyrimidine-3-carboxylate (**41**; 99 mg, 0.385 mmol) and 0.2 M aqueous NaOH solution (5.4 ml) was heated at 85° C for 2.5 h. Next, the mixture was cooled in an ice-water bath and acidified using concentrated HCl. The precipitate was filtered-off, washed with H_2_O and dried affording **42** (54 mg, 0.236 mmol, 61%) as a pale-yellow solid. ^1^H-NMR (300 MHz, DMSO-*d*_6_) δ [ppm] = 13.19 (br s, 1H), 8.77 (s, 1H), 8.48 (d, *J* = 7.8 Hz, 1H), 7.66-7.57 (m, 2H), 7.53 (m, 1H), 7.48 (br t, *J* = 7.2 Hz, 1H). ^13^C-NMR (75 MHz, DMSO-*d*_6_) δ [ppm] = 165.0, 160.4, 159.1, 150.3, 131.1, 127.0, 126.0, 123.3, 116.2, 112.6, 102.5. HRMS (ESI): *m/z* [M-H]^-^ calculated for C_11_H_6_N_3_O_3_: 228.0414, found: 228.0417.

*Benzo[4,5]imidazo[1,2-a]pyrimidin-4(1H)-one (****5****).* A mixture of 4-oxo-1,4-dihydrobenzo[4,5]imidazo[1,2-*a*]pyrimidine-3-carboxylic acid *(***42***;* 47 mg, 0.205 mmol) and Cu powder (8 mg) in quinoline (0.8 ml) was heated at 200 °C for 1 h. The hot mixture was filtered through a paper filter and quinoline was removed by vacuum distillation. The residue was purified on silica gel column (2-4% gradient of MeOH in CH_2_Cl_2_). Fractions containing product were combined, concentrated and repurified using preparative TLC (3% of MeOH in CH_2_Cl_2_) affording **5** (6 mg, 0.032 mmol, 16%) as an off-white solid. ^1^H-NMR (300 MHz, DMSO-*d*_6_) δ [ppm] = 12.96 (br s, 1H), 8.43 (d, *J* = 8.1 Hz, 1H), 7.96 (d, *J* = 6.9 Hz, 1H), 7.55 (br d, *J* = 7.8 Hz, 1H), 7.47 (td, *J* = 7.8, 1.2 Hz, 1H), 7.32 (td, *J* = 8.1, 1.2 Hz, 1H), 5.96 (d, *J* = 6.9 Hz, 1H). ^13^C-NMR (75 MHz, DMSO-*d*_6_) δ [ppm] = 159.5, 149.0, 148.7, 134.7, 126.7, 125.8, 121.5, 115.4, 113.4, 100.1. HRMS (ESI): *m/z* [M+H]^+^ calculated for C_10_H_8_N_3_O: 186.0662, found: 186.0662.

*2-Methylbenzo[4,5]imidazo[1,2-a]pyrimidine-4(1H)-thione (****17****).* A mixture of 2-methylbenzo[4,5]imidazo[1,2-*a*]pyrimidin-4(1*H*)-one (**4**; 460 mg, 2.309 mmol) and P_2_S_5_ (513 mg, 2.309 mg) in pyridine (2.5 ml) was refluxed for 20 h. The solution was cooled to rt, 8 ml of H_2_O was added, and the resulting mixture was extracted with CH_2_Cl_2_ (3x). The organic layers were combined, dried over MgSO_4_, filtered and solvents were removed under reduced pressure. The residue was co-evaporated with toluene (3x) and the crude solid was purified by flash column chromatography on silica gel (40% of heptane in AcOEt) affording **17** (12 mg, 0.056 mmol, 2.5%) as a pale brown solid. ^1^H-NMR (300 MHz, DMSO-*d*_6_) δ [ppm] = 13.50 (br s, 1H), 9.73 (d, *J* = 8.4 Hz, 1H), 7.59-7.58 (m, 2H), 7.58-7.34 (m, 1H), 7.00 (s, 1H), 2.34 (s, 3H). HRMS (ESI): *m/z* [M+H]^+^ calculated for C_11_H_10_N_3_S: 216.0590, found: 216.059.

*2-Isopropylbenzo[4,5]imidazo[1,2-a]pyrimidine-4(1H)-thione (****18****).* A mixture of 2-*iso*propylbenzo[4,5]imidazo[1,2-*a*]pyrimidin-4(1*H*)-one (**7**; 300 mg, 1.320 mmol) and Lawesson's reagent (1.068 g, 2.640 mmol) in toluene (15 ml) was refluxed overnight. The mixture was cooled to rt, the precipitate was filtered-off, washed with toluene and dried. Purification using flash column chromatography on silica gel (0-3% gradient of MeOH in CH_2_Cl_2_) afforded **18** (256 mg, 1.052 mmol, 80%) as a pale-yellow solid. ^1^H-NMR (600 MHz, DMSO-*d*_6_) δ [ppm] = 9.72 (d, *J* = 8.4 Hz, 1H), 7.59-7.54 (m, 2H), 7.35 (ddd, *J* = 8.4, 7.2, 1.8 Hz, 1H), 6.99 (s, 1H), 2.88 (sept, *J* = 7.2 Hz, 1H), 1.25 (d, *J* = 7.2 Hz, 6H). ^13^C-NMR (150 MHz, DMSO-*d*_6_) δ [ppm] = 177.9, 166.9, 148.4, 131.5, 128.1, 127.5, 121.0, 118.3, 115.8, 111.4, 34.7, 21.6. HRMS (ESI): *m/z* [M+H]^+^ calculated for C_13_H_14_N_3_S: 244.0903, found: 244.0889.

*2-Isopropyl-4-(methylthio)benzo[4,5]imidazo[1,2-a]pyrimidine (****19****).* To a mixture of 2-*iso*propylbenzo[4,5]imidazo[1,2-*a*]pyrimidine-4(1*H*)-thione (**18**; 100 mg, 0.411 mmol) and NaHCO_3_ (43 mg, 0.512 mmol) in acetone (2 ml) was added Me_2_SO_4_ (78 µl, 0.822 mmol) and the resulting mixture was refluxed for 20 h. Volatiles were removed under reduced pressure and the crude product was purified first on silica gel column chromatography (0-10% gradient of MeOH in CH_2_Cl_2_) and second on preparative TLC (4% of MeOH in CH_2_Cl_2_) affording **19** (17 mg, 0.066 mmol, 16%). ^1^H-NMR (300 MHz, CDCl_3_) δ [ppm] = 8.35 (d, *J* = 8.4 Hz, 1H), 7.95 (d, *J* = 8.1 Hz, 1H), 7.52 (t, *J* = 7.5 Hz, 1H), 7.33 (t, *J* = 7.5 Hz, 1H), 6.46 (s, 1H), 3.11 (sept, *J* = 6.9 Hz, 1H), 2.74 (s, 3H), 1.39 (d, *J* = 6.9 Hz, 6H). ^13^C-NMR (75 MHz, CDCl_3_) δ [ppm] = 171.5, 152.9, 151.7, 144.8, 128.6, 125.8, 121.0, 119.9, 115.5, 100.5, 37.3, 21.8, 14.9. HRMS (ESI): *m/z* [M+H]^+^ calculated for C_14_H_16_N_3_S: 258.1059, found: 258.1064.

*2-Isopropyl-10-methylbenzo[4,5]imidazo[1,2-a]pyrimidin-4(10H)-one (****21****).* 2-*Iso*propyl-10-methylbenzo[4,5]imidazo[1,2-*a*]pyrimidin-4(10*H*)-one (**21**) was synthesized according to General procedure C starting from 1-methyl-1*H*-benzo[*d*]imidazol-2-amine (**43**; 50 mg, 0.340 mmol) and ethyl 4-methyl-3-oxopentanoate (**35c**; 2.0 equiv). The crude product was purified by column chromatography on silica gel (0-4% gradient of MeOH in CH_2_Cl_2_). Yield: 12%, off-white solid. ^1^H-NMR (300 MHz, CDCl_3_) δ [ppm] = 7.63 (d, *J* = 7.8 Hz, 1H), 7.46 (td, *J* = 7.8, 0.9 Hz, 1H), 7.36-7.29 (m, 2H), 3.80 (s, 3H), 2.86 (sept, *J* = 6.9 Hz, 1H), 1.30 (d, *J* = 6.9 Hz, 6H). ^13^C-NMR (75 MHz, CDCl_3_) δ [ppm] = 173.7, 161.1, 149.0, 131.7, 126.1, 125.7, 122.5, 116.9, 108.3, 99.0, 36.3, 28.3, 21.8. HRMS (ESI): *m/z* [M+H]^+^ calculated for C_14_H_16_N_3_O: 242.1288, found: 242.1290.

*2-Methyl-4H-benzo[4,5]oxazolo[3,2-a]pyrimidin-4-one (****22****).* A mixture of 2-aminobenzoxazole (**44a**; 200 mg, 1.491 mmol) and ethyl acetoacetate (**35a**; 1.1 equiv) in polyphosphoric acid (PPA; 1.7 g) was heated at 120 °C overnight. After cooling the mixture in an ice-water bath, ice cold H_2_O was added to the flask and the mixture was neutralized using a 10M aqueous NaOH solution. Precipitate that formed was filtered-off, washed with H_2_O and dried. The crude product was suspended in MeOH, the solid was filtered-off and dried affording **22** (41 mg, 0.205 mmol, 14%) as a pale-yellow solid. ^1^H-NMR (300 MHz, CDCl_3_) δ [ppm] = 8.41-8.38 (m, 1H), 7.55-7.42 (m, 3H), 6.24 (s, 1H), 2.42 (s, 3H). ^13^C-NMR (75 MHz, CDCl_3_) δ [ppm] = 164.9, 159.5, 155.3, 144.7, 127.1, 126.3, 125.4, 116.7, 111.1, 106.4, 24.5. HRMS (ESI): *m/z* [M+H]^+^ calculated for C_11_H_9_N_2_O_2_: 201.0658, found: 201.0662.

*2-Methyl-4H-benzo[4,5]thiazolo[3,2-a]pyrimidin-4-one (****23****).* Mixture of 2-aminobenzothiazole (**44b**; 200 mg, 1.332 mmol) and ethyl acetoacetate (**35a**; 1.1 equiv) in polyphosphoric acid (PPA; 1.5 g) was heated at 120 °C overnight. After cooling the mixture in an ice-water bath, ice cold H_2_O was added to the flask and the mixture was neutralized using a 10M aqueous NaOH solution. Precipitate that formed was filtered-off, washed with H_2_O and dried. The crude product was suspended in MeOH, the solid was filtered-off and dried affording **23** (90 mg, 0.416 mmol, 31%) as a pale-yellow solid. ^1^H-NMR (300 MHz, CDCl_3_) δ [ppm] = 9.08-9.05 (m, 1H), 7.68-7.65 (m, 1H), 7.54-7.45 (m, 2H), 6.26 (s, 1H), 2.39 (s, 3H). ^13^C-NMR (75 MHz, CDCl_3_) δ [ppm] = 163.0, 161.6, 161.3, 136.3, 127.1, 127.1, 124.2, 121.9, 120.2, 107.3, 23.8. HRMS (ESI): *m/z* [M+H]^+^ calculated for C_11_H_9_N_2_OS: 217.0430, found: 217.0436.

*5,6-Difluoro-1H-benzo[d]imidazol-2-amine (****46a****).* 5,6-Difluoro-1*H*-benzo[*d*]imidazol-2-amine (**46a**) was synthesized from 4,5-difluorobenzene-1,2-diamine (**45a**; 500 mg, 3.47 mmol) according to General procedure E. The crude residue was purified by silica gel flash column chromatography (4-6% gradient of MeOH in CH_2_Cl_2_ with 2% vol. addition of 7N ammonia in MeOH). Yield: 78%, pale brown solid. ^1^H-NMR (600 MHz, DMSO-*d*_6_) δ [ppm] = 7.05-7.08 (m, 2H), 6.31 (br, 2H). ^13^C-NMR (150 MHz, DMSO-*d*_6_) δ [ppm] = 157.0, 144.6 (dd, *J* = 232.4 Hz, *J* = 15.1 Hz), 134.3 (br s), 99.6. ^19^F-NMR (470 MHz, DMSO-*d*_6_) δ [ppm] = ‑150.2. HRMS (ESI): *m/z* [M+H]^+^ calculated for C_7_H_6_F_2_N_3_: 170.0524, found: 170.0531.

*5,6-Dichloro-1H-benzo[d]imidazol-2-amine (****46b****).* 5,6-Dichloro-1*H*-benzo[*d*]imidazol-2-amine (**46b**) was synthesized from 4,5-dichlorobenzene-1,2-diamine (**45b**; 500 mg, 2.82 mmol) according to General procedure E. Yield: 99%, pale yellow solid. ^1^H-NMR (300 MHz, DMSO-*d*_6_) δ [ppm] = 7.27 (s, 2H), 6.58 (s, 2H). ^13^C-NMR (75 MHz, DMSO-*d*_6_) δ [ppm] = 157.1, 138.8, 120.8, 112.4. HRMS (ESI): *m/z* [M+H]^+^ calculated for C_7_H_6_Cl_2_N_3_: 201.9933, found: 201.9934.

*5,6-Dibromo-1H-benzo[d]imidazol-2-amine (****46c****).* 5,6-Dibromo-1*H*-benzo[*d*]imidazol-2-amine (**46c**) was synthesized from 4,5-dibromo-1,2-diaminobenzene (**45c**; 100 mg, 0.376 mmol) according to General procedure E. The crude residue was purified by silica gel flash column chromatography (3-10% gradient of MeOH in CH_2_Cl_2_). Yield: 91%, white solid. ^1^H-NMR (300 MHz, DMSO-*d*_6_) δ [ppm] = 7.46 (s, 2H), 6.93 (br s, 2H).

*5,6-Dimethyl-1H-benzo[d]imidazol-2-amine hydrobromide (****46d****).* 5,6-Dimethyl-1*H*-benzo[*d*]imidazol-2-amine hydrobromide (**46d**) was synthesized from 4,5-dimethylbenzene-1,2-diamine (**45d**) according to General procedure E. After addition of concentrated aqueous NH_3_ solution, the product was still present in the aqueous phase. The water layer was concentrated, and the solid residue was purified on silica gel column (20% of MeOH in CH_2_Cl_2_ with 2% (v/v) addition of 7 N ammonia in MeOH). Yield: 76%, pale yellow solid. ^1^H-NMR (300 MHz, DMSO-*d*_6_) δ [ppm] = 7.97 (br s, 2H), 7.11 (s, 2H), 2.26 (s, 6H). ^13^C-NMR (75 MHz, DMSO-*d*_6_) δ [ppm] = 150.7, 130.6, 129.1, 111.9, 19.6. HRMS (ESI): *m/z* [M+H]^+^ calculated for C_9_H_12_N_3_: 162.1026, found: 162.1033.

*4-Methyl-1H-benzo[d]imidazol-2-amine (****46e****).* 4-Methyl-1*H*-benzo[*d*]imidazol-2-amine (**46e**) was synthesized from 3-methylbenzene-1,2-diamine (**45e**; 500 mg, 4.09 mmol) according to General procedure E. The crude residue was purified by silica gel flash column chromatography (4-5% of MeOH in CH_2_Cl_2_ with 2% vol. addition of 7 N ammonia in MeOH). Yield: 54%, pale orange solid. ^1^H-NMR (300 MHz, DMSO-*d*_6_) δ [ppm] = 6.93 (d, *J* = 7.5 Hz, 1H), 6.75 (t, *J* = 7.5 Hz, 1H), 6.67 (d, *J* = 7.2 Hz, 1H), 6.08 (br, 2H), 2.34 (s, 3H). ^13^C-NMR (75 MHz, DMSO-*d*_6_) δ [ppm] = 154.8, 138.4, 137.2, 121.2, 120.1, 118.8, 108.8, 16.6. HRMS (ESI): *m/z* [M+H]^+^ calculated for C_8_H_10_N_3_: 148.0869, found: 148.0874.

*3-Ethyl-7,8-difluoro-2-isopropylbenzo[4,5]imidazo[1,2-a]pyrimidin-4(1H)-one (****24****).* 3-Ethyl-7,8-difluoro-2-*iso*propylbenzo[4,5]imidazo[1,2-*a*]pyrimidin-4(1*H*)-one (**24**) was synthesized according to General procedure C starting from 5,6-difluoro-1*H*-benzo[*d*]imidazol-2-amine (**46a**; 150 mg, 0.886 mmol) and ethyl 2-ethyl-4-methyl-3-oxopentanoate (**36c**; 1.1 equiv). MeOH was added, the precipitate was filtered-off, washed with MeOH and dried. Yield: 57%, pale yellow solid. ^1^H-NMR (500 MHz, DMSO-*d*_6_) δ [ppm] = 14.36 and 12.93 (2 x br s, 1H), 8.26 (br s, 1H), 7.54 (br s, 1H), 3.23 (br s, 1H), 2.56 (q, J = 7.5 Hz, 2H), 1.25 (br s, 6H), 1.05 (t, *J* = 7.5 Hz, 3H). ^19^F-NMR (470 MHz, DMSO-*d*_6_) δ [ppm] = -140.7, -145.5, -145.1, -138.9. HRMS (ESI): *m/z* [M+H]^+^ calculated for C_15_H_16_F_2_N_3_O: 292.1256, found: 292.1253.

*7,8-Dichloro-3-ethyl-2-isopropylbenzo[4,5]imidazo[1,2-a]pyrimidin-4(1H)-one (****25****)****.*** 7,8-Dichloro-3-ethyl-2-*iso*propylbenzo[4,5]imidazo[1,2-*a*]pyrimidin-4(1*H*)-one (**25**) was synthesized according to General procedure C starting from 5,6-dichloro-1*H*-benzo[*d*]imidazol-2-amine (**46b**; 150 mg, 0.742 mmol) and ethyl 2-ethyl-4-methyl-3-oxopentanoate (**36c**; 1.1 equiv). Volatiles were removed under reduced pressure and the crude product was purified by silica gel column chromatography (0-25 % gradient of EtOAc in cyclohexane). Yield: 20%, beige solid. ^1^H-NMR (300 MHz, DMSO-*d*_6_) δ [ppm] = 12.71 (br s, 1H), 8.45 (s, 1H), 7.70 (s, 1H), 3.25 (sept, *J* = 6.6 Hz, 1H), 2.57 (q, *J* = 7.2 Hz, 2H), 1.27 (d, *J* = 6.6 Hz, 6H), 1.07 (t, *J* = 7.2 Hz, 3H). ^13^C-NMR (75 MHz, DMSO-*d*_6_) δ [ppm] = 159.3, 148.2, 127.7, 126.5, 122.6, 115.9, 29.8, 20.8,17.6, 14.4. HRMS (ESI): *m/z* [M+H]^+^ calculated for C_15_H_16_Cl_2_N_3_O: 324.0665, found: 324.0668.

*7,8-Dibromo-3-ethyl-2-isopropylbenzo[4,5]imidazo[1,2-a]pyrimidin-4(1H)-one (****26****).* To a solution of **46c** (100 mg, 0.343 mmol) in pyridine (2 ml) was added ethyl 2-ethyl-4-methyl-3-oxopentanoate (**36c**; 1.0 equiv). The reaction mixture was heated to reflux and stirred for 48 h. After reaction stagnation, the solvent was evaporated, and the crude residue was purified by silica gel flash chromatography (heptane/EtOAc in a ratio of 5:1 as eluent). Yield: 22%. ^1^H-NMR (300 MHz, CDCl_3_) δ [ppm] = 8.84 (s, 1H), 7.85 (s, 1H), 3.34 (m, *J* = 6.9 Hz, 1H), 2.68 (q, *J* = 7.4 Hz, 2H), 1.35 (d, *J* = 6.9 Hz, 6H), 1.19 (t, *J* = 7.4 Hz, 3H). ^13^C-NMR (75 MHz, pyridine-*d*_5_) δ [ppm] = 162.4, 160.1, 137.1, 120.6, 120.0, 118.2, 115.1, 111.2, 61.5, 54.9, 29.8, 21.2, 18.4, 14.7. HRMS (ESI): *m/z* [M+H]^+^ calculated for C_15_H_16_Br_2_N_3_O: 411.9656, found: 411.9651.

*3-Ethyl-2-isopropyl-7,8-dimethylbenzo[4,5]imidazo[1,2-a]pyrimidin-4(1H)-one (****27****).* 3-Ethyl-2-*iso*propyl-7,8-dimethylbenzo[4,5]imidazo[1,2-*a*]pyrimidin-4(1*H*)-one (**27**) was synthesized according to General procedure C starting from 5,6-dimethyl-1*H*-benzo[*d*]imidazol-2-amine hydrobromide (**46d**; 333 mg, 1.375 mmol) and ethyl 2-ethyl-4-methyl-3-oxopentanoate (**36c**; 1.64 equiv). MeOH was added and the precipitate was filtered-off from the reaction mixture, washed with MeOH and dried. Yield: 36%, beige solid. ^1^H-NMR (300 MHz, DMSO-*d*_6_) δ [ppm] = 12.52 (br s, 1H), 8.22 (s, 1H), 7.18 (s, 1H), 3.21 (sept, *J* = 6.6 Hz, 1H), 2.59 (q, *J* = 7.5 Hz, 2H), 2.33 (s, 6H), 1.21 (d, *J* = 6.6 Hz, 6H), 1.07 (t, *J* = 7.5 Hz, 3H). ^13^C-NMR (75 MHz, pyridine-*d*_5_) δ [ppm] = 166.9, 161.1, 149.2, 135.1, 131.1, 130.3, 126.1, 117.5, 112.5, 112.2, 31.8, 22.3, 20.6, 20.3, 19.1, 15.4. HRMS (ESI): *m/z* [M+H]^+^ calculated for C_17_H_22_N_3_O: 284.1757, found: 284.1753.

*3-Ethyl-2-isopropyl-9-methylbenzo[4,5]imidazo[1,2-a]pyrimidin-4(1H)-one (****31****).* 3-Ethyl-2-*iso*propyl-9-methylbenzo[4,5]imidazo[1,2-*a*]pyrimidin-4(1*H*)-one (**31**) was synthesized according to General procedure C starting from 4-methyl-1*H*-benzo[*d*]imidazol-2-amine (**46e**; 150 mg, 1.02 mmol) and ethyl 2-ethyl-4-methyl-3-oxopentanoate (**36c**; 1.0 equiv). Volatiles were removed under reduced pressure and the residue was suspended in MeOH. The solid was filtered-off and dried. The filtrate was concentrated to dryness and the procedure was repeated. All combined solids were purified by silica gel column chromatography (25% EtOAc in cyclohexane). Yield: 32% (single isomer), off-white solid. ^1^H-NMR (300 MHz, DMSO-*d*_6_) δ [ppm] = 12.78 (br s, 1H), 8.26 (d, *J* = 7.5 Hz, 1H), 7.23 (br d, *J* = 6.9 Hz, 1H), 7.17 (t, *J* = 7.8 Hz, 1H), 3.26-3.19 (m, 1H), 2.60 (q, *J* = 7.5 Hz, 2H), 2.48 (s, 3H), 1.24 (d, *J* = 6.6 Hz, 6H), 1.08 (t, *J* = 7.5 Hz, 3H). ^13^C-NMR (75 MHz, DMSO-*d*_6_) δ [ppm] = 167.1, 159.6, 147.6, 129.6, 126.5, 125.5, 121.2, 120.7, 112.8, 111.3, 30.6, 21.6, 17.7, 16.3, 14.5. HRMS (ESI): *m/z* [M+H]^+^ calculated for C_16_H_20_N_3_O: 270.1601, found: 270.1594.

*N-Benzyl-4-methyl-2-nitroaniline (****48a****).* The title compound was obtained according to General procedure F starting from 4-methyl-2-nitroaniline (**47a**; 1 g, 6.57 mmol) and benzyl bromide (940 μl, 7.89 mmol, 1.2 equiv). Yield: 81%, orange solid. ^1^H-NMR (300 MHz, CDCl_3_) δ [ppm] = 8.33 (br s, 1H), 7.99 (d, *J* = 2.5 Hz, 1H), 7.38-7.25 (m, 5H), 7.20 (dd, *J* = 7.5 Hz, *J* = 2.5 Hz, 1H), 6.73 (d, *J* = 7.5 Hz, 1H), 4.53 (d, *J* = 5 Hz, 2H), 2.25 (s, 3H). ^13^C-NMR (75 MHz, CDCl_3_) δ [ppm] = 142.6, 136.8, 136.8, 131.0, 128.0, 126.7, 126.1, 125.2, 124.4, 113.3, 46.3, 19.1. HRMS (ESI): *m/z* [M+H]^+^ calculated for C_14_H_15_N_2_O_2_: 265.0948, found 265.0950.

*N-Benzyl-5-methyl-2-nitroaniline (****48b****).* The title compound was obtained according to General procedure F starting from 5-methyl-2-nitroaniline (**47b**; 1 g, 6.57 mmol) and benzyl bromide (940 μl, 7.89 mmol, 1.2 equiv). Yield: 68%, orange solid. ^1^H-NMR (600 MHz, DMSO-*d*_6_) δ [ppm] = 8.64 (br s, 1H), 7.98 (d, *J* = 8.7 Hz, 1H), 6.78 (s, 1H), 7.32 (m, 5H), 6.54 (d, *J* = 10.3 Hz, 1H), 4.63 (s, 2H), 2.23 (s, 3H).

*N-Benzyl-4-fluoro-2-nitroaniline (****48c****).* The title compound was obtained according to General procedure F starting from 4-fluoro-2-nitroaniline (**47c**; 2 g, 12.81 mmol) and benzyl bromide (2.28 ml, 19.22 mmol, 1.5 equiv). Yield: 76%, orange solid. ^1^H-NMR (300 MHz, DMSO-*d*_6_) δ [ppm] = 8.65 (br s, 1H), 7.87 (m, 1H), 7.30 (m, 7H), 6.95 (m, 1H), 4.63 (d, *J* = 6.1 Hz, 2H).

*N-Benzyl-5-fluoro-2-nitroaniline (****48d****).* The title compound was obtained according to General procedure F starting from 5-fluoro-2-nitroaniline (**47d**; 500 mg, 3.20 mmol) and benzyl bromide (570 μl, 4.80 mmol, 1.2 equiv). Yield: 53%, orange solid. ^1^H-NMR (300 MHz, DMSO-*d*_6_) δ [ppm] = 8.82 (br s, 1H), 8.19 (m, 1H), 7.33 (m, 5H), 6.71 (dd, *J* = 12.3, 2.6 Hz, 1H), 6.54 (m, 1H), 4.63 (d, *J* = 6.1 Hz, 2H).

*N^1^-Benzyl-4-methylbenzene-1,2-diamine (****49a****).* The title compound was obtained according to General procedure G as a colorless oil. ^1^H-NMR (300 MHz, CDCl_3_) δ [ppm] = 7.34-7.22 (m, 5H), 6.58-6.50 (m, 3H), 4.23 (s, 2H), 3.36 (br s, 3H), 2.19 (s, 3H).

*N^1^-Benzyl-5-methylbenzene-1,2-diamine (****49b****).* The title compound was obtained according to General procedure G as a colorless oil. ^1^H-NMR (300 MHz, DMSO-*d*_6_) δ [ppm] = 7.32 (m, 6H), 6.45 (d, *J* = 7.6 Hz, 1H), 6.21 (d, *J* = 7.2 Hz, 2H), 4.99 (s, 1H), 4.26 (s, 4H), 2.04 (s, 3H).

*N^1^-Benzyl-4-fluorobenzene-1,2-diamine (****49c****).* The title compound was obtained according to General procedure G as a dark green oil. The product was immediately used in the next reaction due to rapid decomposition after exposure to air.

*N^1^-Benzyl-5-fluorobenzene-1,2-diamine (****49d****).* The title compound was obtained according to General procedure G as a dark red oil. The product was immediately used in the next reaction due to rapid decomposition after exposure to air.

*1-Benzyl-5-methyl-1H-benzo[d]imidazol-2-amine (****50a****).* The title product was synthesized from **49a** (500 mg, 2.36 mmol) according to General procedure E affording the title compound as a white solid (294 mg, 52%). ^1^H-NMR (300 MHz, DMSO-*d*_6_) δ [ppm] = 7.22 (m, 5H), 6.92 (d, *J* = 18.3 Hz, 2H), 6.63 (d, *J* = 7.8 Hz, 1H), 6.46 (s, 2H), 5.22 (s, 2H), 2.29 (s, 3H).

*1-Benzyl-6-methyl-1H-benzo[d]imidazol-2-amine (****50b****).* The title product was synthesized from **49b** (400 mg, 1.88 mmol) according to General procedure E affording the title compound as a white solid (220 mg, 49%). ^1^H-NMR (300 MHz, DMSO-*d*_6_) δ [ppm] = 7.26 (m, 5H), 7.02 (d, *J* = 8.3 Hz, 1H), 6.85 (s, 1H), 6.74 (d, *J* = 7.9 Hz, 1H), 6.40 (s, 2H), 5.22 (s, 2H), 2.27 (s, 3H).

*1-Benzyl-5-fluoro-1H-benzo[d]imidazol-2-amine (****50c****).* The title product was synthesized from **49c** (870 mg, 1.71 mmol) according to General procedure E affording the title compound as a purple solid (556 mg, mmol, 57%). ^1^H-NMR (300 MHz, DMSO-*d*_6_) δ [ppm] = 7.26 (m, 5H), 6.98 (m, 1H), 6.90 (m, 1H), 6.95 (m, 3H), 5.25 (s, 2H).

*1-Benzyl-6-fluoro-1H-benzo[d]imidazol-2-amine (****50d****).* The title product was synthesized from **49d** (370 mg, 1.71 mmol) according to General procedure E affording the title compound as a purple solid (251 mg, 61%). ^1^H-NMR (300 MHz, DMSO-*d*_6_) δ [ppm] = 7.27 (m, 5H), 7.08 (dd, *J* = 8.5, 4.9 Hz, 1H), 6.97 (dd, *J* = 9.4, 2.4 Hz, 1H), 6.73 (m, 1H), 6.57 (s, 2H), 5.25 (s, 2H).

*10-Benzyl-3-ethyl-2-isopropyl-7-methylbenzo[4,5]imidazo[1,2-a]pyrimidin-4(10H)-one (****51a****).* The title compound was synthesized starting from **50a** (200 mg, 0.842 mmol) and ethyl 2-ethyl-4-methyl-3-oxopentanoate (**36c**) (1.1 equiv) according to General procedure C. After removal of the volatiles, the crude residue was purified by silica gel flash column chromatography (using 10% of EtOAc in heptane as mobile phase) affording the title product as an off-white solid (96 mg, 32%). ^1^H-NMR (300 MHz, DMSO-*d*_6_) δ [ppm] = 8.45 (m, 1H), 7.69 (d, *J* = 9.3 Hz, 1H), 7.50 (m, 2H), 7.31 (m, 3H), 7.17 (t, *J* = 9.1 Hz, 1H), 5.46 (s, 2H), 3.23 (m, 1H), 2.62 (m, 2H), 1.24 (d, *J* = 6.6 Hz, 6H), 1.08 (t, *J* = 7.4 Hz, 3H).

*10-Benzyl-3-ethyl-2-isopropyl-8-methylbenzo[4,5]imidazo[1,2-a]pyrimidin-4(10H)-one (****51b****).* The title compound was synthesized starting from **50b** (260 mg, 1.10 mmol) and ethyl 2-ethyl-4-methyl-3-oxopentanoate (**36c**) (1.1 equiv) according to General procedure C. After removal of the volatiles, the crude product was purified by silica gel flash chromatography (using 10% of EtOAc in heptane as mobile phase) affording the title compound as an off-white solid (183 mg, 46%). ^1^H-NMR (300 MHz, DMSO-*d*_6_) δ [ppm] = 8.49 (d, *J* = 8.1 Hz, 1H), 7.02-7.44 (m, 7H), 5.39 (s, 2H), 3.25 (m, 1H), 2.73 (q, *J* = 7.5 Hz, 2H), 2.45 (s, 3H), 1.29 (d, *J* = 6.6 Hz, 6H), 1.19 (t, *J* = 7.5 Hz, 3H).

*10-Benzyl-3-ethyl-2-isopropyl-8-fluorobenzo[4,5]imidazo[1,2-a]pyrimidin-4(10H)-one (****51d****).* The title compound was synthesized starting from **50d** (250 mg, 1.04 mmol) and ethyl 2-ethyl-4-methyl-3-oxopentanoate (**36c**) (1.1 equiv) according to General procedure C. After removal of the volatiles, the crude product was purified by silica gel flash chromatography (using 10% of EtOAc in heptane as mobile phase) affording the title compound as a brown solid (103 mg, 27%). ^1^H-NMR (300 MHz, DMSO-*d*_6_) δ [ppm] = 8.49 (d, *J* = 8.1 Hz, 1H), 7.02-7.44 (m, 7H), 5.39 (s, 2H), 3.25 (m, 1H), 2.73 (q, *J* = 7.5 Hz, 2H), 2.45 (s, 3H), 1.29 (d, *J* = 6.6 Hz, 6H), 1.19 (t, *J* = 7.5 Hz, 3H).

*3-Ethyl-2-isopropyl-7-methylbenzo[4,5]imidazo[1,2-a]pyrimidin-4(1H)-one (****28****).* The title compound was obtained according to General procedure H starting from **51a** (50 mg, 0.139 mmol). Yield: 58%, white solid. ^1^H-NMR (300 MHz, DMSO-*d*_6_) δ [ppm] = 12.57 (br s, 1H), 8.28 (s, 1H), 7.28 (m, 2H), 3.21 (m, 1H), 2.60 (m, 2H), 2.45 (s, 3H), 1.22 (d, *J* = 6.7 Hz, 6H), 1.07 (t, *J* = 7.4 Hz, 3H).^13^C-NMR (75 MHz, DMSO-*d*_6_) δ [ppm] = 159.8, 147.8, 130.6, 126.9, 126.3, 115.7, 110.9, 30.7, 21.7, 21.3, 17.9, 14.7. HRMS (ESI): *m/z* [M+H]^+^ calculated for C_16_H_20_N_3_O: 270.1601, found: 270.1606.

*3-Ethyl-2-isopropyl-8-methylbenzo[4,5]imidazo[1,2-a]pyrimidin-4(1H)-one (****29****).* The title compound was obtained according to General procedure H starting from **51b** (183 mg, 0.509 mmol). Yield: 54%, white solid. ^1^H-NMR (300 MHz, DMSO-*d*_6_) δ [ppm] = 12.53 (br s, 1H), 8.29 (d, *J* = 8.1 Hz, 1H), 7.21 (s, 1H), 7.09 (d, *J* = 8.2 Hz, 1H), 3.19 (m, 1H), 2.57 (q, *J* = 6.9 Hz, 2H), 2.44 (s, 3H), 1.21 (d, *J* = 6.2 Hz, 6H), 1.07 (t, *J* = 7.0 Hz, 3H). ^13^C-NMR (75 MHz, DMSO-*d*_6_) δ [ppm] = 159.6, 147.8, 135.7, 122.3, 115.2, 111.3, 30.7, 21.7, 21.4, 17.9, 14.7. HRMS (ESI): *m/z* [M+H]^+^ calculated for C_16_H_20_N_3_O: 270.1601, found: 270.1592.

*3-Ethyl-2-isopropyl-8-fluorobenzo[4,5]imidazo[1,2-a]pyrimidin-4(1H)-one (****30****).* The title compound was obtained according to General procedure H starting from **51d** (103 mg, 0.283 mmol). Yield: 40%, white solid. ^1^H-NMR (300 MHz, pyridine-*d*_5_) δ [ppm] = 8.29 (d, *J* = 8.1 Hz, 1H), 8.81 (dd, *J* = 8.8, 5.1 Hz, 1H), 7.38 (dd, *J* = 9.0, 2.3 Hz, 1H), 7.09 (m, 1H), 3.20 (m, 2H), 2.82 (q, *J* = 7.3 Hz, 2H), 1.35 (d, *J* = 6.7 Hz, 6H), 1.27 (t, *J* = 7.4 Hz, 3H). ^13^C-NMR (75 MHz, DMSO-*d*_6_) δ [ppm] = 159.6, 147.8, 135.7, 122.3, 115.2, 111.3, 30.7, 21.7, 21.4, 17.9, 14.7. HRMS (ESI): *m/z* [M+H]^+^ calculated for C_15_H_17_N_3_OF: 274.1350, found: 274.1352.

*Propyl (6-fluoropyridin-3-yl)carbamate (****53)****.* 6-Fluoropyridine-3-amine (**52**, 5 g, 44.60 mmol, 1.0 equiv) and NaHCO_3_ (11.24 g, 133.80 mmol, 3.0 equiv) were dissolved in dry THF (25 ml) and propyl chloroformate (16.40 g, 133.80 mmol, 3.0 equiv) was added slowly. The reaction was completed after 30 min (TLC: 20% ethyl acetate in petroleum ether) and the solvent evaporated under reduced pressure. Water was added and extracted with ethyl acetate (3 x 10 mL). The combined organic phases were washed with brine, dried over MgSO_4_, filtered and the solvent removed under reduced pressure. The crude product was purified by flash column chromatography (10% ethyl acetate in petroleum ether) to yield **53** (7.15 g, 81%) as a yellowish solid. ^1^H NMR (600 MHz, DMSO-*d*_6_) δ [ppm] = 9.87 (s, 1H), 8.31 - 8.20 (m, 1H), 8.07 - 7.95 (m, 1H), 7.12 (dd, *J* = 8.9, 3.2 Hz, 1H), 4.05 (t, *J* = 6.7 Hz, 2H), 1.64 (sext, *J* = 7.2 Hz, 2H,), 0.92 (t, *J* = 7.4 Hz, 3H). ^13^C NMR (151 MHz, DMSO-*d*_6_) δ [ppm] = 158.2 (d, *J* = 231.4 Hz), 153.8, 136.6 (d, *J* = 15.2 Hz,), 134.3 (d, *J* = 4.4 Hz), 131.6, 109.3 (d, *J* = 39.5 Hz), 66.1, 21.8, 10.2. LC-MS (m/z): 198.7 [M+H]^+^. Purity (HPLC-UV 254 nm-ESI-MS): 97.1%.

*Propyl (6-fluoro-2-nitropyridin-3-yl)carbamate (****54****).* Propyl (6-fluoropyridin-3-yl)carbamate (**53**, 2.0 g, 10.09 mmol, 1.0 equiv) was dissolved in conc. HNO_3_ (2 mL) and the solution was cooled to 0 °C. Concentrated H_2_SO_4_ (2 mL) was added dropwise over 30 min and the solution was stirred for further 5 h at rt. After complete conversion (TLC: 20% ethyl acetate in petroleum ether) the mixture was basified by the addition of 2N aqueous NaOH solution, extracted with ethyl acetate (3x) and the combined organic phases dried over MgSO_4_. The solvent was removed under reduced pressure and the crude product purified by flash column chromatography (50% ethyl acetate in petroleum ether) to yield **54** (1.996 g, 81%) as a yellowish solid. ^1^H NMR (600 MHz, DMSO-*d*_6_) δ [ppm] = 10.02 (s, 1H), 8.35 (dd, *J* = 8.7, 6.5 Hz, 1H), 7.69 (dd, *J* = 8.7, 3.3 Hz, 1H), 4.06 (t, *J* = 6.7 Hz, 2H), 1.63 (sext, *J* = 7.4, 2H), 0.92 (t, *J* = 7.4 Hz, 3H). ^13^C NMR (151 MHz, DMSO-*d*_6_) δ [ppm] = 155.5 (d, *J* = 231.4 Hz), 153.5, 144.8 (d, *J* = 12.7 Hz), 141.0 (d, *J* = 7.6 Hz,), 126.3 (d, *J* = 4.0 Hz,), 116.4 (d, *J* = 37.8 Hz), 67.0, 21.7, 10.1. LC-MS (m/z): 243.9 [M+H]^+^. Purity (HPLC-UV 254 nm-ESI-MS): 73.3%.

*6-Fluoro-2-nitropyridin-3-amine (****55****).* Propyl (6-fluoro-2-nitropyridin-3-yl)carbamate (**54**, 1.0 equiv) was dissolved in acetonitrile (10 mL). 2n NaOH solution was added in portions over 8 h until complete conversion of the starting material (TLC: CH_2_Cl_2_). Acetonitrile was removed under reduced pressure and the residue neutralized with saturated aqueous NH_4_Cl solution (10 mL), extracted with ethyl acetate (3 x 5 mL) and the combined organic phases were washed with brine, dried over MgSO_4_ and the solvent removed under reduced pressure. The crude product was purified by automated flash column chromatography (0-60% ethyl acetate in petroleum ether) to yield **55** (445 mg, 48%) as a yellowish solid. ^1^H NMR (600 MHz, DMSO-*d*_6_) δ [ppm] = 7.73 (dd, *J* = 8.9, 6.8 Hz, 1H), 7.49 (s, 2H), 7.41 (dd, *J* = 8.9, 3.7 Hz, 1H). ^13^C NMR (151 MHz, DMSO-*d*_6_) δ [ppm] = 149.6 (d, *J* = 234.0 Hz), 140.4 (d, *J* = 1.5 Hz), 135.9 (d, *J* = 6.6 Hz), 132.8 (d, *J* = 13.0 Hz), 119.1 (d, *J* = 42.1 Hz). LCMS (m/z): 157.8 [M+H]^+^. Purity (HPLC-UV 254 nm-ESI-MS): 97.5%.

*6-Fluoropyridine-2,3-diamine (****56b****).* To a solution of 6-fluoro-2-nitropyridin-3-amine (**55**, 352 mg, 2.241 mmol, 1.0 equiv) in MeOH (5 mL), were added 10% palladium on charcoal (238 mg, 1.0 equiv). The reaction mixture was stirred for 1 h under hydrogen atmosphere (1 atm.) at rt. After complete conversion of the starting material monitored by TLC (10% MeOH in CH_2_Cl_2_) the catalyst was filtered-off over celite and washed with MeOH. The solvent was removed under reduced pressure and the crude product purified by flash column chromatography (10% MeOH in DCM) to yield the diamino derivative **56b** (270 mg, 95%) as a yellowish solid. ^1^H NMR (600 MHz, DMSO-*d*_6_) δ [ppm] = 6.86 (t, *J* = 7.7 Hz, 1H), 5.98 (dd, *J* = 7.9, 2.9 Hz, 1H), 5.73 (br s, 2H), 5.13 (br s, 2H). ^13^C NMR (151 MHz, DMSO-*d*_6_) δ [ppm] = 155.1 (d, *J* = 222.7 Hz), 147.1 (d, *J* = 17.9 Hz), 125.2 (d, *J* = 3.1 Hz), 124.0 (d, *J* = 6.9 Hz), 93.7 (d, *J* = 38.6 Hz). LC-MS (m/z): 127.8 [M+H]^+^. Purity (HPLC-UV 254 nm-ESI-MS): 97.6%.

*6-Fluoro-1H-imidazo[4,5-b]pyridin-2-amine (****57a****).* Compound **57a** was synthesized as described in General procedure I. Yield: 63%, brown solid. ^1^H NMR (500 MHz, DMSO-*d*_6_) δ [ppm] = 8.01 (m, 1H), 7.78 (s, 2H), 7.52 (dd, *J* = 9.0, 2.6 Hz, 1H). ^13^C NMR (126 MHz, DMSO-*d*_6_) δ [ppm] = 156.1 (d, *J* = 240.7 Hz), 155.2, 145.1, 128.2 (d, *J* = 9.7 Hz), 127.6 (d, *J* = 27.9 Hz), 106.79 (d, *J* = 25.0 Hz). LC-MS (m/z): 152.8 [M+H]^+^. Purity (HPLC-UV 254 nm-ESI-MS): 97.3%.

*5-Fluoro-1H-imidazo[4,5-b]pyridin-2-amine (****57b****).* Compound **57b** was synthesized as described in General procedure I. Yellowish solid. ^1^H NMR (600 MHz, DMSO-*d*_6_) δ [ppm] = 7.62 (dd, *J* = 8.2, 7.2 Hz, 1H), 7.49 (s, 2H), 6.68 (d, *J* = 8.2 Hz, 1H). ^13^C NMR (151 MHz, DMSO-*d*_6_) δ [ppm] = 158.7 (d, *J* = 228.9 Hz), 155.4, 148.9 (d, *J* = 20.2 Hz), 123.9 (d, *J* = 2.6 Hz), 121.1 (d, *J* = 9.6 Hz), 99.3 (d, *J* = 39.8 Hz). LC-MS (m/z): 152.9 [M+H]^+^. Purity (HPLC-UV 254 nm-ESI-MS): 97.5%.

*7-Ethyl-3-fluoro-8-isopropylpyrido[2',3':4,5]imidazo[1,2-a]pyrimidin-6(9H)-one (****32****).* Compound **32** was synthesized as described in General procedure J. Yield: 10%, white solid; mp 212 - 215 °C. ^1^H NMR (600 MHz, DMSO-*d*_6_) δ [ppm] = 12.91 (br s, 1H), 8.43 (t, *J* = 2.4 Hz, 1H,), 8.41 (dd, *J* = 8.3, 2.9 Hz, 1H), 3.27 - 3.20 (m, 1H), 2.59 (q, *J* = 7.5 Hz, 2H), 1.26 (d, *J* = 6.7 Hz, 6H), 1.07 (t, *J* = 7.4 Hz, 3H). ^13^C NMR (151 MHz, DMSO-*d*_6_) δ [ppm] = 159.5, 154.6, 149.2, 149.1, 133.4, 120.0, 119.9, 110.3, 110.2, 30.2, 21.1, 17.8, 14.6. HRMS (ESI-QTOF) calculated for C_14_H_15_FN_4_O [M+H]^+^: 275.1303; found: 275.1320.

*7-Ethyl-2-fluoro-8-isopropylpyrido[2',3':4,5]imidazo[1,2-a]pyrimidin-6(9H)-one (****33****).* Compound **33** was synthesized as described in General procedure J. Yield: 14% over 2 steps, yellowish solid; mp >300 °C. ^1^H NMR (600 MHz, DMSO-*d*_6_) δ [ppm] = 12.73 (br s, 1H), 8.65 (t, *J* = 7.9 Hz, 1H), 6.97 (d, *J* = 8.4 Hz, 1H), 3.30 - 3.25 (m, 1H), 2.59 (q, *J* = 7.4 Hz, 2H), 1.28 (d, *J* = 6.8 Hz, 6H), 1.07 (t, *J* = 7.4 Hz, 3H). ^13^C NMR (126 MHz, DMSO-*d*_6_) δ [ppm] = 160.6 (d, *J* = 233.2 Hz), 159.0, 126.7, 100.8 (d, *J* = 40.7 Hz), 29.5, 20.5, 17.6, 14.4. HRMS (ESI-QTOF) calculated for C_14_H_15_FN_4_O [M+H]^+^: 275.1303; found: 275.1320.

Data S 2: Homology model of MRGPRX2

In order to explore receptor-ligand interactions and rationalize the SARs of the new MRGPRX2 antagonists, a homology model of the human MRGPRX2 was generated on the basis of the crystal structure of the human P2Y_12_ receptor in complex with the antagonist AZD1283 (PDB: 4NTJ).^4^ Lansu *et. al.* identified opioid-related MRGPRX2 agonists including the drugs morphine, hydrocodone and dextromethorphan.^5^ The authors subsequently generated a homology model of the human MRGPRX2 based on the X-ray structure of the human κ-opioid receptor in complex with a selective antagonist, JDTic (PDB 4DJH) as a template and searched for novel agonists using a structure-based virtual screening approach.^6^ The selected template, the κ-opioid receptor, belongs to the *γ*-branch of the class A GPCR family and shares a sequence similarity of 39.6% with the human MRGPRX2. By virtual screening of the ZINC database, ZINC-3573 was identified as a novel MRGPRX2 agonist with an EC_50_ value of 760 nM.^5^ In the present s, the P2Y_12_ receptor structure was selected as a more closely related template with a sequence similarity of 43.3% belonging also to the δ-branch of class A GPCR family as MRGPRX2.

The overall root mean square deviation (RMSD) of the two homology models of MRGPRX2 was 4.6 Å. The comparison of the amino acid residues in the putative orthosteric binding pocket resulted in an RMSD value of 3.8 Å. As a next step, we explored the conformation of the residues in the binding pocket and their interaction by docking the agonist ZINC-3573.

References

(1) Puttaraju, K. B.; Shivashankar, K.; Chandra; Mahendra, M.; Rasal, V. P.; Venkata Vivek, P. N.; Rai, K.; Chanu, M. B. Microwave assisted synthesis of dihydrobenzo4,5imidazo1,2-apyrimidin-4-ones; synthesis, in vitro antimicrobial and anticancer activities of novel coumarin substituted dihydrobenzo4,5imidazo1,2-apyrimidin-4-ones. *European journal of medicinal chemistry* **2013**, *69*, 316–322.

(2) Sirko, S. M.; Gorobets, N. Y.; Musatov, V. I.; Desenko, S. M. Generation of 500-member library of 10-alkyl-2-R(1),3-R(2)-4,10-dihydrobenzo4,5imidazo1,2-alphapyrimidin-4-ones. *Molecules (Basel, Switzerland)* **2009**, *14*, 5223–5234.

(3) Verdonck, S.; Herdewyn, P.; Jonghe, S. de. *Synthesis and biological evaluation of heterocyclic structures targeting underexplored targets*, 2020.

(4) Zhang, K.; Zhang, J.; Gao, Z.-G.; Zhang, D.; Zhu, L.; Han, G. W.; Moss, S. M.; Paoletta, S.; Kiselev, E.; Lu, W.*; et al.* Structure of the human P2Y12 receptor in complex with an antithrombotic drug. *Nature* **2014**, *509*, 115–118.

(5) Lansu, K.; Karpiak, J.; Liu, J.; Huang, X.-P.; McCorvy, J. D.; Kroeze, W. K.; Che, T.; Nagase, H.; Carroll, F. I.; Jin, J.*; et al.* In silico design of novel probes for the atypical opioid receptor MRGPRX2. *Nat. Chem. Biol.* **2017**, *13*, 529–536.

(6) Wu, H.; Wacker, D.; Katritch, V.; Mileni, M.; Han, G. W.; Vardy, E.; Liu, W.; Thompson, A. A.; Huang, X.-P.; Carroll, F. I.*; et al.* Structure of the human kappa opioid receptor in complex with JDTic. *Nature* **2012**, *485*, 327–332.
