# Supplementary material for: Subnanomolar MAS-related G protein-coupled receptor-X2/B2 antagonists with efficacy in human mast cells and disease models
Source: Signal Transduct Target Ther. 2025 Apr 21;10:128. doi: 10.1038/s41392-025-02209-8 (PMC12010006; doi:10.1038/s41392-025-02209-8)
Supplement: Supplementary file 3 — Supplementary Data S2 [file 41392_2025_2209_MOESM3_ESM.docx]

Data S 2: Homology model of MRGPRX2

In order to explore receptor-ligand interactions and rationalize the SARs of the new MRGPRX2 antagonists, a homology model of the human MRGPRX2 was generated on the basis of the crystal structure of the human P2Y_12_ receptor in complex with the antagonist AZD1283 (PDB: 4NTJ).^4^ Lansu *et. al.* identified opioid-related MRGPRX2 agonists including the drugs morphine, hydrocodone and dextromethorphan.^5^ The authors subsequently generated a homology model of the human MRGPRX2 based on the X-ray structure of the human κ-opioid receptor in complex with a selective antagonist, JDTic (PDB 4DJH) as a template and searched for novel agonists using a structure-based virtual screening approach.^6^ The selected template, the κ-opioid receptor, belongs to the *γ*-branch of the class A GPCR family and shares a sequence similarity of 39.6% with the human MRGPRX2. By virtual screening of the ZINC database, ZINC-3573 was identified as a novel MRGPRX2 agonist with an EC_50_ value of 760 nM.^5^ In the present s, the P2Y_12_ receptor structure was selected as a more closely related template with a sequence similarity of 43.3% belonging also to the δ-branch of class A GPCR family as MRGPRX2.

The overall root mean square deviation (RMSD) of the two homology models of MRGPRX2 was 4.6 Å. The comparison of the amino acid residues in the putative orthosteric binding pocket resulted in an RMSD value of 3.8 Å. As a next step, we explored the conformation of the residues in the binding pocket and their interaction by docking the agonist ZINC-3573.

References

(1) Puttaraju, K. B.; Shivashankar, K.; Chandra; Mahendra, M.; Rasal, V. P.; Venkata Vivek, P. N.; Rai, K.; Chanu, M. B. Microwave assisted synthesis of dihydrobenzo4,5imidazo1,2-apyrimidin-4-ones; synthesis, in vitro antimicrobial and anticancer activities of novel coumarin substituted dihydrobenzo4,5imidazo1,2-apyrimidin-4-ones. *European journal of medicinal chemistry* **2013**, *69*, 316–322.

(2) Sirko, S. M.; Gorobets, N. Y.; Musatov, V. I.; Desenko, S. M. Generation of 500-member library of 10-alkyl-2-R(1),3-R(2)-4,10-dihydrobenzo4,5imidazo1,2-alphapyrimidin-4-ones. *Molecules (Basel, Switzerland)* **2009**, *14*, 5223–5234.

(3) Verdonck, S.; Herdewyn, P.; Jonghe, S. de. *Synthesis and biological evaluation of heterocyclic structures targeting underexplored targets*, 2020.

(4) Zhang, K.; Zhang, J.; Gao, Z.-G.; Zhang, D.; Zhu, L.; Han, G. W.; Moss, S. M.; Paoletta, S.; Kiselev, E.; Lu, W.*; et al.* Structure of the human P2Y12 receptor in complex with an antithrombotic drug. *Nature* **2014**, *509*, 115–118.

(5) Lansu, K.; Karpiak, J.; Liu, J.; Huang, X.-P.; McCorvy, J. D.; Kroeze, W. K.; Che, T.; Nagase, H.; Carroll, F. I.; Jin, J.*; et al.* In silico design of novel probes for the atypical opioid receptor MRGPRX2. *Nat. Chem. Biol.* **2017**, *13*, 529–536.

(6) Wu, H.; Wacker, D.; Katritch, V.; Mileni, M.; Han, G. W.; Vardy, E.; Liu, W.; Thompson, A. A.; Huang, X.-P.; Carroll, F. I.*; et al.* Structure of the human kappa opioid receptor in complex with JDTic. *Nature* **2012**, *485*, 327–332.
